# Supplementary material for: Are pharmacists on the front lines of the opioid epidemic? A cross-sectional study of the practices and competencies of community and hospital pharmacists in Punjab, Pakistan
Source: BMJ Open. 2023 Nov 21;13(11):e079507. doi: 10.1136/bmjopen-2023-079507 (PMC10668153; doi:10.1136/bmjopen-2023-079507)
Supplement: Supplementary data [file bmjopen-2023-079507supp001.pdf]

"Are Pharmacists on the Frontlines of the Opioid Epidemic? A Cross Sectional Study of the Practices and Competencies of Community and Hospital Pharmacists in Punjab, Pakistan"

Naeem Mubarak,[1] Taheer Zahid,[2] Fatima Rahman Rana,[1] Umm-E-Barirah Ijaz,[1] Afshan Shabbir,[1] Mahrukh Manzoor,[1] Nahan Khan,[1] Minahil Arif,[1] Muhammad Mehroz Naeem,[1] Sabba Kanwal,[1] Nasira Saif-ur-Rehman,[3] Che Suraya Zin,[4] Khalid Mahmood,[5] Javaid Asgher,[1,6,7] Mohamed Hassan Elnaem[8*]

[1] Department of Pharmacy Practice, Faculty of Pharmaceutical Sciences, Lahore University of Biological & Applied Sciences, Lahore, Punjab, Pakistan.

[2] Manager, Servaid Pharmacy, 24-M Quaid-e-Azam Industrial Estate, Lahore, Punjab, Pakistan.

[3] Department of Pharmaceutics, Faculty of Pharmaceutical Sciences, Lahore University of Biological & Applied Sciences, Lahore, Punjab, Pakistan.

[4] Kulliyyah of Pharmacy, International Islamic University, Kuantan, Malaysia.

[5] Institute of Information Management, University of the Punjab, Lahore, Punjab, Pakistan.

[6] Lahore Medical & Dental College, Lahore, Punjab, Pakistan.

[7] Doctors Hospital & Medical Centre, Lahore, Punjab, Pakistan.

[8] School of Pharmacy and Pharmaceutical Sciences, Ulster University, Coleraine, United Kingdom.

Corresponding author:
Dr Mohamed Hassan Elnaem,
School of Pharmacy and Pharmaceutical Sciences, Ulster University, Coleraine, United Kingdom.
Email: m.elnaem@ulster.ac.uk
ORCiD: 0000-0003-0873-6541

STROBE Statement—checklist of items that should be included in reports of observational studies.

	Item No.	Recommendation	Page No.	Relevant text from manuscript
**Title and abstract**	1	(*a*) Indicate the study's design with a commonly used term in the title or the abstract	1	Line 1
		(*b*) Provide in the abstract an informative and balanced summary of what was done and what was found	2	Line 36-63
		**Introduction**		
Background/rationale	2	Explain the scientific background and rationale for the investigation being reported	4-5	Line 81-129
Objectives	3	State specific objectives, including any prespecified hypotheses	5	Line 128
		**Methods**		
Study design	4	Present key elements of study design early in the paper	5	Line 133
Setting	5	Describe the setting, locations, and relevant dates, including periods of recruitment, exposure, follow-up, and data collection	5-6	Line 132-149
Participants	6	(*a*) *Cohort study*—Give the eligibility criteria, and the sources and methods of selection of participants. Describe methods of follow-up<br>*Case-control study*—Give the eligibility criteria, and the sources and methods of case ascertainment and control selection. Give the rationale for the choice of cases and controls<br>*Cross-sectional study*—Give the eligibility criteria, and the sources and methods of selection of participants	7,16	Line 185-199, 440
		(*b*) *Cohort study*—For matched studies, give matching criteria and number of exposed and unexposed<br>*Case-control study*—For matched studies, give matching criteria and the number of controls per case	NA	NA
Variables	7	Clearly define all outcomes, exposures, predictors, potential confounders, and effect modifiers. Give diagnostic criteria, if applicable	8	Line 202-209
Data sources/ measurement	8*	For each variable of interest, give sources of data and details of methods of assessment (measurement). Describe comparability of assessment methods if there is more than one group	8	Line 202-209
Bias	9	Describe any efforts to address potential sources of bias	6	Line 147-149
Study size	10	Explain how the study size was arrived at	6	Line 143-146

Mubarak N, *et al. BMJ Open* 2024; 13:e079507. doi: 10.1136/bmjopen-2023-079507

Quantitative variables	11	Explain how quantitative variables were handled in the analyses. If applicable, describe which groupings were chosen and why	8,12	Line 202-209,273-277
Statistical methods	12	(*a*) Describe all statistical methods, including those used to control for confounding	8-13	Line 202-209,231-288
		(*b*) Describe any methods used to examine subgroups and interactions	8,12	Line 202-209,273-277
		(*c*) Explain how missing data were addressed	8	Line 199
		(*d*) *Cohort study*—If applicable, explain how loss to follow-up was addressed<br>*Case-control study*—If applicable, explain how matching of cases and controls was addressed.<br>*Cross-sectional study*—If applicable, describe analytical methods taking account of sampling strategy	5-6	Line 138-149
		(*e*) Describe any sensitivity analyses	NA	NA
		**Results**		
Participants	13*	(a) Report numbers of individuals at each stage of study—e.g., numbers potentially eligible, examined for eligibility, confirmed eligible, included in the study, completing follow-up, and analysed	5-6	Line 138-149
		(b) Give reasons for non-participation at each stage	8	Line 199
		(c) Consider use of a flow diagram	NA	NA
Descriptive data	14*	(a) Give characteristics of study participants (eg demographic, clinical, social) and information on exposures and potential confounders	9	Line 232-241
		(b) Indicate number of participants with missing data for each variable of interest	8	Line 199
		(c) *Cohort study*—Summarise follow-up time (eg, average and total amount)	NA	NA
Outcome data	15*	*Cohort study*—Report numbers of outcome events or summary measures over time	*NA*	*NA*
		Case-control study—Report numbers in each exposure category, or summary measures of exposure	*NA*	*NA*
		Cross-sectional study—Report numbers of outcome events or summary measures	8-13	Line 231-288
Main results	16	(*a*) Give unadjusted estimates and, if applicable, confounder-adjusted estimates and their precision (e.g., 95% confidence interval). Make clear which confounders were adjusted for and why they were included	8	Line 212-218
		(*b*) Report category boundaries when continuous variables were categorized	NA	NA
		(*c*) If relevant, consider translating estimates of relative risk into absolute risk for a meaningful time period	NA	NA

Continued on next page

Other analyses	17	Report other analyses done—e.g., analyses of subgroups and interactions, and sensitivity analyses	13	Line 279
		**Discussion**		
Key results	18	Summarise key results with reference to study objectives	13	Line 291
Limitations	19	Discuss limitations of the study, taking into account sources of potential bias or imprecision. Discuss both direction and magnitude of any potential bias	6,18	Line 147-149,410-417
Interpretation	20	Give a cautious overall interpretation of results considering objectives, limitations, multiplicity of analyses, results from similar studies, and other relevant evidence	13-18	Line 291-417
Generalisability	21	Discuss the generalisability (external validity) of the study results	3	Line 72,78
**Other information**				
Funding	22	Give the source of funding and the role of the funders for the present study and, if applicable, for the original study on which the present article is based	20	Line 471

*Give information separately for cases and controls in case-control studies and, if applicable, for exposed and unexposed groups in cohort and cross-sectional studies.

Note: An Explanation and Elaboration article discusses each checklist item and gives methodological background and published examples of transparent reporting. The STROBE checklist is best used in conjunction with this article (freely available on the Web sites of PLoS Medicine at http://www.plosmedicine.org/, Annals of Internal Medicine at http://www.annals.org/, and Epidemiology at http://www.epidem.com/). Information on the STROBE Initiative is available at www.strobe-statement.org.

Mubarak N, *et al. BMJ Open* 2024; 13:e079507. doi: 10.1136/bmjopen-2023-079507
