# Supplementary material for: Are pharmacists on the front lines of the opioid epidemic? A cross-sectional study of the practices and competencies of community and hospital pharmacists in Punjab, Pakistan
Source: BMJ Open. 2023 Nov 21;13(11):e079507. doi: 10.1136/bmjopen-2023-079507 (PMC10668153; doi:10.1136/bmjopen-2023-079507)
Supplement: Supplementary data [file bmjopen-2023-079507supp002.pdf]

"Are Pharmacists on the Frontlines of the Opioid Epidemic? A Cross Sectional Study of the Practices and Competencies of Community and Hospital Pharmacists in Punjab, Pakistan"

Naeem Mubarak,[1] Taheer Zahid,[2] Fatima Rahman Rana,[1] Umm-E-Barirah Ijaz,[1] Afshan Shabbir,[1] Mahrukh Manzoor,[1] Nahan Khan,[1] Minahil Arif,[1] Muhammad Mehroz Naeem,[1] Sabba Kanwal,[1] Nasira Saif-ur-Rehman,[3] Che Suraya Zin,[4] Khalid Mahmood,[5] Javaid Asgher,[1,6,7] Mohamed Hassan Elnaem[8*]

[1] Department of Pharmacy Practice, Faculty of Pharmaceutical Sciences, Lahore University of Biological & Applied Sciences, Lahore, Punjab, Pakistan.

[2] Manager, Servaid Pharmacy, 24-M Quaid-e-Azam Industrial Estate, Lahore, Punjab, Pakistan.

[3] Department of Pharmaceutics, Faculty of Pharmaceutical Sciences, Lahore University of Biological & Applied Sciences, Lahore, Punjab, Pakistan.

[4] Kulliyyah of Pharmacy, International Islamic University, Kuantan, Malaysia.

[5] Institute of Information Management, University of the Punjab, Lahore, Punjab, Pakistan.

[6] Lahore Medical & Dental College, Lahore, Punjab, Pakistan.

[7] Doctors Hospital & Medical Centre, Lahore, Punjab, Pakistan.

[8] School of Pharmacy and Pharmaceutical Sciences, Ulster University, Coleraine, United Kingdom.

Corresponding author:
Dr Mohamed Hassan Elnaem,
School of Pharmacy and Pharmaceutical Sciences, Ulster University, Coleraine, United Kingdom.
Email: m.elnaem@ulster.ac.uk
ORCiD: 0000-0003-0873-6541

DEMOGRAPHICS

Please ENCIRCLE/WRITE

- **Gender:** Male Female
- **Age (years)** ________ **Years of practice experience** _________
- **Employment status:** Full-time Part-time Intern
- **Type of community pharmacy:** Chain Independent Hospital
- **If pharmacy in hospital, type of hospital:** Public Private
- **Highest level of education:** PhD Masters Pharm-D/B-Pharm
- **Location of pharmacy:**
  - Lahore, Kasur, Nankana Sahib, Sheikhupura
  - Gujranwala, Gujrat, Hafizabad, MandiBahauddin, Narowal, Sialkot
  - Sargodha, Khushab, Mianwali, Bhakkar
  - Sahiwal, Pakpattan, Okara
  - Multan, Lodhran, Khanewal, Vehari
  - Faisalabad, Chiniot, Toba Tek Singh, Jhang
  - Rawalpindi, Jhelum, Chakwal, Attock
  - Bahawalpur, Bahawalnagar, Rahim Yar Khan
  - DG Khan, Layyah, Muzaffargarh, Rajanpur
- **Please encircle the appropriate:**

[Never = 1, rarely= 2, Sometimes= 3, Very often= 4, Almost always =5]

- I……………..make clinical notes in a journal/dispensing software (apart from narcotic register) to monitor ongoing opioids use.
  1 2 3 4 5
- I …...counsel patients on the possible side effects of opioids.
  1 2 3 4 5
- I …...counsel patients on opioids over dose risk.
  1 2 3 4 5
- I ……….refuse the supply of the opioids when in doubt of abuse
  1 2 3 4 5
- I…………notify police/regulatory bodies on the abuse of opioids
  1 2 3 4 5
- To clarify the opioids' prescription, I …………call the physician

[Never = 1, rarely= 2, Sometimes= 3 Very often= 4 Almost always =5]

- **Have you completed any continuous professional development course in last ONE year?** YES NO
- **You completed your B.Pharm/Pharm-D degree from a ……….institute**
  Public Private
- **Your system of education in B.Pharm/Pharm-D was:**
  Annual Semester

OPIOIDS AND SOCIETY

1 **The "Opioid Epidemic" or "Opioid Crises" refers to the increase in the number of deaths and hospitalization:**
   a. Due to prescription opioid use
   b. Due to illicit(illegal) opioid use
   c. Due to opioid use for instant pain relief
   d. Due to both prescription and illicit(illegal) opioid use
   e. I don't know

2 **National Institute on Drug Abuse recommends the term _______ for a "substance or drug abuser"**
   a. Drug addict
   b. Patient
   c. Junkie
   d. Addicted baby
   e. I don't know

3 **In Pakistan, the highest number of opioid drug users belongs to:**
   a. KPK
   b. Punjab
   c. Balochistan
   d. Sindh
   e. I don't know

4 **In Pakistan, opioid misuse is more common among**
   a. Males
   b. Females
   c. Transgender
   d. No difference on the basis of gender
   e. I don't know

5 **In Pakistan, generally women abuse sedative and amphetamines, while, men favor _____**
   a. Methamphetamine and cannabis
   b. Cannabis and alcohol
   c. Sedative and opioids
   d. Cannabis and opioids
   e. I don't know

OPIOID PHYSIOLOGY

1 **Opioids produce analgesia, which indicates:**
   a. CNS depression
   b. CNS stimulation
   c. Both depression and stimulation
   d. No effect
   e. I don't know

2 **The increase in the sensitivity of pain receptors is called:**
   a. Analgesia
   b. Algesia
   c. Hyperalgesia
   d. Allodynia
   e. I don't know

3 **Beta endorphins are the most common endogenous ligands for the receptor:**
   a. Beta
   b. Delta
   c. Kappa
   d. Mu
   e. I don't know

4 **When activated, which one of the following opioid receptors mainly cause both analgesia and dysphoria?**
   a. Mu
   b. Kappa
   c. Delta
   d. Alpha
   e. I don't know

5 **When activated, which one of the following opioid receptors causes the most respiratory depression?**
   a. Mu
   b. Beta
   c. Kappa
   d. Delta
   e. I don't know

OPIOID PHARMACOLOGY

1 **The difference between Opiates and Opioids is**
   a. Opiates are natural, while Opioids are synthetic
   b. Opioids are natural, while Opiates are synthetic
   c. Opiates are semi-synthetic, while Opioids are synthetic
   d. Opioids are semi-synthetic, while Opiates are synthetic
   e. I don't know

2 **Majority of the opioid receptors belongs to:**
   a. Intracellular receptors
   b. Tyrosine Kinase receptors
   c. G-protein coupled receptors
   d. Enzyme linked receptors
   e. I don't know

3 **Tramadol is a __________ Mu receptor agonist**
   a. Strong
   b. Weak
   c. Moderate
   d. None of these
   e. I don't know

4 **At the injection site, Morphine causes localized pain and itching due to the release of:**
   a. Glutamine
   b. Serotonin
   c. Dopamine
   d. Histamine
   e. I don't know

5 **Which one of the following opioids is a mixed agonist-antagonist?**
   a. Morphine
   b. Fentanyl
   c. Codeine
   d. Pentazocine
   e. I don't know

Mubarak N, *et al. BMJ Open* 2024; 13:e079507. doi: 10.1136/bmjopen-2023-079507

OPIOID PHARMACOKINETICS AND DRUG INTERACTIONS

1 **Morphine undergoes _________ during phase-2 biotransformation**
   a. Methylation
   b. Acetylation
   c. Glucuronidation
   d. Oxidation
   e. I don't know

2 **Which one of the following opioids is safe to use in renal failure and does not require dose adjustment?**
   a. Buprenorphine
   b. Morphine
   c. Fentanyl
   d. Methadone
   e. I don't know

3 **Which one of the following opioids has the least CNS penetration?**
   a. Morphine
   b. Loperamide
   c. Codeine
   d. Fentanyl
   e. I don't know

4 **Opioids can cause significant respiratory depression when administered with:**
   a. Naloxone
   b. Naltrexone
   c. Anti-hypertensive
   d. Benzodiazepines
   e. I don't know

5 **Which of the following drugs when administered with tramadol can cause serotonin syndrome (increase in serotonin levels)?**
   a. Tricyclic anti-depressants
   b. Selective serotonin reuptake inhibitors
   c. Both
   d. None of these
   e. I don't know

THERAPEUTIC USE OF OPIOIDS

1 **The appropriate treatment for moderate pain as defined by WHO analgesic ladder is:**
   a. NSAIDS
   b. Strong opioids and NSAIDS
   c. Weak opioids and NSAIDS
   d. Weak opioids and strong opioids
   e. I don't know

2 **Which of the following opioids are used for opioid substitution therapy?**
   a. Meperidine and Methadone
   b. Oxycodone and Buprenorphine
   c. Fentanyl and Methadone
   d. Methadone and Buprenorphine
   e. I don't know

3 **Opioid dependence refers to:**
   a. Withdrawal symptoms when not taking the opioid
   b. Reduced response to an opioid with repeated use
   c. Persistent use of an opioid despite substantial harm and adverse consequences
   d. Overwhelming desire to continue taking the opioid and obtaining it by all means
   e. I don't know

4 **Which one of the following opioids is generally contraindicated in Myocardial Infarction?**
   a. Morphine
   b. Fentanyl
   c. Methadone
   d. Pentazocine
   e. I don't know

5 **Opioid dependence during pregnancy can be treated with:**
   a. Methadone
   b. Fentanyl
   c. Naloxone
   d. Morphine
   e. I don't know

Mubarak N, *et al. BMJ Open* 2024; 13:e079507. doi: 10.1136/bmjopen-2023-079507

OPIOID EDUCATION

1 **Pharmacist should educate the patient to change the fentanyl patch after:**
   a. 72 hours
   b. 48 hours
   c. 24 hours
   d. 6 hours
   e. I don't know

2 **The appropriate fate of fentanyl patch after use is:**
   a. Reuse the same patch
   b. Incinerate(fire) the patch
   c. Flush over (used or leftover patches)
   d. Give to someone else for use to prevent drug wastage
   e. I don't know

3 **Pharmacist should advise the patient to store opioids at:**
   a. Room temperature
   b. Refrigerator
   c. Freezer
   d. Any temperature
   e. I don't know

4 **Pharmacist should restrict methadone supply for:**
   a. 7 days
   b. 8 days
   c. 6 days
   d. 10 days
   e. I don't know

5 **In case of incorrect, ambiguous or incomplete prescription of opioids, pharmacist should:**
   a. Dispense medication as written
   b. Correct the prescription and dispense the medication
   c. Call to police
   d. Hold dispensing and clarify from the prescriber
   e. I don't know

OPIOID DISPENSING

1 **In Pakistan, as per Control of Narcotic Substances Act 1967, opioids come under:**
   a. Schedule A
   b. Schedule B
   c. Schedule C
   d. Schedule D
   e. I don't know

2 **Which one of the following is NOT the sign of opioid's intoxication?**
   a. Altered mental state
   b. Shallow breathing
   c. Irresponsiveness
   d. Fast breathing
   e. I don't know

3 **Pharmacist should dispense the take home doses of opioids in:**
   a. Child resistant containers
   b. Amber colored bottles
   c. Light colored containers
   d. Aluminum foil
   e. I don't know

4 **The record for the sale of opioids should be documented in the narcotic register with:**
   a. blue ink
   b. red ink
   c. green ink
   d. any color
   e. I don't know

5 **As per Punjab Drug Rules-2007, which one of the following is an important particular to mention in the narcotic register during the sale of opioids?**
   a. Lot number
   b. Expiry date
   c. Manufacturing date
   d. Batch number
   e. I don't know

OPIOIDS AND PAIN MANAGEMENT

1 **…is the pain due to injury to nerve and opioid of choice for this kind of pain is…………**
   a. Neuropathic, Methadone
   b. Psychogenic, Morphine
   c. Somatic, Methadone
   d. Visceral, Morphine
   e. I don't know

2 **Which one of the following opioids is the gold standard for the treatment of acute (severe, accidental) pain?**
   a. Methadone
   b. Fentanyl
   c. Morphine
   d. Tramadol
   e. I don't know

3 **Which one of the following is the opioid of choice in labor pain?**
   a. Meperidine
   b. Pentazocine
   c. Methadone
   d. Buprenorphine
   e. I don't know

4 **The Centers for Disease Control and Prevention recommends the use of opioid therapy in patients with acute (severe) pain for:**
   a. 7-14 days
   b. 3-7 days
   c. 14-28 days
   d. More than 1 month
   e. I don't know

5 **FDA approved opioid for treatment of acute pain in children is:**
   a. Morphine
   b. Codeine
   c. Fentanyl
   d. Methadone
   e. I don't know

COURSE OF OPIOID THERAPY

1 **Opioid taper, Opioid rotation, and Opioid Equianalgesic table refer to _____ , respectively:**
   a. Dose reduction over time, switching between opioids, equivalent to analgesic dose of morphine
   b. Switching between opioids, dose reduction over time , equivalent to analgesic dose of codeine
   c. Dose reduction over time, switching from opioids to non-opioids, equivalent to analgesic dose of morphine
   d. Dose reduction over time, switching between opioids, equivalent to analgesic dose of methadone
   e. I don't know

2 **The appropriate adult dose of Tramadol is:**
   a. 50-100mg for 4-6 hours PRN, with maximum dose up to 400mg/day
   b. 200-300mg for 4-6 hours PRN, with maximum dose up to 500mg/day
   c. 50-100mg for 4-6 hours PRN, with maximum dose up to 500mg/day
   d. 200-300mg for 4-6 hours PRN, with maximum dose up to 400mg/day
   e. I don't know

3 **As per equianalgesic table, _____ oral dose of tramadol is equivalent to 10mg of morphine**
   a. 100mg
   b. 50mg
   c. 200mg
   d. 150mg
   e. I don't know

4 **The indications for an opioid rotation are to:**
   a. Improve analgesia
   b. Reduce adverse effects
   c. Decrease opioid tolerance
   d. All of the above
   e. I don't know

5 **CDC guidelines recommend appropriate opioid tapering as the**
   a. 10% decrease in dose per month
   b. 20% decrease in dose per month
   c. 30% decrease in dose per month
   d. 40% decrease in dose per month
   e. I don't know

Mubarak N, *et al. BMJ Open* 2024; 13:e079507. doi: 10.1136/bmjopen-2023-079507

OPIOID MONITORING

1 **5A's of monitoring chronic pain are:**
   a. Activity, analgesia, adverse effects, aberrant behaviors, affect
   b. Activity, algesia, adverse effects, aberrant behaviors, assign
   c. Activity, algesia, adverse effects, adherent behaviors, affect
   d. Action, analgesia, adverse effects, aberrant behaviors, assign
   e. I don't know

2 **The bolus injection of morphine requires monitoring of respiratory rate after every:**
   a. 12 hours
   b. 6 hours
   c. 3 hours
   d. 24 hours
   e. I don't know

3 **Opioid therapy monitoring is recommended ……for patients at <u>low risk</u> of adverse effects:**
   a. Once every 3-6 months
   b. Twice every 3-6 months
   c. Monthly
   d. Weekly
   e. I don't know

4 **Opioid therapy monitoring is recommended ……..for patients at <u>high risk</u> of adverse effects:**
   a. Once every 3-6 months
   b. Twice every 3-6 months
   c. Monthly
   d. Weekly
   e. I don't know

5 **Chronic opioid therapy is the use of opioids daily or nearly daily for <u>at least</u>:**
   a. 30 days
   b. 90 days
   c. 60 days
   d. 180 days
   e. I don't know

OPIOID OVERDOSE TRAINING

1 **The maximum toxic dose of morphine in adult patients is:**
   a. 250mg/day
   b. 500mg/day
   c. 1000mg/day
   d. 100mg/day
   e. I don't know

2 **The usual dose of Naloxone for opioid toxicity is:**
   a. 0.4mg to 2mg IV at 2-3 min intervals
   b. 10 mg IV at 2-3 min intervals
   c. 2.5mg to 5mg at 2-3 min intervals
   d. 7.5 mg IV at 2-3 min intervals
   e. I don't know

3 **The FDA recommends administering IM Naloxone in the**
   a. Upper arm
   b. Buttock
   c. Veins
   d. Intrathecal
   e. I don't know

4 **Three key symptoms of opioid overdose triad include**:
   a. Pinpoint pupils, Slow breathing, Unconsciousness/non-responsiveness
   b. Dilated pupils, Shallow breathing, Unconsciousness/ non-responsiveness
   c. Pinpoint pupils, Fast breathing, Clammy skin
   d. Dilated pupils, Slow breathing, Limp body (difficulty in walk)
   e. I don't know

5 **Which one of the following opioids is resistant to Naloxone?**
   a. Buprenorphine
   b. Morphine
   c. Codeine
   d. Hydromorphone
   e. I don't know

Mubarak N, *et al. BMJ Open* 2024; 13:e079507. doi: 10.1136/bmjopen-2023-079507
