# Supplementary material for: Are pharmacists on the front lines of the opioid epidemic? A cross-sectional study of the practices and competencies of community and hospital pharmacists in Punjab, Pakistan
Source: BMJ Open. 2023 Nov 21;13(11):e079507. doi: 10.1136/bmjopen-2023-079507 (PMC10668153; doi:10.1136/bmjopen-2023-079507)
Supplement: Supplementary data [file bmjopen-2023-079507supp004.pdf]

"Are Pharmacists on the Frontlines of the Opioid Epidemic? A Cross Sectional Study of the Practices and Competencies of Community and Hospital Pharmacists in Punjab, Pakistan"

Naeem Mubarak,[1] Taheer Zahid,[2] Fatima Rahman Rana,[1] Umm-E-Barirah Ijaz,[1] Afshan Shabbir,[1] Mahrukh Manzoor,[1] Nahan Khan,[1] Minahil Arif,[1] Muhammad Mehroz Naeem,[1] Sabba Kanwal,[1] Nasira Saif-ur-Rehman,[3] Che Suraya Zin,[4] Khalid Mahmood,[5] Javaid Asgher,[1,6,7] Mohamed Hassan Elnaem[8*]

[1] Department of Pharmacy Practice, Faculty of Pharmaceutical Sciences, Lahore University of Biological & Applied Sciences, Lahore, Punjab, Pakistan.

[2] Manager, Servaid Pharmacy, 24-M Quaid-e-Azam Industrial Estate, Lahore, Punjab, Pakistan.

[3] Department of Pharmaceutics, Faculty of Pharmaceutical Sciences, Lahore University of Biological & Applied Sciences, Lahore, Punjab, Pakistan.

[4] Kulliyyah of Pharmacy, International Islamic University, Kuantan, Malaysia.

[5] Institute of Information Management, University of the Punjab, Lahore, Punjab, Pakistan.

[6] Lahore Medical & Dental College, Lahore, Punjab, Pakistan.

[7] Doctors Hospital & Medical Centre, Lahore, Punjab, Pakistan.

[8] School of Pharmacy and Pharmaceutical Sciences, Ulster University, Coleraine, United Kingdom.

Corresponding author:
Dr Mohamed Hassan Elnaem,
School of Pharmacy and Pharmaceutical Sciences, Ulster University, Coleraine, United Kingdom.
Email: m.elnaem@ulster.ac.uk
ORCiD: 0000-0003-0873-6541

S1 Table Cronbach's alpha value against each of the eleven opioid competencies

Sr.	Competency	Alpha*
**1.**	Opioids & Society	0.654
**2.**	Opioids' Physiology	0.800
**3.**	Opioids' Pharmacology	0.776
**4.**	Opioids' Pharmacokinetic & Drug Interaction	0.826
**5.**	Therapeutic use of Opioids	0.746
**6.**	Opioids' Education	0.740
**7.**	Opioids Dispensing	0.833
**8.**	Opioids & Pain Management	0.852
**9.**	Course of Opioid Therapy	0.844
**10.**	Opioids' Monitoring	0.871
**11.**	Opioid Overdose Management	0.852

*** Cronbach's alpha coefficient test**
