# Supplementary material for: Are pharmacists on the front lines of the opioid epidemic? A cross-sectional study of the practices and competencies of community and hospital pharmacists in Punjab, Pakistan
Source: BMJ Open. 2023 Nov 21;13(11):e079507. doi: 10.1136/bmjopen-2023-079507 (PMC10668153; doi:10.1136/bmjopen-2023-079507)
Supplement: Supplementary data [file bmjopen-2023-079507supp005.pdf]

"Are Pharmacists on the Frontlines of the Opioid Epidemic? A Cross Sectional Study of the Practices and Competencies of Community and Hospital Pharmacists in Punjab, Pakistan"

Naeem Mubarak,[1] Taheer Zahid,[2] Fatima Rahman Rana,[1] Umm-E-Barirah Ijaz,[1] Afshan Shabbir,[1] Mahrukh Manzoor,[1] Nahan Khan,[1] Minahil Arif,[1] Muhammad Mehroz Naeem,[1] Sabba Kanwal,[1] Nasira Saif-ur-Rehman,[3] Che Suraya Zin,[4] Khalid Mahmood,[5] Javaid Asgher,[1,6,7] Mohamed Hassan Elnaem[8*]

[1] Department of Pharmacy Practice, Faculty of Pharmaceutical Sciences, Lahore University of Biological & Applied Sciences, Lahore, Punjab, Pakistan.

[2] Manager, Servaid Pharmacy, 24-M Quaid-e-Azam Industrial Estate, Lahore, Punjab, Pakistan.

[3] Department of Pharmaceutics, Faculty of Pharmaceutical Sciences, Lahore University of Biological & Applied Sciences, Lahore, Punjab, Pakistan.

[4] Kulliyyah of Pharmacy, International Islamic University, Kuantan, Malaysia.

[5] Institute of Information Management, University of the Punjab, Lahore, Punjab, Pakistan.

[6] Lahore Medical & Dental College, Lahore, Punjab, Pakistan.

[7] Doctors Hospital & Medical Centre, Lahore, Punjab, Pakistan.

[8] School of Pharmacy and Pharmaceutical Sciences, Ulster University, Coleraine, United Kingdom.

Corresponding author:
Dr Mohamed Hassan Elnaem,
School of Pharmacy and Pharmaceutical Sciences, Ulster University, Coleraine, United Kingdom.
Email: **m.elnaem@ulster.ac.uk**
ORCiD: **0000-0003-0873-6541**

S2 Table Details the individual competency scores against different variables.

Variable	Category	Opioids & Society (C1)		Opioids' Physiology (C2)		Opioids' Pharmacology (C3)		Opioids' Pharmacokinetic & Drug Interaction (C4)		Therapeutic use of Opioids (C5)		Opioids' Education (C6)		Opioids Dispensing (C7)		Opioids & Pain Management (C8)		Course of Opioid Therapy (C9)		Opioids' Use monitoring (C10)		Opioid Overdose Management (C11)	
		Mean (SD)	t (p-value)	Mean (SD)	t (p-value)	Mean (SD)	t (p-value)	Mean (SD)	t (p-value)	Mean (SD)	t (p-value)	Mean (SD)	t (p-value)	Mean (SD)	t (p-value)	Mean (SD)	t (p-value)	Mean (SD)	t (p-value)	Mean (SD)	t (p-value)	Mean (SD)	t (p-value)
**Gender**	**Male**	1.45 (0.956)	-0.160 (0.873)	2.05 (1.353)	-0.966 (0.335)	1.95 (1.389)	-1.649 (0.100)	1.88 (1.280)	0.250 (0.803)	1.36 (1.198)	-1.329 (0.184)	2.17 (1.205)	-1.030 (0.303)	2.18 (1.352)	-1.200 (0.231)	1.78 (1.115)	0.048 (0.962)	1.91 (1.376)	1.620 (0.106)	1.42 (1.049)	-0.251 (0.802)	1.49 (1.316)	-0.589 (0.556)
	**Female**	1.46 (0.957)		2.15 (1.388)		2.12 (1.442)		1.86 (1.239)		1.48 (1.244)		2.27 (1.219)		2.30 (1.307)		1.77 (1.083)		1.75 (1.374)		1.44 (1.079)		1.55 (1.286)	
**System of assessment in the Pharm-D degree program**	**Annual**	1.49 (0.995)	1.054 (0.292)	2.17 (1.379)	1.639 (0.102)	2.05 (1.409)	0.826 (0.409)	1.90 (1.257)	0.608 (0.543)	1.39 (1.215)	-0.364 (0.716)	2.22 (1.210)	0.225 (0.822)	2.28 (1.285)	1.104 (0.270)	1.85 (1.098)	1.869 (0.062)	1.87 (1.474)	0.348 (0.728)	1.46 (1.074)	0.735 (0.462)	1.57 (1.350)	1.235 (0.219)
	**Semester**	1.42 (0.919)		2.01 (1.351)		1.97 (1.411)		1.85 (1.273)		1.42 (1.216)		2.20 (1.211)		2.17 (1.379)		1.71 (1.104)		1.84 (1.287)		1.40 (1.047)		1.46 (1.263)	
**Type of institute**	**Public**	1.49 (0.955)	1.144 (0.253)	2.16 (1.339)	1.625 (0.105)	2.02 (1.372)	0.248 (0.805)	1.96 (1.322)	2.066 **(0.039)** *	1.42 (1.244)	0.461 (0.645)	2.21 (1.221)	0.065 (0.948)	1.22 (1.351)	-0.082 (0.935)	1.89 (1.118)	2.939 **(0.003)** *	1.92 (1.479)	1.315 (0.189)	1.49 (1.096)	1.696 (0.090)	1.58 (1.337)	1.573 (0.116)
	**Private**	1.41 (0.956)		2.01 (1.390)		2.00 (1.196)		1.78 (1.196)		1.38 (1.185)		1.21 (1.200)		1.22 (1.322)		1.66 (1.075)		1.79 (1.258)		1.36 (0.016)		1.43 (0.268)	
**Highest level of education**	**Masters**	1.57 (0.960)	1.666 (0.096)	2.43 (1.353)	3.475 **(0.001)** *	2.24 (1.469)	2.259 **(0.024)** *	2.02 (1.351)	1.618 (0.106)	1.85 (1.244)	5.118 **(0.000)** *	2.41 (1.288)	2.173 **(0.031)** *	2.32 (1.371)	1.004 (0.316)	1.92 (1.137)	1.877 (0.061)	2.08 (1.494)	2.189 **(0.030)** *	1.62 (1.065)	2.539 **(0.011)** *	1.55 (1.315)	0.413 (0.680)
	**Pharm-D/ B-Pharm**	1.43 (0.953)		2.00 (1.357)		1.95 (1.390)		1.84 (1.241)		1.30 (1.184)		2.16 (1.186)		2.20 (1.327)		1.74 (1.092)		1.80 (1.341)		1.38 (0.053)		1.50 (1.303)	
**Have you completed any continuous professional development courses in last TWO years?**	**Yes**	1.53 (0.966)	1.230 (0.219)	2.03 (1.385)	-0.586 (0.558)	2.01 (1.526)	0.012 (0.991)	1.94 (1.317)	0.851 (0.395)	1.49 (1.197)	1.008 (0.314)	2.20 (1.239)	-0.103 (0.896)	2.25 (1.440)	0.327 (0.744)	1.76 (1.108)	-0.154 (0.878)	1.75 (1.445)	-1.180 (0.238)	1.40 (1.051)	-0.444 (0.658)	1.33 (1.259)	-2.113 **(0.035)** *
	**No**	1.43 (0.952)		2.10 (1.360)		2.01 (1.375)		1.85 (1.250)		1.38 (1.220)		2.21 (1.203)		2.21 (1.305)		1.78 (1.102)		1.88 (1.356)		1.44 (1.063)		1.56 (1.314)	
**Employment status**	**Full-time**	1.50 (0.955)	2.520 **(0.012)** *	2.14 (1.350)	2.211 **(0.027)** *	2.08 (1.398)	2.581 **(0.010)** *	1.96 (1.264)	3.768 **(0.000)** *	1.46 (1.228)	2.412 **(0.017)** *	2.29 (1.196)	3.483 **(0.001)** *	2.31 (1.350)	3.875 **(0.000)** *	1.82 (1.094)	2.116 **(0.035)** *	1.91 (1.390)	2.162 **(0.031)** *	1.50 (1.076)	3.803 **(0.000)** *	1.55 (1.324)	1.537 (0.125)
	**Part-time**	1.29 (0.941)		1.88 (1.405)		1.76 (1.428)		1.55 (1.219)		1.21 (1.150)		1.92 (1.220)		1.89 (1.232)		1.62 (1.123)		1.65 (1.310)		1.17 (0.958)		1.38 (1.229)	
**If pharmacy is in hospital, type of hospital:**	**Public**	1.67 (0.943)	2.628 **(0.009)** *	2.34 (1.344)	1.417 (0.157)	2.17 (1.429)	0.783 (0.434)	2.02 (1.490)	1.053 (0.293)	1.59 (1.269)	1.242 (0.215)	2.20 (1.292)	-0.282 (0.778)	1.91 (1.233)	-1.126 (0.261)	1.95 (1.181)	1.056 (0.292)	2.15 (1.586)	1.719 (0.087)	1.48 (1.023)	0.620 (0.536)	1.58 (1.306)	0.372 (0.710)
	**Private**	1.35 (0.988)		2.10 (1.284)		2.02 (1.475)		1.84 (1.252)		1.39 (1.195)		2.25 (1.323)		2.10 (1.367)		1.79 (1.043)		1.84 (1.345)		1.39 (1.167)		1.52 (1.253)	
**Type of pharmacy practice**	**Community**	1.39 (0.944)	-2.389 **(0.017)** *	1.99 (1.379)	-2.620 **(0.009)** *	1.95 (1.389)	-1.598 (0.111)	1.83 (1.172)	-1.322 (0.187)	1.34 (1.194)	-2.033 **(0.042)** *	2.20 (1.159)	-0.174 (0.862)	2.36 (1.349)	3.847 **(0.000)** *	1.71 (1.078)	-2.312 **(0.021)** *	1.75 (1.283)	-2.803 **(0.005)** *	1.41 (1.054)	-0.422 (0.673)	1.48 (1.315)	-0.848 (0.397)
	**Hospital**	1.56 (0.968)		2.26 (1.327)		2.12 (1.443)		1.96 (1.416)		1.52 (1.246)		2.22 (1.300)		1.97 (1.279)		1.90 (1.138)		2.05 (1.515)		1.45 (1.071)		1.56 (1.287)	

***Independent t-test, p-value (≥ 0.05) Bold values indicate statistical significance.**
SD= standard deviation, C= competency
