# Supplementary material for: Are pharmacists on the front lines of the opioid epidemic? A cross-sectional study of the practices and competencies of community and hospital pharmacists in Punjab, Pakistan
Source: BMJ Open. 2023 Nov 21;13(11):e079507. doi: 10.1136/bmjopen-2023-079507 (PMC10668153; doi:10.1136/bmjopen-2023-079507)
Supplement: Supplementary data [file bmjopen-2023-079507supp003.pdf]

# LAHORE PHARMACY COLLEGE

(A PROJECT OF LMDC)
**Affiliated with**
**UNIVERSITY OF HEALTH SCIENCES, LAHORE**

Ref. No. LPC / ETH/26/09/22 Date: 23-09-2022

**Dr. Naeem Mubarak,**
Department of Pharmacy Practice,
Lahore Pharmacy College,
*(A Project of LMDC).*

**SUBJECT:** **ETHICAL APPROVAL CERTIFICATE**

Dear Dr. Naeem Mubarak,

Apropos of the ethical review application (ref: NM/03//22), after a detailed review of the ethical aspects of the research project mentioned below, the Research Ethics Committee of Lahore Pharmacy College grants ethical approval for the implementation of the study.

- **Research Title:** "Evaluating competencies of Community Pharmacists when supplying opioids. A cross sectional study in Punjab, Pakistan".
- **Principal Supervisor:** Dr. Naeem Mubarak
- **Co-Supervisors:** Prof. Dr. Nasira Saif ur Rehman

This ethical approval certificate is valid from **23rd Sep. 2022** to **23rd Sep. 2023.**

Yours Truly,

**Dr. Asad Majeed Khan (PhD)**
Chairperson,
Research Ethics Committee,
Lahore Pharmacy College,
University of Health Sciences,
Lahore, Pakistan.
**asad.majeed@lmdc.edu.pk**

Tulspura, North Canal Bank, Lahore 53400, Pakistan | Tel: +92 346 4418891-98
Fax: +92 42 37392220 | E-mail: laphco@gmail.com | www.lmdc.edu.pk
