# Supplementary material for: Are pharmacists on the front lines of the opioid epidemic? A cross-sectional study of the practices and competencies of community and hospital pharmacists in Punjab, Pakistan
Source: BMJ Open. 2023 Nov 21;13(11):e079507. doi: 10.1136/bmjopen-2023-079507 (PMC10668153; doi:10.1136/bmjopen-2023-079507)
Supplement: Author's manuscript [file bmjopen-2023-079507.draft_revisions.pdf]

BMJ Open

BMJ Open is committed to open peer review. As part of this commitment we make the peer review history of every article we publish publicly available.

When an article is published we post the peer reviewers' comments and the authors' responses online. We also post the versions of the paper that were used during peer review. These are the versions that the peer review comments apply to.

The versions of the paper that follow are the versions that were submitted during the peer review process. They are not the versions of record or the final published versions. They should not be cited or distributed as the published version of this manuscript.

BMJ Open is an open access journal and the full, final, typeset and author-corrected version of record of the manuscript is available on our site with no access controls, subscription charges or pay-per-view fees (http://bmjopen.bmj.com).

If you have any questions on BMJ Open's open peer review process please email info.bmjopen@bmj.com

BMJ Open

"Are Pharmacists at the Frontline? A Cross Sectional Analysis of the Practices and Competencies of Community and Hospital Pharmacists Amid the Looming Opioid Crises"

Journal:	*BMJ Open*
Manuscript ID	bmjopen-2023-079507
Article Type:	Original research
Date Submitted by the Author:	02-Sep-2023

Complete List of Authors:	MUBARAK, NAEEM; Lahore Medical and Dental College,<br>Zahid, Taheer; Lahore Medical and Dental College<br>Rana, Fatima Rahman; Lahore Medical and Dental College<br>Ijaz, Umm-E-Barirah; Lahore Medical and Dental College<br>Shabbir, Afshan; Lahore Medical and Dental College<br>Manzoor, Mahrukh; Lahore Medical and Dental College<br>Khan, Nahan; Lahore Medical and Dental College<br>Arif, Minahil; Lahore Medical and Dental College<br>Naeem, Muhammad Mehroz; Lahore Medical and Dental College<br>Kanwal, Sabba; Lahore Medical and Dental College<br>Saif-ur-Rehman, Nasira; Lahore Medical and Dental College<br>Zin, Che; International Islamic University Malaysia - Kuantan Campus, Pharmacy Practice<br>Mahmood, Khalid; University of the Punjab Quaid-i-Azam Campus<br>Asgher, Javaid; Lahore Medical and Dental College<br>Elnaem, Mohamed Hassan; Ulster University - Coleraine Campus

Keywords:	Hospitals, PAIN MANAGEMENT, Primary Care < Primary Health Care, Surveys and Questionnaires, PUBLIC HEALTH

SCHOLARONE™
Manuscripts

I, the Submitting Author has the right to grant and does grant on behalf of all authors of the Work (as defined in the below author licence), an exclusive licence and/or a non-exclusive licence for contributions from authors who are: i) UK Crown employees; ii) where BMJ has agreed a CC-BY licence shall apply, and/or iii) in accordance with the terms applicable for US Federal Government officers or employees acting as part of their official duties; on a worldwide, perpetual, irrevocable, royalty-free basis to BMJ Publishing Group Ltd ("BMJ") its licensees and where the relevant Journal is co-owned by BMJ to the co-owners of the Journal, to publish the Work in this journal and any other BMJ products and to exploit all rights, as set out in our licence.

The Submitting Author accepts and understands that any supply made under these terms is made by BMJ to the Submitting Author unless you are acting as an employee on behalf of your employer or a postgraduate student of an affiliated institution which is paying any applicable article publishing charge ("APC") for Open Access articles. Where the Submitting Author wishes to make the Work available on an Open Access basis (and intends to pay the relevant APC), the terms of reuse of such Open Access shall be governed by a Creative Commons licence – details of these licences and which Creative Commons licence will apply to this Work are set out in our licence referred to above.

Other than as permitted in any relevant BMJ Author's Self Archiving Policies, I confirm this Work has not been accepted for publication elsewhere, is not being considered for publication elsewhere and does not duplicate material already published. I confirm all authors consent to publication of this Work and authorise the granting of this licence.

For peer review only

"Are Pharmacists at the Frontline? A Cross Sectional Analysis of the Practices and Competencies of Community and Hospital Pharmacists Amid the Looming Opioid Crises".

Short title: Opioid-related Competencies among Pharmacists

Word Count:

Abstract: 299

Manuscript: 3868

For peer review only

Naeem Mubarak,[1] Taheer Zahid,[2] Fatima Rahman Rana,[1] Umm-E-Barirah Ijaz,[1] Afshan Shabbir,[1] Mahrukh Manzoor,[1] Nahan Khan,[1] Minahil Arif,[1] Muhammad Mehroz Naeem,[1] Sabba Kanwal,[1] Nasira Saif-ur-Rehman,[3] Che Suraya Zin,[4] Khalid Mehmood,[5] Javaid Asgher,[1,6,7] Mohamed Hassan Elnaem[8*]

[1] Department of Pharmacy Practice, Faculty of Pharmaceutical Sciences, Lahore University of Biological & Applied Sciences, Lahore, Punjab, Pakistan.

[2] Manager, Servaid Pharmacy, 24-M Quaid-e-Azam Industrial Estate, Lahore, Punjab, Pakistan.

[3] Department of Pharmaceutics, Faculty of Pharmaceutical Sciences, Lahore University of Biological & Applied Sciences, Lahore, Punjab, Pakistan.

[4] Kulliyyah of Pharmacy, International Islamic University, Kuantan, Malaysia.

[5] Institute of Information Management, University of the Punjab, Lahore, Punjab, Pakistan.

[6] Lahore Medical & Dental College, Lahore, Punjab, Pakistan.

[7] Doctors Hospital & Medical Centre, Lahore, Punjab, Pakistan.

[8] School of Pharmacy and Pharmaceutical Sciences, Ulster University, Coleraine, United Kingdom.

Corresponding author:
Dr Mohamed Hassan Elnaem,
School of Pharmacy and Pharmaceutical Sciences, Ulster University, Coleraine, United Kingdom.
Email: m.elnaem@ulster.ac.uk
ORCiD: 0000-0003-0873-6541

ABSTRACT

Objectives

Countries are grappling with a rapidly worsening upsurge in the opioid-related overdose deaths, misuse, and abuse. There is a dearth of data in Pakistan regarding the practices and competencies of pharmacists in handling opioid-related issues. Therefore, this study aimed to evaluate the competencies and practices of pharmacists on dispensing and managing opioids.

Design,

Cross-sectional study

Setting

Punjab, Pakistan

Participants

The study deployed a validated survey to evaluate opioid-related competencies and practices of the community and hospital pharmacists.

Results

504 community pharmacists and 279 hospital pharmacists participated in the survey with an overall response rate of 85.5%. Almost half of the respondents "never" or "sometimes" made clinical notes in a journal or dispensing software to monitor ongoing opioid use. Generally, pharmacists were reluctant to collaborate with physicians or notify police regarding the abuse/misuse of opioids. Hospital pharmacists achieved significantly higher Mean Competency Scores than chain and independent community pharmacists ($p<0.05$). In competency evaluation, three priority areas emerged that require additional training i.e., "opioid overdose management", "opioid use monitoring", and "therapeutic uses of opioids".

Conclusion

Both community and hospital pharmacists hold significant positions and potential to contribute meaningfully to the mitigation of harms and risks associated with opioids. Nevertheless, this study underscores notable deficiencies in the competence of pharmacists, whether in hospital or community settings in Punjab, concerning various aspects related to the dispensing and utilization of opioids. It also highlights the pressing need for the development of strategies aimed at improving several practice areas including the documentation, the quality of patient counseling, the effectiveness of reporting mechanisms for opioid abuse, and the stringent enforcement of regulatory policies to curtail opioid misuse. Thus, to combat the looming opioid crisis in Pakistan, it is crucial to address the competency and practices gaps in the pharmacist workforce to offer opioid stewardship activities.

Keywords

Opioid crisis, opioid dispensing, opioid competency, community pharmacist, hospital pharmacists, practices, Pakistan

For peer review only

ARTICLE SUMMARY: STRENGTH & LIMITATIONS

- This study is the first of its kind to systematically assess the practices and competencies of community and hospital pharmacists in relation to opioid use and dispensing, utilizing an extensive framework comprising eleven opioid competencies.
- The study stands out for its inclusivity and generalizability, as the sample of the community and hospital pharmacists represent diversely located nine administrative divisions of the Punjab.
- The length of the survey, with 55 items, could have induced respondent fatigue, potentially affecting the accuracy and completeness of the responses.
- We could not comment on the absence of pharmacists in hospital settings due to the absence of data regarding the total number of pharmacists in a given hospital.
- The study excluded 13 incomplete survey forms, the response rate and the diversity of the sample size achieved still support the generalizability of the results.

For peer review only

For peer review only - http://bmjopen.bmj.com/site/about/guidelines.xhtml

INTRODUCTION

Chronic pain is one of the most debilitating human experiences that affects over a quarter of the global population in terms of significant physical discomfort. [1] Opioid analgesics, also known as narcotics, are the primary drugs for pain relief, which strictly require a prescription of a legally qualified prescriber and notable examples include morphine, oxycodone, fentanyl, codeine, and tramadol. World Health Organization (WHO) reserves opioid analgesics mainly for moderate to severe pain and palliative care. [2,3] Opioids interact with their specific receptors in the brain and elsewhere to produce a powerful pain-numbing effect, a sense of invincibility and euphoria. Nevertheless, their benefits are often offset by the risks associated with their use, such as tolerance, addiction, and overdose-related mortalities especially where opioids were combined with alcohol or anti-anxiety medicine. [4,5] Due to these inherent risks, the dispensing of opioids is subject to stringent regulations and involves meticulous documentation of the patient information, prescriber details, and the exact dose in the narcotic register — a responsibility typically entrusted to a qualified pharmacist. [6,7]

"Opioid crisis" refers to the alarming upsurge in opioid related overdose deaths and addiction, primarily due to escalating opioids use/misuse. According to the world drug report, in 2017, around two-thirds of the total drug-related deaths were attributed to opioids while there have been 68, 849.25 confirmed reports of opioid overdose deaths in 2020. [8] The opioid crisis is not confined to specific regions; it is a trend that extends globally. Asian countries are also grappling with the challenges posed by opioid misuse; thus, the opioid crisis remains a crucial area in global public health research because of its detrimental effect on communities, families, and society, including the socioeconomic burden it imposes on healthcare systems. [9]

Pakistan is the 5th most populous country in the world, with its one province Punjab houses a population of 110 million. [10] Concerns have been mounting about the widespread misuse and abuse of prescription opioids in this colossal population. In Pakistan, inappropriate dispensing, or inept handling of opioids in community or hospital setting is considered a precipitating factor in the opioid crisis. It has been estimated that 4.25 million adults in Pakistan use opioids unsafely and 1.6 million people reportedly misuse prescription opioids, mainly tramadol, codeine, and nalbuphine . [11–13] Pharmacists, whether in community or hospital settings, play a crucial role in controlling the supply and diversion of opioids by carefully managing opioid refills, preventing individuals from purchasing opioids from multiple pharmacies, avoiding the use of irrational opioid combinations with abuse potential, and monitoring the non-medical use of prescription opioids. [14,15] Further, they have a legal obligation to identify opioid misuse and diversion and collaborate with physicians and law enforcement agencies to report, manage, and screen patients with opioid addiction. Thus, pharmacists offer a comprehensive range of services to

For peer review only - http://bmjopen.bmj.com/site/about/guidelines.xhtml

ensure rational opioid use, such as dispensing, counseling, stigma and harm reduction, overdose management, record keeping, recommending replacement therapies, and safe disposal guidance. [6,16] However, efficiently delivering the above-mentioned services requires essential opioid dispensing competencies of the handling pharmacist. Opioid dispensing competencies refer to a pharmacist's knowledge, and skills to promote rational opioid use and reduce non-medical or misuse in society. [17] A recent study gained expert consensus and identified 11 key areas (competencies) for pharmacists on opioid dispensing, based on competency framework devised by the Association of Faculties of Pharmacy of Canada's Opioid Working Group. [17,18] These competencies serve as a foundation for evaluating pharmacists' professional practice standards to promote rational opioid use in each society.

Despite the severity of the opioid crisis, there has been a dearth of studies evaluating the competence of pharmacists in Pakistan. It is important for pharmacists to ensure they are up to date with opioid dispensing guidelines. Assessing their competency in this area will provide evidence of their readiness to deal with the looming opioid crisis and identify areas where additional training is needed, to improve their capabilities to serve global health objectives. Therefore, this study aimed to assess and compare community and hospital pharmacists' practices and competencies in dispensing, using, and managing opioids in Punjab, Pakistan.

METHODS

Study Design, Setting & Sample

It was a cross-sectional study that collected data from the community and hospital pharmacists in all nine administrative divisions of the province of Punjab in addition to Islamabad Capital Territory.

According to official sources, there were 23,029 registered pharmacists in Punjab. [19] To obtain the sampling frame for community pharmacies, we formally requested a division-wise list of all licensed community pharmacies in Punjab from the Provincial Drug Control Unit, which provided the required information. The sampling frame encompassed 10,417 community pharmacies. Similarly, the sampling frame for hospital pharmacists was obtained from the primary and secondary health official website. In Punjab, there were 664 hospital pharmacists in public sector hospitals, while when combined with pharmacists in private hospitals (approximately one-fourth of the population in public hospitals), the sampling frame reached around 830 hospital pharmacists. [20] We used "Raosoft," an online sample size calculator, to compute an appropriate sample size for community and hospital pharmacists. Sample size calculations were based on a 5% margin of error, 95% confidence interval, a response distribution of 50%, and a maximum population of 10,417 (for community

For peer review only - http://bmjopen.bmj.com/site/about/guidelines.xhtml

pharmacists) and 830 (for hospital pharmacists). The calculator determined a target sample size (n = 371) for community pharmacists and hospital pharmacists (n = 263) to represent their respective populations.

Consequently, to achieve the target sample size, we aimed at a higher number of community and hospital pharmacies, considering potential refusals and dropouts. Further, we used a stratified random sampling technique to reduce the sampling bias and obtain a diverse, representative sample of community and hospital pharmacists.

Survey Instrument

The survey instrument utilized the concept of 11 core opioid dispensing competencies, and survey items were informed by an extensive review of the relevant literature. [13,17,18,21–25]

The survey instrument comprised three main sections:

1- Demographic

2- Routine practices regarding opioid use and dispensing.

3- Core opioid competencies

The third section was further divided into 11 sub-sections corresponding to 11 core opioid competencies. These competencies include understanding societal, physiological, and pharmacological aspects of opioids, recognizing opioid therapeutic applications, and managing opioid overdoses. Knowledge of pain management and adherence to state laws related to opioids was also included. [17,18] Competencies include skills like counselling, communication, and understanding addiction issues, detecting vulnerable groups for signs of opioid addiction, overdose, or misuse is important. Each competency was evaluated using five statements (survey items) which required the respondent to choose the correct answer out of five exclusive options. Total scores were computed using a summative scale for comparisons based on gender, pharmacy practice type, location, and professional development course completion. Please refer to S1 Appendix for the full-length survey. 'See electronic supplementary material S1 Appendix'.

Validity and Reliability of the Survey Instrument

After an extensive literature review to develop survey items, face, and content validity evaluations were conducted by two faculty members, two community pharmacists, and one hospital pharmacist. Moreover, to ensure the internal consistency/ reliability of the survey items, a pilot study involving 50 pharmacists (33 community pharmacists and 17 hospital pharmacists) was conducted, and Cronbach's alpha statistics were applied. Results of

For peer review only

For peer review only - http://bmjopen.bmj.com/site/about/guidelines.xhtml

the reliability analysis showed that the items in the 11 competencies had a satisfactory level (0.7-0.9) of internal consistency ('See electronic supplementary material S1 Table').

Definitions, Inclusion, and Exclusion Criteria

In context of this study, a pharmacy within the premises of a hospital was referred to as a hospital pharmacy, which can further be categorized based on the type of hospital they are associated with, such as public or private hospitals. On the other hand, any pharmacy outlet outside the premises of a hospital was defined as a community pharmacy. While the existing scholarly work lacks a consensus-based definition of a chain or an independent pharmacy. Chain pharmacies are typically a corporation with 6-200+ pharmacies operating under a nationwide banner. On the other hand, an independent pharmacy follows a small operator model with an individual owning up to five pharmacies. Thus, for our research, we defined a pharmacy as a chain if more than five pharmacies were operating under the same name and the business used distinctive branding across all pharmacies. Detailed inclusion/exclusion criteria are given in Table 1.

Table 1 Inclusion-Exclusion criteria

Inclusion	Exclusion
Any community or hospital pharmacy registered under a category-A license, located in any of the nine divisions of Punjab or Islamabad Capital Territory was eligible to be included in the study.	Pharmacies without a category-A license or medical stores with category-B license, or designated dispensaries were excluded from data collection.
Pharmacists registered in category-A (legally permitted to handle opioids) were included.	Assistant pharmacist or pharmacy technicians or pharmacy interns (who had not completed the Pharm-D degree) were excluded.
Secondary or tertiary care hospitals both in public and private sector were included	Primary care clinics/facilities were excluded because generally the facility is smaller and do not have a pharmacist within the premises especially in the private sector.
Survey forms completed in all respects were included in the final count	Incomplete/missing survey forms were excluded.

Data Collection and Reporting

The first author conducted a comprehensive training of a team of Pharm-D students and research associates on different aspects of data collection. The team was divided into subgroups assigned to different divisions of Punjab. A second visit was made if the pharmacist was unavailable, or the pharmacy was occupied in rush hours during the first visit. The survey was administered in a face-to-face meeting. Data collection began on December 10, 2022, and ended on January 15, 2023. The data were reported in accordance with STROBE guidelines ('See electronic supplementary material S2 Appendix').

For peer review only - http://bmjopen.bmj.com/site/about/guidelines.xhtml

Data Management and Statistical Analysis

We used Statistical Package Social Sciences (version 22 IBM, California, USA) for data management and statistical analysis. Descriptive statistics were used to evaluate categorical variables, while differences in mean scores were compared between two groups for different variables using a t-test. To conduct quantitative analysis, we made groups based on different variables, such as gender, nature of the employment, and type of pharmacy practice. To determine the mean difference among three or more groups, we conducted One-way ANOVA followed by post-hoc analysis. The significance level for detecting differences in means was set at a p-value of ≤ 0.05.

Ethical Approval

Research Ethics Committee, Lahore Pharmacy College, Lahore Medical & Dental College granted ethical approval for this study (ref# LPC/ETH/25/09/22, 'See electronic supplementary material S3 Appendix'). Before data collection, the research team explained the study objectives to potential participants and obtained their voluntary consent (informed consent). The research team assured anonymity of the response. Finally, neither the authors nor the data collectors had access to identifying the survey respondents at any research stage.

RESULTS

Data were collected from community and hospital pharmacies diversely located in all administrative divisions of the province of Punjab, namely Lahore (provincial capital), Sargodha, Multan, Faisalabad, Bahawalpur, Rawalpindi, Sahiwal, and Dera Ghazi Khan besides Islamabad Capital Territory. During the first visit, pharmacists were present in only 44.8 % of the pharmacies visited. This presence of pharmacists was further declined when the research team moved from the provincial capital to other cities, and the lowest presence (18.9%) was recorded in Faisalabad. However, the presence increased significantly on the second visit. A total of (n=783) pharmacists completed the survey (community pharmacist= 504; hospital pharmacist = 279). Most respondents were full-time employed males within the age range of 26-30. A mere 22.6% of the pharmacists reported involvement in any continuous professional development program within the past two years. Table 2 provides a summary of the demographics of the survey participants.

For peer review only

Table 2 Demographics of the survey participants

<table>
<tr><th>Variable</th><th colspan="2">Category</th><th colspan="2">n (%)</th></tr>
<tr><td rowspan="2">Gender</td><td colspan="2">Male</td><td colspan="2">503 (64.2)</td></tr>
<tr><td colspan="2">Female</td><td colspan="2">280 (35.8)</td></tr>
<tr><td rowspan="8">Age group</td><td colspan="2">23-25</td><td colspan="2">190 (24.3)</td></tr>
<tr><td colspan="2">26-30</td><td colspan="2">367 (46.9)</td></tr>
<tr><td colspan="2">31-35</td><td colspan="2">139 (17.8)</td></tr>
<tr><td colspan="2">36-40</td><td colspan="2">57 (7.3)</td></tr>
<tr><td colspan="2">41-45</td><td colspan="2">19 (2.4)</td></tr>
<tr><td colspan="2">46-50</td><td colspan="2">9 (1.1)</td></tr>
<tr><td colspan="2">56-60</td><td colspan="2">2 (0.3)</td></tr>
<tr><td colspan="2">Total</td><td colspan="2">783 (100)</td></tr>
<tr><td rowspan="2">You studied/completed Pharm-D/B-Pharm degree in a ________ institute.</td><td colspan="2">Public</td><td colspan="2">403 (51.5)</td></tr>
<tr><td colspan="2">Private</td><td colspan="2">380 (48.5)</td></tr>
<tr><td rowspan="2">Mode of assessment in your Pharm-D/B-Pharm was:</td><td colspan="2">Annual</td><td colspan="2">366 (46.7)</td></tr>
<tr><td colspan="2">Semester</td><td colspan="2">417 (53.3)</td></tr>
<tr><td rowspan="2">Your highest level of education</td><td colspan="2">Masters</td><td colspan="2">155 (19.8)</td></tr>
<tr><td colspan="2">Pharm-D/B-Pharm</td><td colspan="2">628 (80.2)</td></tr>
<tr><td rowspan="2">Have you completed any continuous professional development course in the last TWO years?</td><td colspan="2">Yes</td><td colspan="2">177 (22.6)</td></tr>
<tr><td colspan="2">No</td><td colspan="2">606 (77.4)</td></tr>
<tr><td rowspan="2">Employment status</td><td colspan="2">Full-time</td><td colspan="2">614 (78.4)</td></tr>
<tr><td colspan="2">Part-time</td><td colspan="2">169 (21.6)</td></tr>
<tr><td rowspan="4">Type of pharmacy practice</td><td rowspan="2">Community</td><td>Chain</td><td>291 (37.2)</td><td rowspan="2">504 (64.4)</td></tr>
<tr><td>Independent</td><td>213 (27.2)</td></tr>
<tr><td rowspan="2">Hospital</td><td>Public</td><td>187 (67)</td><td rowspan="2">279 (35.6)</td></tr>
<tr><td>Private</td><td>92 (33)</td></tr>
</table>

n= number

The overall response rate collectively for community and hospital pharmacists was 85.5%, as shown in S2 Table (‘See electronic supplementary material S2 Table’). However, it is essential to note that many pharmacists (mostly in hospitals) declined to participate in the survey due to reasons, such as a busy schedule, lack of motivation, or fear of potential regulatory consequences.

For peer review only - http://bmjopen.bmj.com/site/about/guidelines.xhtml

The survey also examined the trends of professional practices on opioid use and dispensing among community and hospital pharmacists. Half of the respondents mentioned that they "never" or "sometimes" made clinical notes in a journal or dispensing software to monitor ongoing opioid use. Roughly one-third of the respondents reported that they "almost always" counsel patients on the possible side effects and overdose risks associated with opioid use. Similarly, only 18% reported that they "almost always" contacted police or physicians regarding the abuse of opioids or clarify an opioid prescription. Table 3 presents the trends of practice among pharmacists.

Table 3 Practices among pharmacists on opioid use

Statements	Never n (%)	Rarely n (%)	Sometimes n (%)	Very Often n (%)	Almost Always n (%)
**I______ make clinical notes in a journal/dispensing software (apart from the narcotic register) to monitor ongoing opioids use.**	179 (22.9)	166 (21.2)	178 (22.7)	121 (15.5)	139 (17.8)
**I ______ counsel patients on the possible side effects of opioids.**	26 (3.3)	89 (11.4)	187 (23.9)	217 (27.7)	264 (33.7)
**I ______ counsel patients on opioids overdose risk.**	40 (5.1)	86 (11.0)	184 (23.5)	189 (24.1)	284 (36.3)
**I ______ refuse the supply of the opioids when in doubt of abuse.**	36 (4.6)	75 (9.6)	105 (13.4)	161 (20.6)	406 (51.9)
**I ______ notify police/regulatory bodies on the abuse of opioids.**	203 (25.9)	154 (19.7)	145 (18.5)	135 (17.2)	146 (18.6)
**To clarify the opioids prescription, I ______ call the physician.**	76 (9.7)	140 (17.9)	294 (37.5)	129 (16.5)	144 (18.4)

n= number

Statistical analysis revealed no significant difference in the overall mean competency scores against the variables, such as gender, community vs. hospital settings, or system of assessment used in the Pharm-D degree program or type of hospital (private or public). In contrast, full-time employed pharmacists with a higher degree after Pharm-D, as well as those who had completed continuous professional development courses, achieved significantly higher mean competency scores. Similarly, pharmacists with a degree from a public university scored slightly higher (significance at borderline) than pharmacists who received a Pharm-D degree from a private educational institute. Table 4 summarizes the overall mean competency score against each variable.

For peer review only - http://bmjopen.bmj.com/site/about/guidelines.xhtml

Table 4 Different variables against the summative mean score of all the core opioids competencies

Variable	Category	Mean (SD)	t* (p-value)
**Gender**	Male	19.64 (8.301)	-0.816 (0.415)
	Female	20.14 (8.019)	
**System of assessment in your Pharm-D degree program**	Annual	20.25 (8.455)	1.393 (0.164)
	Semester	19.44 (7.959)	
**Type of institute**	Public	20.36 (8.700)	1.921 (0.055) *
	Private	19.24 (7.602)	
**Highest level of education**	Masters	21.99 (9.099)	**3.408 (0.001)** *
	Pharm-D/B-Pharm	19.28 (7.878)	
**Employment status**	Full-time	20.50 (8.180)	**4.519 (0.000)** *
	Part-time	17.33 (7.800)	
**Have you completed any continuous professional development course in the last TWO years?**	Yes	20.86 (9.101)	**2.079 (0.038)** *
	No	19.41 (7.787)	
**Type of pharmacy practice**	Community	19.40 (7.869)	-1.846 (0.065)
	Hospital	20.57 (8.730)	
**If pharmacy is in hospital, type of hospital:**	Public	21.05 (9.047)	1.316 (0.189)
	Private	19.59 (8.004)	

***Independent t-test, p-value (≥ 0.05) Bold values indicate statistical significance.**

SD= standard deviation

On the other hand, results of ANOVA revealed that statistically significant differences (p=0.006) exist among the three pharmacist groups compared, i.e., hospital, chain, and independent pharmacist. Hospital pharmacists secured higher mean competency scores than all other sub-groups (both chain and independent). Further, the mean competency scores for pharmacists at chain pharmacies were better than pharmacists at independent pharmacies, as shown in Table 5.

For peer review only - http://bmjopen.bmj.com/site/about/guidelines.xhtml

Table 5 Comparison of different types of pharmacy practice

Type of Pharmacy Practice	Groups	Mean Difference	p-value
**Chain**	Independent	1.904	**.010 ***
	Hospital	-0.357	.602
**Independent**	Chain	-1.904	**.010 ***
	Hospital	-2.261	**.002***
**Hospital**	Chain	0.357	.602
	Independent	2.261	**.002 ***

***p-value (≤ 0.05)**

S3 Table details the mean score for each of the eleven individual opioid competencies against different variables ('See electronic supplementary material S3 Table'). Comparatively, among the eleven opioid competencies, community pharmacists received the lowest mean competency score in "therapeutic use of opioids" (C-5= 1.34), "opioid and society" (C-1 = 1.39), and "opioid use monitoring" (C-10= 1.41) thus emerged as a priority area to focus for further training. For hospital pharmacists, "opioid use monitoring" (C-10= 1.45), "therapeutic use of opioids" (C-5= 1.52), "opioid and society" (C-1 = 1.56), and "opioid overdose management" (C-11 = 1.56) had the lowest mean scores.

DISCUSSION

The findings of this study portray a bleak picture of the current professional practices and competencies of pharmacist workforce related to opioid use and dispensing in Punjab, Pakistan.

First, it is pertinent to mention that pharmacist availability, especially in the community setting, was challenging during the first visit to pharmacies. Our study confirms the earlier findings of Bashir and colleagues, which reported absence of pharmacists in 50% of the pharmacies in different regions of Punjab. [26] Pharmacies without a qualified pharmacist or operating on a proxy could precipitate an upsurge in the dispensing of opioids without a prescription and pervasive misuse and abuse of prescription opioids. Further, this finding implies poor regulatory check and balance and raises serious concerns on the consequences of low engagement of community pharmacists in opioid management. Thus, pharmacists, especially in community settings are not visible at the frontline. The government should ensure the implementation of the mandatory presence of a qualified person

For peer review only - http://bmjopen.bmj.com/site/about/guidelines.xhtml

(pharmacist) in community pharmacies in Punjab. The loopholes in the current regulatory policies and the partial implementation of the Control of Narcotic Substances Act 1967 are also attributed to opioid misuse and unauthorized dispensing. A few countries, including India, Ukraine, and Colombia, have recently amended their laws to engage community pharmacists to offer opioid stewardship activities, though the implementation is slow. [9,27]

Second, findings of this study reveal a potential gap in the documentation practices, which are crucial for ensuring patient safety and continuity of care. Nevertheless, studies support the exigent need for community pharmacist engagement to facilitate the implementation of safe opioid use in society. [6,15] This situation is further exacerbated by the low engagement of pharmacists in professional development programs, which emphasizes the need for initiatives to make continuous education and training programs a regulatory prerequisite for practice. To handle this, recent research endorsed that prior training results in better competency of community pharmacists in handling medication opioid use disorder. [12] Thus, community and hospital pharmacists must undergo continuous professional education to dispense opioids and follow standard guidelines.

Third, unfortunately, only one-third of the pharmacists reported that they "almost always" counseled patients on the possible side effects and overdose risks associated with opioid use. While pharmacists have the professional scope to educate patients about opioid-related risks and benefits, suggest alternatives, conduct medication reviews as part of an interdisciplinary team, and counsel on opioid substitution therapy and management of opioid poisoning. [6,28] These findings may be correlated with the recent surge in the abuse of opioid analgesics, particularly Nalbuphine, in Pakistan. [12] Another study in Pakistan also attributed unchecked dispensing at pharmacies with much of the opioid's abuse and misuse in Pakistan. Thus, to curtail the opioid related overdose mortalities, enhanced patient education especially in the community setting could improve the opioid use in the society.

Fourth, pharmacists were reluctant to call the physician to clarify opioid-related prescriptions, indicating a lack of interprofessional collaboration between community pharmacists and general practitioners. These findings indicate the importance and urgent need to enhance interprofessional communication and collaboration, especially in community settings. An Australian study identified that authentication of the prescription by contacting the prescriber as one of the crucial strategies used in prescription opioid monitoring. [14] Ensuring appropriate opioid use requires devising a mechanism or channel of communication between the prescriber and pharmacist, especially in a community setting in Pakistan. Numerous studies provide evidence for the positive outcomes of collaborative care on medication reviews, identifying prescribing errors, and intervening in opioid

For peer review only

For peer review only - http://bmjopen.bmj.com/site/about/guidelines.xhtml

misuse in other countries. [29,30] Similarly, only a handful of respondents reported that they notified the police or regulatory bodies regarding the abuse of opioids, highlighting a potential lack of awareness or reluctance to report such cases. The collaboration of pharmacists with law enforcement agencies, addiction consultants, policymakers, regulators, epidemiologists, and pharmaceutical companies is equally important.

Fifth, higher competency scores of hospital pharmacists compared with chain and independent pharmacies may be linked to their higher exposure and clinical and patient care experience in hospital settings. Similarly, better competency scores at chain pharmacies compared to their counterparts in independent pharmacies could be attributed to the fact that chain pharmacies hold more financial resources, which they spent on the training and development of their pharmacists.

Finally, the pharmacist who had studied under the current Pharm-D curriculum in Pakistan lack sufficient competencies to practice across all scopes of opioid management and dispensing. The lowest overall mean competency scores in "opioid overdose management", "opioid use monitoring", and "therapeutic use of opioids" indicate the top three priority areas which require further training and education. These findings align with the conclusion drawn in a recent qualitative study on the dearth of skill-based competency among pharmacists, such as identifying and communicating prescription errors, therapeutic duplication, monitoring unauthorized dispensing, and providing patient-centered care. [12] A Canadian study also validated similar key areas of training in opioid and pain management, alternative pain management, naloxone dispensing, collaborating with physicians, and counseling patients on opioid use and misuse. [31] Another possible reason for these poor competency scores may be related to the limited coverage of these topics in the Pharm-D curriculum in Pakistan. Further, the Pharm-D curriculum in Pakistan has not been revised for a decade, making it difficult for fresh graduates to adapt to the updated guidelines on pain management and opioid use. [32] This highlights the urgent need to upgrade the Pharm-D curriculum and incorporate opioid learning modules with updated guidelines. Lessons should be learned from the case of the US, where, in line with the recent opioid crisis, pharmacy schools are incorporating specific opioids related competencies into their Doctor of Pharmacy (Pharm-D) curriculums to ensure that students are well-prepared to handle the complex issues surrounding opioid use and misuse. [33,34]

Implications for Policy, Practice and Future Research

Based on the findings of this study, the authors suggest the following implications for policy, practice, and further research:

For peer review only - http://bmjopen.bmj.com/site/about/guidelines.xhtml

1. It is crucial to address the gaps in knowledge and practice related to therapeutic use of opioids, opioid use monitoring, and opioid overdose management. Improving pharmacist competency in these areas could contribute to the safe and effective use of opioids, thereby reducing the risks associated with their misuse and abuse.
2. Strategies should be developed to promote the documentation of clinical notes and enhanced patient counselling on opioid risks.
3. The notification of opioid misuse to regulatory authorities and clarification of opioid-related prescription from the physicians should be made a binding requirement.
4. To curb opioid misuse, the availability of a qualified personnel at community pharmacist should be ensured.
5. Community pharmacists must pass a licensing exam that assess the different opioid dispensing competencies of pharmacists before recruitment in community and hospital settings.
6. An electronic national opioid database system should be developed to store the records of all opioid prescriptions. This will assist in tracing the unauthorized prescribing and dispensing of opioids.
7. The Pharm-D curriculum should be revised, and opioids must be covered in depth at the graduate and undergraduate level primarily related to addiction, misuse, and diversion.
8. Future research should prioritize the examining of policies and interventions aimed at ensuring the active participation of pharmacists in community pharmacies. Furthermore, there is a need for research to focus on the development of evidence-based opioid stewardship interventions, clinical support tools and collaborative practice models fostering enhanced communication among the pharmacist, prescribers, and regulatory bodies.

For peer review only

Strength & Limitations

The main strengths of this work are related to being the pioneer study to report the practices and competencies of community and hospital pharmacists on opioid use and dispensing in a systematic way i.e., under the framework of eleven opioid competencies. It thus may be taken as a call for action. Our study did not select a convenient sample of the community pharmacies and hospital pharmacists, instead, it reached out to all nine diverse divisions of Punjab to present data representative of the population.

The current study should be interpreted in the light of its limitations. It was a long survey consisting of 55 items that may have resulted in the fatigue bias of the respondent. However, it was inevitable in the context of the coverage of essential 11 core competencies related to opioid use and dispensing. In the case of community pharmacy, the presence percentage was possible because one pharmacist/pharmacy generally follows the law of the land. However, we could not comment on the absence of pharmacists in hospital settings due to the absence of data regarding the total number of pharmacists in a hospital. We did not include 13 incomplete survey forms, however, given the response rate, diverse and vast sample size achieved, the findings remain the representative of the population and results are expected to be generalizable.

CONCLUSION

While the opioid crisis continues to inflict grave and unsettling consequences, both community and hospital pharmacists hold significant positions and potential to contribute meaningfully to the mitigation of harms and risks associated with opioids at both individual and societal levels. Nevertheless, this study underscores notable deficiencies in the competence and practices of pharmacists, whether in hospital or community settings in Punjab, concerning various aspects related to the dispensing and utilization of opioids. It also highlights the pressing need for the development of strategies aimed at improving several practice areas including the documentation, the quality of patient counseling, the effectiveness of reporting mechanisms for opioid abuse, and the stringent enforcement of regulatory policies to curtail opioid misuse. Thus, to combat the looming opioid crisis in Pakistan, it is crucial to equip pharmacists with the necessary competencies to offer opioid stewardship activities to ensure responsible opioid use. However, achieving this goal necessitates a forward-thinking approach, an eagerness to collaborate and notify physicians and regulatory authorities, a willingness to honestly introspect and acknowledge the lack of competencies or past failed efforts, and an investment in the training of pharmacy human resources. Embracing this life-saving commitment is imperative for the well-being of the patients, communities, and the pharmacy profession.

For peer review only - http://bmjopen.bmj.com/site/about/guidelines.xhtml

DECLARATIONS

Competing Interest

The authors have no relevant financial or non-financial interests to disclose.

Data Sharing Statement

All relevant data, materials, and information necessary to understand, replicate, and build upon the findings presented in this article have been made available within the main manuscript and supplementary files associated with this publication.

Acknowledgement

We extend our heartfelt thanks to the Provisional Drug Control Unit, Punjab, for providing the list of community pharmacies across Punjab.

Author Contribution

Naeem Mubarak: Conceptualization, Methodology, Formal analysis, Writing - original draft preparation, Writing - review and editing, Resources, Supervision
Taheer Zahid: Methodology, Iinvestigation, Resources, Writing - review and editing
Fatima Rahman Rana: Methodology, Investigation, Formal analysis, Writing - original draft preparation, Writing - review and editing, Resources.
Umm-E-Barirah Ijaz: Methodology, Iinvestigation, Resources, Writing - review and editing
Afshan Shabbir: Methodology, Iinvestigation, Resources, Writing - review and editing
Mahrukh Manzoor: Methodology, Investigation, Resources
Nahan Khan: Methodology, Iinvestigation, Resources, Writing - review and editing
Minahil Arif: Methodology, Iinvestigation, Resources, Writing - review and editing
Muhammad Mehroz Naeem: Methodology, Iinvestigation, Resources, Writing - review and editing
Sabba Kanwal: Methodology, Formal analysis, Resources, Writing - review and editing
Nasira Saif-ur-Rehman: Methodology, Iinvestigation, Resources, Supervision, Writing - review and editing
Che Suraya Zin: Methodology, Investigation, Writing - review and editing, Resources, Supervision
Khalid Mehmood: Methodology, Formal analysis, Writing - review and editing, Resources, Supervision
Javaid Asgher: Methodology, Investigation, Writing - review and editing, Resources, Supervision
Mohamed Hassan Elnaem: Methodology, Formal analysis, Writing - review and editing, Resources, Supervision

Prior Publication

This paper has not been submitted elsewhere in similar form, and all authors have contributed significantly to the publication. All authors are aware of the submission and agree with it.

For peer review only

For peer review only - http://bmjopen.bmj.com/site/about/guidelines.xhtml

Funding

The authors declare that no funds, grants, or other support were received during the preparation of this manuscript.

SUPPORTING INFORMATION

S1 Table = Highlights Cronbach's alpha values against individual opioid competencies

S2 Table= Response Rate for Community and Hospital Pharmacists in the nine divisions of Punjab

S3 Table = Details the individual competency scores against different variables

S1 Appendix= Outlines the final version of the survey administered

S2 Appendix= Provides STROBE guidelines

S3 Appendix= Depicts Ethical approval document

For peer review only

REFERENCES

1. Macchia L, Oswald AJ. Social Science & Medicine Physical pain , gender , and the state of the economy in 146 nations. Soc Sci Med [Internet]. 2021;287(December 2020):114332. Available from: https://doi.org/10.1016/j.socscimed.2021.114332
2. Amaechi O, Human MM, Featherstone K. Pharmacologic Therapy for Acute Pain. Vol. 104, American Family Physician. 2022.
3. Anekar AA, Hendrix JM, Cascella M. WHO Analgesic Ladder. Stat Pearls Publishing. 2023. p. 1–7.
4. Rauf U, Ali M, Dehele I, Paudyal V, et al. Causes, nature and toxicology of fentanyl-analogues associated fatalities: A systematic review of case reports and case series. J Pain Res. 2021;14:2601–14.
5. Alrashdi S, Paudyal V, Elnaem MH, et al. Causes, Nature and Toxicology of Tramadol-Associated Deaths Reported in the Peer-Reviewed Literature: A Systematic Review of Case Studies and Case Series. Drugs Ther Perspect [Internet]. 2021;37(9):422–32. Available from: https://doi.org/10.1007/s40267-021-00859-6
6. Gregory T, Gregory L. The Role of Pharmacists in Safe Opioid Dispensing. J Pharm Pract. 2020;33(6):856–62.
7. Jantarada C, Silva C, Guimarães-Pereira L. Prevalence of Problematic Use of Opioids in Patients with Chronic Noncancer Pain: A Systematic Review with Meta-analysis. Pain Pract. 2021;21(6):715–29.
8. UNODC, World Drug Report 2022 (United Nations publications, 2022)
9. Zachary, Z., Kathryn, F., Hanna, GP., Anna Z. A global study of pain prevalence across 52 countries : examining the role of country- level contextual factors : PAIN. Pain. 2022;163(9):1–12.
10. Mubarak N. Corona and clergy – The missing link for effective social distancing in Pakistan: Time for some unpopular decisions. Int J Infect Dis. 2020;95(January):431–2.
11. Iqbal MJ, Ali M, Rizwan, Ghafoor, et al. Study the different incidences of Tramadol in different cities of Punjab. Vol. 11. 2021.
12. Iqbal A, Knaggs R, Anderson C, et al. Logic model for opioid safety in chronic non ‑ malignant pain management , an in ‑ depth qualitative study. Int J Clin Pharm [Internet]. 2022;45(1):220–32. Available from: https://doi.org/10.1007/s11096-022-01493-6
13. Islam M. State of the art treatment options for Pakistan's opioid, alcohol and methamphetamine crisis. J Pak Med Assoc. 2020;70(6):1063–8.
14. Alvin M, Picco L, Wood P, et al. Community pharmacists ' preparedness to intervene with concerns around prescription opioids : findings from a nationally representative survey. Int J Clin Pharm [Internet]. 2020;43(0123456789):411–9. Available from: https://doi.org/10.1007/s11096-020-01152-8
15. Webb K, Cernasev A, Li MS, et al. An Exploratory Study of Pharmacist Perceptions of Opioid Interventions for Acute Pain. J Pharm Technol. 2021;37(1):36–44.
16. Underwood N, Cremer L, Cance JD, et al. The impact of community-level prevention strategies on high-dose opioid dispensing rates: 2014-2019. Drug Alcohol Depend. 2021 Oct;227:108988.
17. Maher E, Nielsen S, Summers R, et al. Core competencies for Australian pharmacists when supplying prescribed opioids: a modified Delphi study. Int J Clin Pharm. 2021 Apr;43(2):430–8.
18. Sproule B. AFPC Commitment to the Joint Statement of Action to Address the Opioid Crisis in Canada. 2018.
19. Punjab Pharmacy Council (2023) https://punjabpharmacycouncil.com/index_files/Page528.htm-(Accessed on-1-7-2023)
20. Punjab, G. of. (2023). Primary & Secondary Health Care Department (pp. 1–71). https://pshealthpunjab.gov.pk/-(Accessed on 1-7-2023)
21. Rosen DM, Alcock MM, Palmer GM. Opioids for acute pain management in children. Anaesth Intensive Care. 2022;50(1–2):81–94.
22. Pattullo GG. Opioids in acute pain: Towards getting the right balance. Anaesth Intensive Care. 2022;50(1–2):68–80.
23. E.Hall J. Guyton and Hall: Textbook of Medical Physiology. 12th editi. William Schmitt; Rebecca Gruliow; Frank Morales, editor. Saunders. Saunders; 2013. 1112 p.
24. Laurence L. Brunton; Randa Hilal-Dandan; Björn C. Knollmann. Goodman & Gilman's: The Pharmacological Basis of Therapeutics [Internet]. 13th editi. Randa Hilal-Dandan; Bjorn Knollmann, editor. Mc Graw Hill Education; 2022. 1423 p. Available from: https://accesspharmacy.mhmedical.com/book.aspx?bookID=2189
25. Brady JE, Wunsch H, DiMaggio C, et al. Prescription drug monitoring and dispensing of prescription opioids. Public Health Rep. 2014;129(2):139–47.
26. Bashir I, Ahmad M, Jamshaid M, et al. Heliyon Illicit sale of controlled drugs at community pharmacy / medical stores of Punjab , Pakistan : A road to demolition of public health. Heliyon [Internet]. 2021;7(November 2020):e07031. Available from: https://doi.org/10.1016/j.heliyon.2021.e07031

For peer review only

For peer review only - http://bmjopen.bmj.com/site/about/guidelines.xhtml

27. Volkow ND, McLellan AT. Opioid Abuse in Chronic Pain — Misconceptions and Mitigation Strategies. N Engl J Med. 2016;374(13):1253–63.
28. Aziz MM, Ji W, Masood I, et al. Patient satisfaction with community pharmacies services: A cross-sectional survey from Punjab; Pakistan. Int J Environ Res Public Health. 2018;15(12).
29. Mubarak N, Hatah E, Aris MAM, et al. Consensus among healthcare stakeholders on a collaborative medication therapy management model for chronic diseases in Malaysia; A Delphi study. PLoS One. 2019;14(5):e0216563–e0216563.
30. Moecker R, Weissenborn M, Klingenberg A, et al. Task sharing in an interprofessional medication management program – a survey of general practitioners and community pharmacists. BMC Health Serv Res. 2022;22:1–10.
31. Balough MM, Nwankpa S, Unni EJ. Readiness of Pharmacists Based in Utah About Pain Management and Opioid Dispensing. Pharm (Basel, Switzerland). 2019 Jan;7(1).
32. Mubarak N, Arif S, Irshad M, et al. How are we educating future physicians and pharmacists in pakistan? A survey of the medical and pharmacy student's perception on learning and preparedness to assume future roles in antibiotic use and resistance. Antibiotics. 2021;10(10).
33. Hohmeier KC, Cernasev A, Sensmeier M, et al. U.S. student pharmacist perceptions of the pharmacist's role in methadone for opioid use disorder: A qualitative study. SAGE Open Med. 2021;9:205031212110229.
34. Renfro ML, Moczygemba LR, Baumgartner J, et al. Opioid-Related Education Provided by Continuing Education Divisions at US Pharmacy Schools. Am J Pharm Educ. 2020 Oct;84(10):ajpe8001.

For peer review only

For peer review only - http://bmjopen.bmj.com/site/about/guidelines.xhtml

S1 Table Cronbach's alpha value against each of the eleven opioid competencies

Sr.	Competency	Alpha*
**1.**	Opioids & Society	0.654
**2.**	Opioids' Physiology	0.800
**3.**	Opioids' Pharmacology	0.776
**4.**	Opioids' Pharmacokinetic & Drug Interaction	0.826
**5.**	Therapeutic use of Opioids	0.746
**6.**	Opioids' Education	0.740
**7.**	Opioids Dispensing	0.833
**8.**	Opioids & Pain Management	0.852
**9.**	Course of Opioid Therapy	0.844
**10.**	Opioids' Monitoring	0.871
**11.**	Opioid Overdose Management	0.852

*** Cronbach's alpha coefficient test**

For peer review only

S2 Table Response Rate for Community and Hospital Pharmacists in the nine divisions of Punjab

Sr.	Division in Punjab	Total No. of Community Pharmacies Visited	No. of CPs Present on the 1st visit	Total No. of CPs Administered the Survey by the 2nd visit	No. of CPs Refused	Total No. of CPs Completed the Survey	*Response Rate of the CPs*	Total No. of HPs Administered the Survey	No. of HPs Refused	No. of HPs Completed the Survey	*Response Rate of the HPs*
		n	n (%)	(a)	n	(b)	*(%)*	(c)	n	(d)	*(%)*
**1**	**Lahore**	249	146 (58.6)	236	9	227	*96.2*	155	27	128	*82.5*
**2**	**Bahawalpur**	66	30 (45.5)	48	4	44	*91.7*	10	5	5	*50*
**3**	**Rawalpindi/ Islamabad**	41	22 (53.7)	32	3	29	*90.6*	15	4	11	*73.3*
**4**	**Faisalabad**	79	15 (18.9)	31	6	25	*80.6*	18	12	6	*33.3*
**5**	**Gujranwala**	181	70 (38.7)	103	7	96	*93.2*	69	14	55	*79.7*
**6**	**Multan**	57	24 (42.1)	39	5	34	*87.2*	39	9	30	*76.9*
**7**	**Sargodha**	43	18 (41.9)	25	3	22	*88*	33	8	25	*75.7*
**8**	**Sahiwal**	34	13 (38.2)	23	2	21	*91.3*	18	7	11	*61.1*
**9**	**Dera Ghazi Khan**	14	4 (28.6)	8	2	6	*75*	14	6	8	*57.1*
***Overall*/ Total**		**764**	**342 (44.8)**	**545**	**41**	**504**	***94.98***	**371**	**92**	**279**	***75.2***

***Overall pharmacist response rate** = $\frac{b + d}{a + c} \times 100 = \frac{783}{916} \times 100 = 85.5\%$

HP= Hospital Pharmacist, **CP= Community Pharmacist.

***Each division consists of various districts, for instance:

Lahore= Lahore, Sheikhupura, Kasur, Nankana Sahib; **Bahawalpur**= Bahawalpur, Bahawalnagar, Rahim Yar Khan; **Rawalpindi/Islamabad**= Rawalpindi, Jhelum, Chakwal, Attock; **Faisalabad**= Faisalabad, Jhang, Toba Tek Singh, Chiniot; **Gujranwala**= Gujranwala, Gujrat, Narowal, Sialkot, Mandi Baha Uddin, Hafizabad; **Multan**= Multan, Lodhran, Khanewal, Vehari; **Sargodha**= Bhakkar, Khushab, Mianwali, Sargodha; **Sahiwal**= Sahiwal, Okara, Pakpattan; **Dera Ghazi Khan**= Dera Ghazi Khan, Layyah, Muzaffargarh, Rajanpur

For peer review only - http://bmjopen.bmj.com/site/about/guidelines.xhtml

S2 Table Response Rate for Community and Hospital Pharmacists in the nine divisions of Punjab

Sr.	Division in Punjab	Total No. of Community Pharmacies Visited	No. of CPs Present on the 1st visit	Total No. of CPs Administered the Survey by the 2nd visit	No. of CPs Refused	Total No. of CPs Completed the Survey	*Response Rate of the CPs*	Total No. of HPs Administered the Survey	No. of HPs Refused	No. of HPs Completed the Survey	*Response Rate of the HPs*
		n	n (%)	(a)	n	(b)	*(%)*	(c)	n	(d)	*(%)*
1	Lahore	249	146 (58.6)	236	9	227	*96.2*	155	27	128	*82.5*
2	Bahawalpur	66	30 (45.5)	48	4	44	*91.7*	10	5	5	*50*
3	Rawalpindi/ Islamabad	41	22 (53.7)	32	3	29	*90.6*	15	4	11	*73.3*
4	Faisalabad	79	15 (18.9)	31	6	25	*80.6*	18	12	6	*33.3*
5	Gujranwala	181	70 (38.7)	103	7	96	*93.2*	69	14	55	*79.7*
6	Multan	57	24 (42.1)	39	5	34	*87.2*	39	9	30	*76.9*
7	Sargodha	43	18 (41.9)	25	3	22	*88*	33	8	25	*75.7*
8	Sahiwal	34	13 (38.2)	23	2	21	*91.3*	18	7	11	*61.1*
9	Dera Ghazi Khan	14	4 (28.6)	8	2	6	*75*	14	6	8	*57.1*
Overall/ Total		**764**	**342 (44.8)**	**545**	**41**	**504**	***94.98***	**371**	**92**	**279**	***75.2***

***Overall pharmacist response rate** = $\frac{b + d}{a + c} \times 100 = \frac{783}{916} \times 100 = 85.5\%$

HP= Hospital Pharmacist, **CP= Community Pharmacist.

***Each division consists of various districts, for instance:

Lahore= Lahore, Sheikhupura, Kasur, Nankana Sahib; **Bahawalpur**= Bahawalpur, Bahawalnagar, Rahim Yar Khan; **Rawalpindi/Islamabad**= Rawalpindi, Jhelum, Chakwal, Attock; **Faisalabad**= Faisalabad, Jhang, Toba Tek Singh, Chiniot; **Gujranwala**= Gujranwala, Gujrat, Narowal, Sialkot, Mandi Baha Uddin, Hafizabad; **Multan**= Multan, Lodhran, Khanewal, Vehari; **Sargodha**= Bhakkar, Khushab, Mianwali, Sargodha; **Sahiwal**= Sahiwal, Okara, Pakpattan; **Dera Ghazi Khan**= Dera Ghazi Khan, Layyah, Muzaffargarh, Rajanpur

S2 Table Details the individual competency scores against different variables.

Variable	Category	Opioids & Society (C1) Mean (SD)	C1 t (p-value)	Opioids' Physiology (C2) Mean (SD)	C2 t (p-value)	Opioids' Pharmacology (C3) Mean (SD)	C3 t (p-value)	Opioids' Pharmacokinetic & Drug Interaction (C4) Mean (SD)	C4 t (p-value)	Therapeutic use of Opioids (C5) Mean (SD)	C5 t (p-value)	Opioids' Education (C6) Mean (SD)	C6 t (p-value)	Opioids Dispensing (C7) Mean (SD)	C7 t (p-value)	Opioids & Pain Management (C8) Mean (SD)	C8 t (p-value)	Course of Opioid Therapy (C9) Mean (SD)	C9 t (p-value)	Opioids' Use monitoring (C10) Mean (SD)	C10 t (p-value)	Opioid Overdose Management (C11) Mean (SD)	C11 t (p-value)
**Gender**	**Male**	1.45 (0.956)	-0.160 (0.873)	2.05 (1.353)	-0.966 (0.335)	1.95 (1.389)	-1.649 (0.100)	1.88 (1.280)	0.250 (0.803)	1.36 (1.198)	-1.329 (0.184)	2.17 (1.205)	-1.030 (0.303)	2.18 (1.352)	-1.200 (0.231)	1.78 (1.115)	0.048 (0.962)	1.91 (1.376)	1.620 (0.106)	1.42 (1.049)	-0.251 (0.802)	1.49 (1.316)	-0.589 (0.556)
	**Female**	1.46 (0.957)		2.15 (1.388)		2.12 (1.442)		1.86 (1.239)		1.48 (1.244)		2.27 (1.219)		2.30 (1.307)		1.77 (1.083)		1.75 (1.374)		1.44 (1.079)		1.55 (1.286)	
**System of assessment in the Pharm-D degree program**	**Annual**	1.49 (0.995)	1.054 (0.292)	2.17 (1.379)	1.639 (0.102)	2.05 (1.409)	0.826 (0.409)	1.90 (1.257)	0.608 (0.543)	1.39 (1.215)	-0.364 (0.716)	2.22 (1.210)	0.225 (0.822)	2.28 (1.285)	1.104 (0.270)	1.85 (1.098)	1.869 (0.062)	1.87 (1.474)	0.348 (0.728)	1.46 (1.074)	0.735 (0.462)	1.57 (1.350)	1.235 (0.219)
	**Semester**	1.42 (0.919)		2.01 (1.351)		1.97 (1.411)		1.85 (1.273)		1.42 (1.216)		2.20 (1.211)		2.17 (1.379)		1.71 (1.104)		1.84 (1.287)		1.40 (1.047)		1.46 (1.263)	
**Type of institute**	**Public**	1.49 (0.955)	1.144 (0.253)	2.16 (1.339)	1.625 (0.105)	2.02 (1.372)	0.248 (0.805)	1.96 (1.322)	2.066 **(0.039) ***	1.42 (1.244)	0.461 (0.645)	2.21 (1.221)	0.065 (0.948)	1.22 (1.351)	-0.082 (0.935)	1.89 (1.118)	2.939 **(0.003) ***	1.92 (1.479)	1.315 (0.189)	1.49 (1.096)	1.696 (0.090)	1.58 (1.337)	1.573 (0.116)
	**Private**	1.41 (0.956)		2.01 (1.390)		2.00 (1.196)		1.78 (1.196)		1.38 (1.185)		1.21 (1.200)		1.22 (1.322)		1.66 (1.075)		1.79 (1.258)		1.36 (0.016)		1.43 (0.268)	
**Highest level of education**	**Masters**	1.57 (0.960)	1.666 (0.096)	2.43 (1.353)	3.475 **(0.001) ***	2.24 (1.469)	2.259 **(0.024) ***	2.02 (1.351)	1.618 (0.106)	1.85 (1.244)	5.118 **(0.000) ***	2.41 (1.288)	2.173 **(0.031) ***	2.32 (1.371)	1.004 (0.316)	1.92 (1.137)	1.877 (0.061)	2.08 (1.494)	2.189 **(0.030) ***	1.62 (1.065)	2.539 **(0.011) ***	1.55 (1.315)	0.413 (0.680)
	**Pharm-D/ B-Pharm**	1.43 (0.953)		2.00 (1.357)		1.95 (1.390)		1.84 (1.241)		1.30 (1.184)		2.16 (1.186)		2.20 (1.327)		1.74 (1.092)		1.80 (1.341)		1.38 (0.053)		1.50 (1.303)	
**Have you completed any continuous professional development courses in last TWO years?**	**Yes**	1.53 (0.966)	1.230 (0.219)	2.03 (1.385)	-0.586 (0.558)	2.01 (1.526)	0.012 (0.991)	1.94 (1.317)	0.851 (0.395)	1.49 (1.197)	1.008 (0.314)	2.20 (1.239)	-0.103 (0.896)	2.25 (1.440)	0.327 (0.744)	1.76 (1.108)	-0.154 (0.878)	1.75 (1.445)	-1.180 (0.238)	1.40 (1.051)	-0.444 (0.658)	1.33 (1.259)	-2.113 **(0.035) ***
	**No**	1.43 (0.952)		2.10 (1.360)		2.01 (1.375)		1.85 (1.250)		1.38 (1.220)		2.21 (1.203)		2.21 (1.305)		1.78 (1.102)		1.88 (1.356)		1.44 (1.063)		1.56 (1.314)	
**Employment status**	**Full-time**	1.50 (0.955)	2.520 **(0.012) ***	2.14 (1.350)	2.211 **(0.027) ***	2.08 (1.398)	2.581 **(0.010) ***	1.96 (1.264)	3.768 **(0.000) ***	1.46 (1.228)	2.412 **(0.017) ***	2.29 (1.196)	3.483 **(0.001) ***	2.31 (1.350)	3.875 **(0.000) ***	1.82 (1.094)	2.116 **(0.035) ***	1.91 (1.390)	2.162 **(0.031) ***	1.50 (1.076)	3.803 **(0.000) ***	1.55 (1.324)	1.537 (0.125)
	**Part-time**	1.29 (0.941)		1.88 (1.405)		1.76 (1.428)		1.55 (1.219)		1.21 (1.150)		1.92 (1.220)		1.89 (1.232)		1.62 (1.123)		1.65 (1.310)		1.17 (0.958)		1.38 (1.229)	
**If pharmacy is in hospital, type of hospital:**	**Public**	1.67 (0.943)	2.628 **(0.009) ***	2.34 (1.344)	1.417 (0.157)	2.17 (1.429)	0.783 (0.434)	2.02 (1.490)	1.053 (0.293)	1.59 (1.269)	1.242 (0.215)	2.20 (1.292)	-0.282 (0.778)	1.91 (1.233)	-1.126 (0.261)	1.95 (1.181)	1.056 (0.292)	2.15 (1.586)	1.719 (0.087)	1.48 (1.023)	0.620 (0.536)	1.58 (1.306)	0.372 (0.710)
	**Private**	1.35 (0.988)		2.10 (1.284)		2.02 (1.475)		1.84 (1.252)		1.39 (1.195)		2.25 (1.323)		2.10 (1.367)		1.79 (1.043)		1.84 (1.345)		1.39 (1.167)		1.52 (1.253)	
**Type of pharmacy practice**	**Community**	1.39 (0.944)	-2.389 **(0.017) ***	1.99 (1.379)	-2.620 **(0.009) ***	1.95 (1.389)	-1.598 (0.111)	1.83 (1.172)	-1.322 (0.187)	1.34 (1.194)	-2.033 **(0.042) ***	2.20 (1.159)	-0.174 (0.862)	2.36 (1.349)	3.847 **(0.000) ***	1.71 (1.078)	-2.312 **(0.021) ***	1.75 (1.283)	-2.803 **(0.005) ***	1.41 (1.054)	-0.422 (0.673)	1.48 (1.315)	-0.848 (0.397)
	**Hospital**	1.56 (0.968)		2.26 (1.327)		2.12 (1.443)		1.96 (1.416)		1.52 (1.246)		2.22 (1.300)		1.97 (1.279)		1.90 (1.138)		2.05 (1.515)		1.45 (1.071)		1.56 (1.287)	

***Independent t-test, p-value (≥ 0.05) Bold values indicate statistical significance.**

SD= standard deviation, C= competency

DEMOGRAPHICS

Please ENCIRCLE/WRITE

- **Gender:** Male Female
- **Age (years)** ________ **Years of practice experience** _________
- **Employment status:** Full-time Part-time Intern
- **Type of community pharmacy:** Chain Independent Hospital
- **If pharmacy in hospital, type of hospital:** Public Private
- **Highest level of education:** PhD Masters Pharm-D/B-Pharm
- **Location of pharmacy:**
  - Lahore, Kasur, Nankana Sahib, Sheikhupura
  - Gujranwala, Gujrat, Hafizabad, MandiBahauddin, Narowal, Sialkot
  - Sargodha, Khushab, Mianwali, Bhakkar
  - Sahiwal, Pakpattan, Okara
  - Multan, Lodhran, Khanewal, Vehari
  - Faisalabad, Chiniot, Toba Tek Singh, Jhang
  - Rawalpindi, Jhelum, Chakwal, Attock
  - Bahawalpur, Bahawalnagar, Rahim Yar Khan
  - DG Khan, Layyah, Muzaffargarh, Rajanpur
- **Please encircle the appropriate:**

[Never = 1, rarely= 2, Sometimes= 3, Very often= 4, Almost always =5]

- I……………..make clinical notes in a journal/dispensing software (apart from narcotic register) to monitor ongoing opioids use.
  1 2 3 4 5
- I …...counsel patients on the possible side effects of opioids.
  1 2 3 4 5
- I …...counsel patients on opioids over dose risk.
  1 2 3 4 5
- I ……….refuse the supply of the opioids when in doubt of abuse
  1 2 3 4 5
- I………..notify police/regulatory bodies on the abuse of opioids
  1 2 3 4 5
- To clarify the opioids' prescription, I ………..call the physician

[Never = 1, rarely= 2, Sometimes= 3 Very often= 4 Almost always =5]

- **Have you completed any continuous professional development course in last ONE year?** YES NO
- **You completed your B.Pharm/Pharm-D degree from a ……….institute**
  Public Private
- **Your system of education in B.Pharm/Pharm-D was:**
  Annual Semester

OPIOIDS AND SOCIETY

1 **The "Opioid Epidemic" or "Opioid Crises" refers to the increase in the number of deaths and hospitalization:**
   a. Due to prescription opioid use
   b. Due to illicit(illegal) opioid use
   c. Due to opioid use for instant pain relief
   d. Due to both prescription and illicit(illegal) opioid use
   e. I don't know

2 **National Institute on Drug Abuse recommends the term _______ for a "substance or drug abuser"**
   a. Drug addict
   b. Patient
   c. Junkie
   d. Addicted baby
   e. I don't know

3 **In Pakistan, the highest number of opioid drug users belongs to:**
   a. KPK
   b. Punjab
   c. Balochistan
   d. Sindh
   e. I don't know

4 **In Pakistan, opioid misuse is more common among**
   a. Males
   b. Females
   c. Transgender
   d. No difference on the basis of gender
   e. I don't know

5 **In Pakistan, generally women abuse sedative and amphetamines, while, men favor _____**
   a. Methamphetamine and cannabis
   b. Cannabis and alcohol
   c. Sedative and opioids
   d. Cannabis and opioids
   e. I don't know

For peer review only

OPIOID PHYSIOLOGY

1 **Opioids produce analgesia, which indicates:**
   a. CNS depression
   b. CNS stimulation
   c. Both depression and stimulation
   d. No effect
   e. I don’t know

2 **The increase in the sensitivity of pain receptors is called:**
   a. Analgesia
   b. Algesia
   c. Hyperalgesia
   d. Allodynia
   e. I don’t know

3 **Beta endorphins are the most common endogenous ligands for the receptor:**
   a. Beta
   b. Delta
   c. Kappa
   d. Mu
   e. I don’t know

4 **When activated, which one of the following opioid receptors mainly cause both analgesia and dysphoria?**
   a. Mu
   b. Kappa
   c. Delta
   d. Alpha
   e. I don’t know

5 **When activated, which one of the following opioid receptors causes the most respiratory depression?**
   a. Mu
   b. Beta
   c. Kappa
   d. Delta
   e. I don’t know

OPIOID PHARMACOLOGY

1 **The difference between Opiates and Opioids is**
   a. Opiates are natural, while Opioids are synthetic
   b. Opioids are natural, while Opiates are synthetic
   c. Opiates are semi-synthetic, while Opioids are synthetic
   d. Opioids are semi-synthetic, while Opiates are synthetic
   e. I don’t know

2 **Majority of the opioid receptors belongs to:**
   a. Intracellular receptors
   b. Tyrosine Kinase receptors
   c. G-protein coupled receptors
   d. Enzyme linked receptors
   e. I don’t know

3 **Tramadol is a __________ Mu receptor agonist**
   a. Strong
   b. Weak
   c. Moderate
   d. None of these
   e. I don’t know

4 **At the injection site, Morphine causes localized pain and itching due to the release of:**
   a. Glutamine
   b. Serotonin
   c. Dopamine
   d. Histamine
   e. I don’t know

5 **Which one of the following opioids is a mixed agonist-antagonist?**
   a. Morphine
   b. Fentanyl
   c. Codeine
   d. Pentazocine
   e. I don’t know

For peer review only - http://bmjopen.bmj.com/site/about/guidelines.xhtml

OPIOID PHARMACOKINETICS AND DRUG INTERACTIONS

1 **Morphine undergoes _________ during phase-2 biotransformation**
   a. Methylation
   b. Acetylation
   c. Glucuronidation
   d. Oxidation
   e. I don’t know

2 **Which one of the following opioids is safe to use in renal failure and does not require dose adjustment?**
   a. Buprenorphine
   b. Morphine
   c. Fentanyl
   d. Methadone
   e. I don’t know

3 **Which one of the following opioids has the least CNS penetration?**
   a. Morphine
   b. Loperamide
   c. Codeine
   d. Fentanyl
   e. I don’t know

4 **Opioids can cause significant respiratory depression when administered with:**
   a. Naloxone
   b. Naltrexone
   c. Anti-hypertensive
   d. Benzodiazepines
   e. I don’t know

5 **Which of the following drugs when administered with tramadol can cause serotonin syndrome (increase in serotonin levels)?**
   a. Tricyclic anti-depressants
   b. Selective serotonin reuptake inhibitors
   c. Both
   d. None of these
   e. I don’t know

THERAPEUTIC USE OF OPIOIDS

1 **The appropriate treatment for moderate pain as defined by WHO analgesic ladder is:**
   a. NSAIDS
   b. Strong opioids and NSAIDS
   c. Weak opioids and NSAIDS
   d. Weak opioids and strong opioids
   e. I don’t know

2 **Which of the following opioids are used for opioid substitution therapy?**
   a. Meperidine and Methadone
   b. Oxycodone and Buprenorphine
   c. Fentanyl and Methadone
   d. Methadone and Buprenorphine
   e. I don’t know

3 **Opioid dependence refers to:**
   a. Withdrawal symptoms when not taking the opioid
   b. Reduced response to an opioid with repeated use
   c. Persistent use of an opioid despite substantial harm and adverse consequences
   d. Overwhelming desire to continue taking the opioid and obtaining it by all means
   e. I don’t know

4 **Which one of the following opioids is generally contraindicated in Myocardial Infarction?**
   a. Morphine
   b. Fentanyl
   c. Methadone
   d. Pentazocine
   e. I don’t know

5 **Opioid dependence during pregnancy can be treated with:**
   a. Methadone
   b. Fentanyl
   c. Naloxone
   d. Morphine
   e. I don’t know

For peer review only

For peer review only - http://bmjopen.bmj.com/site/about/guidelines.xhtml

OPIOID EDUCATION

1 **Pharmacist should educate the patient to change the fentanyl patch after:**
   a. 72 hours
   b. 48 hours
   c. 24 hours
   d. 6 hours
   e. I don't know

2 **The appropriate fate of fentanyl patch after use is:**
   a. Reuse the same patch
   b. Incinerate(fire) the patch
   c. Flush over (used or leftover patches)
   d. Give to someone else for use to prevent drug wastage
   e. I don't know

3 **Pharmacist should advise the patient to store opioids at:**
   a. Room temperature
   b. Refrigerator
   c. Freezer
   d. Any temperature
   e. I don't know

4 **Pharmacist should restrict methadone supply for:**
   a. 7 days
   b. 8 days
   c. 6 days
   d. 10 days
   e. I don't know

5 **In case of incorrect, ambiguous or incomplete prescription of opioids, pharmacist should:**
   a. Dispense medication as written
   b. Correct the prescription and dispense the medication
   c. Call to police
   d. Hold dispensing and clarify from the prescriber
   e. I don't know

OPIOID DISPENSING

1 **In Pakistan, as per Control of Narcotic Substances Act 1967, opioids come under:**
   a. Schedule A
   b. Schedule B
   c. Schedule C
   d. Schedule D
   e. I don't know

2 **Which one of the following is NOT the sign of opioid's intoxication?**
   a. Altered mental state
   b. Shallow breathing
   c. Irresponsiveness
   d. Fast breathing
   e. I don't know

3 **Pharmacist should dispense the take home doses of opioids in:**
   a. Child resistant containers
   b. Amber colored bottles
   c. Light colored containers
   d. Aluminum foil
   e. I don't know

4 **The record for the sale of opioids should be documented in the narcotic register with:**
   a. blue ink
   b. red ink
   c. green ink
   d. any color
   e. I don't know

5 **As per Punjab Drug Rules-2007, which one of the following is an important particular to mention in the narcotic register during the sale of opioids?**
   a. Lot number
   b. Expiry date
   c. Manufacturing date
   d. Batch number
   e. I don't know

For peer review only - http://bmjopen.bmj.com/site/about/guidelines.xhtml

OPIOIDS AND PAIN MANAGEMENT

1 **…is the pain due to injury to nerve and opioid of choice for this kind of pain is…………**
   a. Neuropathic, Methadone
   b. Psychogenic, Morphine
   c. Somatic, Methadone
   d. Visceral, Morphine
   e. I don't know

2 **Which one of the following opioids is the gold standard for the treatment of acute (severe, accidental) pain?**
   a. Methadone
   b. Fentanyl
   c. Morphine
   d. Tramadol
   e. I don't know

3 **Which one of the following is the opioid of choice in labor pain?**
   a. Meperidine
   b. Pentazocine
   c. Methadone
   d. Buprenorphine
   e. I don't know

4 **The Centers for Disease Control and Prevention recommends the use of opioid therapy in patients with acute (severe) pain for:**
   a. 7-14 days
   b. 3-7 days
   c. 14-28 days
   d. More than 1 month
   e. I don't know

5 **FDA approved opioid for treatment of acute pain in children is:**
   a. Morphine
   b. Codeine
   c. Fentanyl
   d. Methadone
   e. I don't know

COURSE OF OPIOID THERAPY

1 **Opioid taper, Opioid rotation, and Opioid Equianalgesic table refer to _____ , respectively:**
   a. Dose reduction over time, switching between opioids, equivalent to analgesic dose of morphine
   b. Switching between opioids, dose reduction over time , equivalent to analgesic dose of codeine
   c. Dose reduction over time, switching from opioids to non-opioids, equivalent to analgesic dose of morphine
   d. Dose reduction over time, switching between opioids, equivalent to analgesic dose of methadone
   e. I don't know

2 **The appropriate adult dose of Tramadol is:**
   a. 50-100mg for 4-6 hours PRN, with maximum dose up to 400mg/day
   b. 200-300mg for 4-6 hours PRN, with maximum dose up to 500mg/day
   c. 50-100mg for 4-6 hours PRN, with maximum dose up to 500mg/day
   d. 200-300mg for 4-6 hours PRN, with maximum dose up to 400mg/day
   e. I don't know

3 **As per equianalgesic table, _____ oral dose of tramadol is equivalent to 10mg of morphine**
   a. 100mg
   b. 50mg
   c. 200mg
   d. 150mg
   e. I don't know

4 **The indications for an opioid rotation are to:**
   a. Improve analgesia
   b. Reduce adverse effects
   c. Decrease opioid tolerance
   d. All of the above
   e. I don't know

5 **CDC guidelines recommend appropriate opioid tapering as the**
   a. 10% decrease in dose per month
   b. 20% decrease in dose per month
   c. 30% decrease in dose per month
   d. 40% decrease in dose per month
   e. I don't know

For peer review only - http://bmjopen.bmj.com/site/about/guidelines.xhtml

OPIOID MONITORING

1 **5A's of monitoring chronic pain are:**
   a. Activity, analgesia, adverse effects, aberrant behaviors, affect
   b. Activity, algesia, adverse effects, aberrant behaviors, assign
   c. Activity, algesia, adverse effects, adherent behaviors, affect
   d. Action, analgesia, adverse effects, aberrant behaviors, assign
   e. I don't know

2 **The bolus injection of morphine requires monitoring of respiratory rate after every:**
   a. 12 hours
   b. 6 hours
   c. 3 hours
   d. 24 hours
   e. I don't know

3 **Opioid therapy monitoring is recommended ……for patients at low risk of adverse effects:**
   a. Once every 3-6 months
   b. Twice every 3-6 months
   c. Monthly
   d. Weekly
   e. I don't know

4 **Opioid therapy monitoring is recommended ……..for patients at high risk of adverse effects:**
   a. Once every 3-6 months
   b. Twice every 3-6 months
   c. Monthly
   d. Weekly
   e. I don't know

5 **Chronic opioid therapy is the use of opioids daily or nearly daily for at least:**
   a. 30 days
   b. 90 days
   c. 60 days
   d. 180 days
   e. I don't know

OPIOID OVERDOSE TRAINING

1 **The maximum toxic dose of morphine in adult patients is:**
   a. 250mg/day
   b. 500mg/day
   c. 1000mg/day
   d. 100mg/day
   e. I don't know

2 **The usual dose of Naloxone for opioid toxicity is:**
   a. 0.4mg to 2mg IV at 2-3 min intervals
   b. 10 mg IV at 2-3 min intervals
   c. 2.5mg to 5mg at 2-3 min intervals
   d. 7.5 mg IV at 2-3 min intervals
   e. I don't know

3 **The FDA recommends administering IM Naloxone in the**
   a. Upper arm
   b. Buttock
   c. Veins
   d. Intrathecal
   e. I don't know

4 **Three key symptoms of opioid overdose triad include**:
   a. Pinpoint pupils, Slow breathing, Unconsciousness/non-responsiveness
   b. Dilated pupils, Shallow breathing, Unconsciousness/ non-responsiveness
   c. Pinpoint pupils, Fast breathing, Clammy skin
   d. Dilated pupils, Slow breathing, Limp body (difficulty in walk)
   e. I don't know

5 **Which one of the following opioids is resistant to Naloxone?**
   a. Buprenorphine
   b. Morphine
   c. Codeine
   d. Hydromorphone
   e. I don't know

For peer review only - http://bmjopen.bmj.com/site/about/guidelines.xhtml

For peer review only

STROBE Statement—checklist of items that should be included in reports of observational studies.

	Item No.	Recommendation	Page No.	Relevant text from manuscript
**Title and abstract**	1	(*a*) Indicate the study's design with a commonly used term in the title or the abstract	1	Line 1
		(*b*) Provide in the abstract an informative and balanced summary of what was done and what was found	2	Line 39-68
		**Introduction**		
Background/rationale	2	Explain the scientific background and rationale for the investigation being reported	4-5	Line 87-131
Objectives	3	State specific objectives, including any prespecified hypotheses	5	Line 128
		**Methods**		
Study design	4	Present key elements of study design early in the paper	5	Line 135
Setting	5	Describe the setting, locations, and relevant dates, including periods of recruitment, exposure, follow-up, and data collection	5-6	Line 134-151
Participants	6	(*a*) *Cohort study*—Give the eligibility criteria, and the sources and methods of selection of participants. Describe methods of follow-up<br>*Case-control study*—Give the eligibility criteria, and the sources and methods of case ascertainment and control selection. Give the rationale for the choice of cases and controls<br>*Cross-sectional study*—Give the eligibility criteria, and the sources and methods of selection of participants	7	Line 179-189
		(*b*) *Cohort study*—For matched studies, give matching criteria and number of exposed and unexposed		NA
		Case-control study—For matched studies, give matching criteria and the number of controls per case	NA	
Variables	7	Clearly define all outcomes, exposures, predictors, potential confounders, and effect modifiers. Give diagnostic criteria, if applicable	8	Line 199-206
Data sources/ measurement	8*	For each variable of interest, give sources of data and details of methods of assessment (measurement). Describe comparability of assessment methods if there is more than one group	8	Line 199-206
Bias	9	Describe any efforts to address potential sources of bias	6	Line 149-151
Study size	10	Explain how the study size was arrived at	5-6	Line 145-148

For peer review only

Quantitative variables	11	Explain how quantitative variables were handled in the analyses. If applicable, describe which groupings were chosen and why	8, 11	Line 199-206, 258-262
Statistical methods	12	(*a*) Describe all statistical methods, including those used to control for confounding	8-12	Line 199-206, 215-273
		(*b*) Describe any methods used to examine subgroups and interactions	8, 11	Line 199-206, 258-262
		(*c*) Explain how missing data were addressed	7	Line 189
		(*d*) *Cohort study*—If applicable, explain how loss to follow-up was addressed<br>*Case-control study*—If applicable, explain how matching of cases and controls was addressed.<br>*Cross-sectional study*—If applicable, describe analytical methods taking account of sampling strategy	5-6	Line 137-151
		(*e*) Describe any sensitivity analyses	NA	NA
		**Results**		
Participants	13*	(a) Report numbers of individuals at each stage of study—eg numbers potentially eligible, examined for eligibility, confirmed eligible, included in the study, completing follow-up, and analysed	5-6	Line 137-151
		(b) Give reasons for non-participation at each stage	7	Line 189
		(c) Consider use of a flow diagram	NA	NA
Descriptive data	14*	(a) Give characteristics of study participants (eg demographic, clinical, social) and information on exposures and potential confounders	8	Line 216-225
		(b) Indicate number of participants with missing data for each variable of interest	7	Line 189
		(c) *Cohort study*—Summarise follow-up time (eg, average and total amount)	NA	NA
Outcome data	15*	*Cohort study*—Report numbers of outcome events or summary measures over time	*NA*	*NA*
		Case-control study—Report numbers in each exposure category, or summary measures of exposure	*NA*	*NA*
		Cross-sectional study—Report numbers of outcome events or summary measures	*8-12*	*Line* 215-273
Main results	16	(*a*) Give unadjusted estimates and, if applicable, confounder-adjusted estimates and their precision (eg, 95% confidence interval). Make clear which confounders were adjusted for and why they were included	8	Line 199-206
		(*b*) Report category boundaries when continuous variables were categorized	NA	NA
		(*c*) If relevant, consider translating estimates of relative risk into absolute risk for a meaningful time period	NA	NA

Continued on next page

For peer review only - http://bmjopen.bmj.com/site/about/guidelines.xhtml

Other analyses	17	Report other analyses done—eg analyses of subgroups and interactions, and sensitivity analyses	12	Line 264
**Discussion**				
Key results	18	Summarise key results with reference to study objectives	12	Line 276
Limitations	19	Discuss limitations of the study, taking into account sources of potential bias or imprecision. Discuss both direction and magnitude of any potential bias	3, 16	Line 74-85, 366-379
Interpretation	20	Give a cautious overall interpretation of results considering objectives, limitations, multiplicity of analyses, results from similar studies, and other relevant evidence	12	Line 275
Generalisability	21	Discuss the generalisability (external validity) of the study results	16	Line 377
**Other information**				
Funding	22	Give the source of funding and the role of the funders for the present study and, if applicable, for the original study on which the present article is based	18	Line 431

*Give information separately for cases and controls in case-control studies and, if applicable, for exposed and unexposed groups in cohort and cross-sectional studies.

Note: An Explanation and Elaboration article discusses each checklist item and gives methodological background and published examples of transparent reporting. The STROBE checklist is best used in conjunction with this article (freely available on the Web sites of PLoS Medicine at http://www.plosmedicine.org/, Annals of Internal Medicine at http://www.annals.org/, and Epidemiology at http://www.epidem.com/). Information on the STROBE Initiative is available at www.strobe-statement.org.

For peer review only - http://bmjopen.bmj.com/site/about/guidelines.xhtml

LAHORE PHARMACY COLLEGE

(A PROJECT OF LMDC)
Affiliated with
UNIVERSITY OF HEALTH SCIENCES, LAHORE

Ref. No. LPC / ETH/26/09/22 Date: 23-09-2022

Dr. Naeem Mubarak,
Department of Pharmacy Practice,
Lahore Pharmacy College,
(A Project of LMDC).

SUBJECT: **ETHICAL APPROVAL CERTIFICATE**

Dear Dr. Naeem Mubarak,

Apropos of the ethical review application (ref: NM/03//22), after a detailed review of the ethical aspects of the research project mentioned below, the Research Ethics Committee of Lahore Pharmacy College grants ethical approval for the implementation of the study.

- **Research Title:** "Evaluating competencies of Community Pharmacists when supplying opioids. A cross sectional study in Punjab, Pakistan".
- **Principal Supervisor:** Dr. Naeem Mubarak
- **Co-Supervisors:** Prof. Dr. Nasira Saif ur Rehman

This ethical approval certificate is valid from **23rd Sep. 2022** to **23rd Sep. 2023.**

Yours Truly,

Dr. Asad Majeed Khan (PhD)
Chairperson,
Research Ethics Committee,
Lahore Pharmacy College,
University of Health Sciences,
Lahore, Pakistan.
asad.majeed@lmdc.edu.pk

Tulspura, North Canal Bank, Lahore 53400, Pakistan | Tel: +92 346 4418891-98
Fax: +92 42 37392220 | E-mail: laphco@gmail.com | www.lmdc.edu.pk

BMJ Open

"Are Pharmacists on the Frontlines of the Opioid Epidemic? A Cross Sectional Study of the Practices and Competencies of Community and Hospital Pharmacists in Punjab, Pakistan"

Journal:	*BMJ Open*
Manuscript ID	bmjopen-2023-079507.R1
Article Type:	Original research
Date Submitted by the Author:	26-Oct-2023
Complete List of Authors:	MUBARAK, NAEEM; Lahore Medical and Dental College<br>Zahid, Taheer; Lahore Medical and Dental College<br>Rana, Fatima Rahman; Lahore Medical and Dental College<br>Ijaz, Umm-E-Barirah; Lahore Medical and Dental College<br>Shabbir, Afshan; Lahore Medical and Dental College<br>Manzoor, Mahrukh; Lahore Medical and Dental College<br>Khan, Nahan; Lahore Medical and Dental College<br>Arif, Minahil; Lahore Medical and Dental College<br>Naeem, Muhammad Mehroz; Lahore Medical and Dental College<br>Kanwal, Sabba; Lahore Medical and Dental College<br>Saif-ur-Rehman, Nasira; Lahore Medical and Dental College<br>Zin, Che; International Islamic University Malaysia - Kuantan Campus, Pharmacy Practice<br>Mahmood, Khalid; University of the Punjab Quaid-i-Azam Campus<br>Asgher, Javaid; Lahore Medical and Dental College<br>Elnaem, Mohamed Hassan; Ulster University - Coleraine Campus
**Primary Subject Heading**:	Public health
Secondary Subject Heading:	Health policy
Keywords:	Hospitals, PAIN MANAGEMENT, Primary Care < Primary Health Care, PUBLIC HEALTH

SCHOLARONE™
Manuscripts

I, the Submitting Author has the right to grant and does grant on behalf of all authors of the Work (as defined in the below author licence), an exclusive licence and/or a non-exclusive licence for contributions from authors who are: i) UK Crown employees; ii) where BMJ has agreed a CC-BY licence shall apply, and/or iii) in accordance with the terms applicable for US Federal Government officers or employees acting as part of their official duties; on a worldwide, perpetual, irrevocable, royalty-free basis to BMJ Publishing Group Ltd ("BMJ") its licensees and where the relevant Journal is co-owned by BMJ to the co-owners of the Journal, to publish the Work in this journal and any other BMJ products and to exploit all rights, as set out in our licence.

The Submitting Author accepts and understands that any supply made under these terms is made by BMJ to the Submitting Author unless you are acting as an employee on behalf of your employer or a postgraduate student of an affiliated institution which is paying any applicable article publishing charge ("APC") for Open Access articles. Where the Submitting Author wishes to make the Work available on an Open Access basis (and intends to pay the relevant APC), the terms of reuse of such Open Access shall be governed by a Creative Commons licence – details of these licences and which Creative Commons licence will apply to this Work are set out in our licence referred to above.

Other than as permitted in any relevant BMJ Author's Self Archiving Policies, I confirm this Work has not been accepted for publication elsewhere, is not being considered for publication elsewhere and does not duplicate material already published. I confirm all authors consent to publication of this Work and authorise the granting of this licence.

For peer review only

"Are Pharmacists on the Frontlines of the Opioid Epidemic? A Cross Sectional Study of the Practices and Competencies of Community and Hospital Pharmacists in Punjab, Pakistan"

Short title: Opioid-related Competencies among Pharmacists

Naeem Mubarak,[1] Taheer Zahid,[2] Fatima Rahman Rana,[1] Umm-E-Barirah Ijaz,[1] Afshan Shabbir,[1] Mahrukh Manzoor,[1] Nahan Khan,[1] Minahil Arif,[1] Muhammad Mehroz Naeem,[1] Sabba Kanwal,[1] Nasira Saif-ur-Rehman,[3] Che Suraya Zin,[4] Khalid Mahmood,[5] Javaid Asgher,[1,6,7] Mohamed Hassan Elnaem[8]*

[1] Department of Pharmacy Practice, Faculty of Pharmaceutical Sciences, Lahore University of Biological & Applied Sciences, Lahore, Punjab, Pakistan.

[2] Manager, Servaid Pharmacy, 24-M Quaid-e-Azam Industrial Estate, Lahore, Punjab, Pakistan.

[3] Department of Pharmaceutics, Faculty of Pharmaceutical Sciences, Lahore University of Biological & Applied Sciences, Lahore, Punjab, Pakistan.

[4] Kulliyyah of Pharmacy, International Islamic University, Kuantan, Malaysia.

[5] Institute of Information Management, University of the Punjab, Lahore, Punjab, Pakistan.

[6] Lahore Medical & Dental College, Lahore, Punjab, Pakistan.

[7] Doctors Hospital & Medical Centre, Lahore, Punjab, Pakistan.

[8] School of Pharmacy and Pharmaceutical Sciences, Ulster University, Coleraine, United Kingdom.

Corresponding author:
Dr Mohamed Hassan Elnaem,
School of Pharmacy and Pharmaceutical Sciences, Ulster University, Coleraine, United Kingdom.
Email: m.elnaem@ulster.ac.uk
ORCiD: 0000-0003-0873-6541

For peer review only - http://bmjopen.bmj.com/site/about/guidelines.xhtml

ABSTRACT

Introduction

Countries are grappling with a rapidly worsening upsurge in the opioid-related overdose deaths, misuse, and abuse. There is a dearth of data in Pakistan regarding the practices and competencies of pharmacists in handling opioid-related issues.

Study Design

A cross-sectional study, conducted across Punjab, Pakistan.

Method

The study deployed a validated survey to evaluate the competencies and practices of the community and hospital pharmacists.

Results

504 community pharmacists and 279 hospital pharmacists participated in the survey with an overall response rate of 85.5%. Almost half of the respondents "never" or "sometimes" made clinical notes in a journal or dispensing software to monitor ongoing opioid use. Generally, pharmacists were reluctant to collaborate with physicians or notify police regarding the abuse/misuse of opioids. Hospital pharmacists achieved significantly higher Mean Competency Scores than chain and independent community pharmacists ($p<0.05$). In competency evaluation, three priority areas emerged that require additional training i.e., "opioid overdose management", "opioid use monitoring", and "therapeutic uses of opioids".

Conclusion

Both community and hospital pharmacists hold significant positions and potential to contribute meaningfully to the mitigation of harms and risks associated with opioids. Nevertheless, this study underscores notable deficiencies in the competence of pharmacists, whether in hospital or community settings in Punjab, concerning various aspects related to the dispensing and utilization of opioids. It also highlights the pressing need for the development of strategies aimed at improving several practice areas including the documentation, the quality of patient counseling, the effectiveness of reporting mechanisms for opioid abuse, and the stringent enforcement of regulatory policies to curtail opioid misuse. Thus, to mitigate the opioid epidemic in Pakistan, it is imperative to institute opioid stewardship initiatives aimed at rectifying the competency and procedural deficiencies within the pharmacist workforce.

Keywords

Pain Management, Opioid, Opioid Epidemic, Analgesics, Opiate Overdose, Opioid Dispensing, Opioid Competencies, Community Pharmacist, Hospital Pharmacists, Hospitals, Primary care, Public Health, Pakistan.

For peer review only

For peer review only - http://bmjopen.bmj.com/site/about/guidelines.xhtml

- This study is the first of its kind to systematically assess the practices and competencies of community and hospital pharmacists in relation to opioid use and dispensing, utilizing an extensive framework comprising eleven opioid competencies.
- The study stands out for its inclusivity and generalizability, as the sample of the community and hospital pharmacists represent diversely located nine administrative divisions of the Punjab.
- The length of the survey, with 55 items, could have induced respondent fatigue, potentially affecting the accuracy and completeness of the responses.
- We could not comment on the absence of pharmacists in hospital settings due to the absence of data regarding the total number of pharmacists in a given hospital.
- The study excluded 13 incomplete survey forms, the response rate and the diversity of the sample size achieved still support the generalizability of the results.

For peer review only - http://bmjopen.bmj.com/site/about/guidelines.xhtml

INTRODUCTION

Chronic pain is one of the most debilitating human experiences that affects over a quarter of the global population in terms of significant physical discomfort. [1] Opioid analgesics, also known as narcotics, are the primary drugs for pain relief, which strictly require a prescription of a legally qualified prescriber and notable examples include morphine, oxycodone, fentanyl, codeine, and tramadol. World Health Organization (WHO) reserves opioid analgesics mainly for moderate to severe pain and palliative care. [2,3] Opioids interact with their specific receptors in the brain and elsewhere to produce a powerful pain-numbing effect, a sense of invincibility and euphoria. Nevertheless, their benefits are often offset by the risks associated with their use, such as tolerance, opioid use disorder, and overdose-related mortalities especially where opioids were combined with alcohol or anti-anxiety medicine. [4,5] Due to these inherent risks, the dispensing of opioids is subject to stringent regulations and involves meticulous documentation of the patient information, prescriber details, and the exact dose in the narcotic register — a responsibility typically entrusted to a qualified pharmacist. [6,7]

"Opioid crisis" or "opioid epidemic" refers to the alarming upsurge in opioid related overdose deaths and opioid use disorder, primarily due to escalating opioids use/misuse. According to the world drug report, in 2017, around two-thirds of the total drug-related deaths were attributed to opioids while there have been 68, 849.25 confirmed reports of opioid overdose deaths in 2020. [8] In 2017 alone, there were 70,000 opiate related deaths in the US. The opioid epidemic is not confined to specific regions; it is a trend that extends globally. Illicitly manufactured fentanyl are rising in North America, Europe, and Australia. [9] Asian countries are also grappling with the challenges posed by opioid misuse. Asia is the largest contributor to world's illicit opioid use, thus, the opioid epidemic remains a crucial area in global public health research because of its detrimental effect on communities, families, and society, including the socioeconomic burden it imposes on healthcare systems. [10,11]

Pakistan is the 5th most populous country in the world, with its one province Punjab houses a population of 110 million. [12] Concerns have been mounting about the widespread misuse and abuse of prescription opioids in this colossal population. Pakistan is currently at risk particularly due to the imported opioid epidemic from neighboring countries. Opium production and trafficking in Afghanistan, nearly one-third of the world's opioid-using population in India and illegally manufactured opioids in China are among the major drivers. [13,14] In Pakistan, inappropriate dispensing, or inept handling of opioids in community or hospital setting is considered a precipitating factor in the opioid epidemic. It has been estimated that 4.25 million adults in Pakistan use opioids unsafely and 1.6 million people reportedly misuse prescription opioids, mainly tramadol, codeine, and nalbuphine. [14–16] Pharmacists, whether in community or hospital settings, play a crucial role in controlling the supply and

For peer review only - http://bmjopen.bmj.com/site/about/guidelines.xhtml

diversion of opioids by carefully managing opioid refills, preventing individuals from purchasing opioids from multiple pharmacies, avoiding the use of irrational opioid combinations with abuse potential, and monitoring the non-medical use of prescription opioids. [17,18] Further, they have a legal obligation to identify opioid misuse and diversion and collaborate with physicians and law enforcement agencies to report, manage, and screen patients with opioid use disorder. Thus, pharmacists offer a comprehensive range of services to ensure rational opioid use, such as dispensing, counseling, stigma and harm reduction, overdose management, record keeping, recommending replacement therapies, and safe disposal guidance. [6,19] However, efficiently delivering the above-mentioned services requires essential opioid dispensing competencies of the handling pharmacist. Opioid dispensing competencies refer to a pharmacist's knowledge, and skills to promote rational opioid use and reduce non-medical or misuse in society. [20] A recent study gained expert consensus and identified 11 key areas (competencies) for pharmacists on opioid dispensing, based on competency framework devised by the Association of Faculties of Pharmacy of Canada's Opioid Working Group. [20,21] These competencies serve as a foundation for evaluating pharmacists' professional practice standards to promote rational opioid use in each society.

Despite the severity of the opioid epidemic, there has been a dearth of studies evaluating the competence of pharmacists in Pakistan. It is important for pharmacists to ensure they are up to date with opioid dispensing guidelines. Assessing their competency in this area will provide evidence of their readiness to deal with the looming opioid epidemic and identify areas where additional training is needed, to improve their capabilities to serve global health objectives. Therefore, this study aimed to assess and compare community and hospital pharmacists' practices and competencies in dispensing, using, and managing opioids in Punjab, Pakistan.

METHODS

Study Design, Setting & Sample

It was a cross-sectional study that collected data from the community and hospital pharmacists in all nine administrative divisions of the province of Punjab in addition to Islamabad Capital Territory.

According to official sources, there were 23,029 registered pharmacists in Punjab. [22] To obtain the sampling frame for community pharmacies, we formally requested a division-wise list of all licensed community pharmacies in Punjab from the Provincial Drug Control Unit, which provided the required information. The sampling frame encompassed 10,417 community pharmacies. Similarly, the sampling frame for hospital pharmacists was obtained from the primary and secondary health official website. In Punjab, there were 664 hospital pharmacists in public sector hospitals, while when combined with pharmacists in private hospitals

For peer review only

(approximately one-fourth of the population in public hospitals), the sampling frame reached around 830 hospital pharmacists. [23] We used "Raosoft" an online sample size calculator, to compute an appropriate sample size for community and hospital pharmacists. Sample size calculations were based on a 5% margin of error, 95% confidence interval, a response distribution of 50%, and a maximum population of 10,417 (for community pharmacists) and 830 (for hospital pharmacists). The calculator determined a target sample size (n = 371) for community pharmacists and hospital pharmacists (n = 263) to represent their respective populations.

Consequently, to achieve the target sample size, we aimed at a higher number of community and hospital pharmacies, considering potential refusals and dropouts. Further, we used a stratified random sampling technique to reduce the sampling bias and obtain a diverse, representative sample of community and hospital pharmacists.

Survey Instrument

The survey instrument utilized the concept of 11 core opioid dispensing competencies, and survey items were informed by an extensive review of the relevant literature. [14,20,21,24–28]

The survey instrument comprised three main sections:

1- Demographic
2- Routine practices regarding opioid use and dispensing.
3- Core opioid competencies

The third section was further divided into 11 sub-sections corresponding to 11 core opioid competencies. These competencies include understanding societal, physiological, and pharmacological aspects of opioids, recognizing opioid therapeutic applications, and managing opioid overdoses. Knowledge of pain management and adherence to state laws related to opioids was also included. [20,21] Competencies include skills like counselling, communication, and understanding opioid use disorder issues, detecting vulnerable groups for signs of opioid overdose, or misuse is important. Each competency was evaluated using five statements (survey items) which required the respondent to choose the correct answer out of five exclusive options. Total scores were computed using a summative scale for comparisons based on gender, pharmacy practice type, location, and professional development course completion. Please refer to S1 Appendix for the full-length survey. ‘See electronic supplementary material S1 Appendix’.

For peer review only - http://bmjopen.bmj.com/site/about/guidelines.xhtml

Validity and Reliability of the Survey Instrument

After an extensive literature review to develop initial survey items. Six experts, including two professors, two community pharmacists, and two hospital pharmacists, evaluated the face and content validity of the items as per steps mentioned in the Yusoff et al., 2019. [29] They assessed each statement using two different scales: (A) Relevance, with options (1) 'not relevant,' (2) 'somewhat relevant,' (3) 'quite relevant,' or (4) 'highly relevant,' and (B) Clarity, with options (1) 'not clear,' (2) 'somewhat clear,' (3) 'quite clear,' or (4) 'highly clear.' The item-level Content Validity Index (I-CVI) for relevance was calculated as I-CVI (R) = Nr/N, where Nr represents the number of experts who rated an item as (3) or (4), and N is the total number of experts involved. Statements with an I-CVI in the range of 0.83 to 1.00 were deemed acceptable, while those below 0.83 were rejected. This process resulted in a reduction from the initial 70 statements (items) in the questionnaire to a final version comprising 55 items divided in eleven domains related to opioids competency. Moreover, to ensure the internal consistency/ reliability of the survey items, a pilot study involving 50 pharmacists (33 community pharmacists and 17 hospital pharmacists) was conducted, and Cronbach's alpha statistics were applied. Results of the reliability analysis showed that the items in the 11 competencies had a satisfactory level (0.7-0.9) of internal consistency ('See electronic supplementary material S1 Table').

Definitions, Inclusion, and Exclusion Criteria

In context of this study, a pharmacy within the premises of a hospital was referred to as a hospital pharmacy, which can further be categorized based on the type of hospital they are associated with, such as public or private hospitals. On the other hand, any pharmacy outlet outside the premises of a hospital was defined as a community pharmacy. While the existing scholarly work lacks a consensus-based definition of a chain or an independent pharmacy. Chain pharmacies are typically a corporation with 6-200+ pharmacies operating under a nationwide banner. On the other hand, an independent pharmacy follows a small operator model with an individual owning up to five pharmacies. Thus, for our research, we defined a pharmacy as a chain if more than five pharmacies were operating under the same name and the business used distinctive branding across all pharmacies. Detailed inclusion/exclusion criteria are given in Table 1.

For peer review only - http://bmjopen.bmj.com/site/about/guidelines.xhtml

Table 1 Inclusion-Exclusion criteria

Inclusion	Exclusion
Any community or hospital pharmacy registered under a category-A license, located in any of the nine divisions of Punjab or Islamabad Capital Territory was eligible to be included in the study.	Pharmacies without a category-A license or medical stores with category-B license, or designated dispensaries were excluded from data collection.
Pharmacists registered in category-A (legally permitted to handle opioids) were included.	Assistant pharmacist or pharmacy technicians or pharmacy interns (who had not completed the Pharm-D degree) were excluded.
Secondary or tertiary care hospitals both in public and private sector were included	Primary care clinics/facilities were excluded because generally the facility is smaller and do not have a pharmacist within the premises especially in the private sector.
Survey forms completed in all respects were included in the final count	Incomplete/missing survey forms were excluded.

Data Collection and Reporting

The first author conducted a comprehensive training of a team of Pharm-D students and research associates on different aspects of data collection. The team was divided into subgroups assigned to different divisions of Punjab. Data collectors were instructed to pay a second visit to a community pharmacy/ a hospital pharmacy, if the pharmacist was unavailable, or the pharmacy was occupied in rush hours during the first visit. The survey was administered in a face-to-face meeting. Data collection began on December 10, 2022, and ended on January 15, 2023. The data were reported in accordance with STROBE guidelines ('See electronic supplementary material S2 Appendix').

Data Management and Statistical Analysis

We used Statistical Package Social Sciences (version 22 IBM, California, USA) for data management and statistical analysis. Descriptive statistics were used to evaluate categorical variables, while differences in mean scores were compared between two groups for different variables using a t-test. To conduct quantitative analysis, we made groups based on different variables, such as gender, nature of the employment, and type of pharmacy practice. To determine the mean difference among three or more groups, we conducted One-way ANOVA followed by post-hoc analysis. The significance level for detecting differences in means was set at a p-value of ≤ 0.05.

For peer review only - http://bmjopen.bmj.com/site/about/guidelines.xhtml

Ethical Approval

Research Ethics Committee, Lahore Pharmacy College, Lahore Medical & Dental College granted ethical approval for this study (ref# LPC/ETH/25/09/22, 'See electronic supplementary material S3 Appendix'). Before data collection, the research team explained the study objectives to potential participants and obtained their voluntary consent. The research team assured anonymity of the response. Finally, neither the authors nor the data collectors had access to identifying the survey respondents at any research stage.

Patient and Public Involvement

No patient was involved in this survey.

RESULTS

Data were collected from community and hospital pharmacies diversely located in all administrative divisions of the province of Punjab, namely Lahore (provincial capital), Sargodha, Multan, Faisalabad, Bahawalpur, Rawalpindi, Sahiwal, and Dera Ghazi Khan besides Islamabad Capital Territory. During the first visit, pharmacists were present in only 44.8 % of the pharmacies visited. This presence of pharmacists was further declined when the research team moved from the provincial capital to other cities, and the lowest presence (18.9%) was recorded in Faisalabad. However, the presence increased significantly on the second visit. A total of (n=783) pharmacists completed the survey (community pharmacist= 504; hospital pharmacist = 279). Most respondents were full-time employed males within the age range of 26-30. A mere 22.6% of the pharmacists reported involvement in any continuous professional development program within the past two years. Table 2 provides a summary of the demographics of the survey participants.

For peer review only - http://bmjopen.bmj.com/site/about/guidelines.xhtml

Table 2 Demographics of the survey participants

Variable	Category		n (%)	
**Gender**	Male		503 (64.2)	
	Female		280 (35.8)	
**Age group**	23-25		190 (24.3)	
	26-30		367 (46.9)	
	31-35		139 (17.8)	
	36-40		57 (7.3)	
	41-45		19 (2.4)	
	46-50		9 (1.1)	
	56-60		2 (0.3)	
	Total		783 (100)	
**You studied/completed Pharm-D/B-Pharm degree in a ________ institute.**	Public		403 (51.5)	
	Private		380 (48.5)	
**Mode of assessment in your Pharm-D/B-Pharm was:**	Annual		366 (46.7)	
	Semester		417 (53.3)	
**Your highest level of education**	Masters		155 (19.8)	
	Pharm-D/B-Pharm		628 (80.2)	
**Have you completed any continuous professional development course in the last TWO years?**	Yes		177 (22.6)	
	No		606 (77.4)	
**Employment status**	Full-time		614 (78.4)	
	Part-time		169 (21.6)	
**Type of pharmacy practice**	Community	Chain	291 (37.2)	504 (64.4)
		Independent	213 (27.2)	
	Hospital	Public	187 (67)	279 (35.6)
		Private	92 (33)	

n= number

The overall response rate collectively for community and hospital pharmacists was 85.5%, as shown in S2 Table ('See electronic supplementary material S2 Table'). However, it is essential to note that many pharmacists (mostly in hospitals) declined to participate in the survey due to reasons, such as a busy schedule, lack of motivation, or fear of potential regulatory consequences.

For peer review only - http://bmjopen.bmj.com/site/about/guidelines.xhtml

The survey also examined the trends of professional practices on opioid use and dispensing among community and hospital pharmacists. Half of the respondents mentioned that they "never" or "sometimes" made clinical notes in a journal or dispensing software to monitor ongoing opioid use. Roughly one-third of the respondents reported that they "almost always" counsel patients on the possible side effects and overdose risks associated with opioid use. Similarly, only 18% reported that they "almost always" contacted police or physicians regarding the abuse of opioids or clarify an opioid prescription. Table 3 presents the trends of practice among pharmacists.

Table 3 Practices among pharmacists on opioid use

Statements	Never n (%)	Rarely n (%)	Sometimes n (%)	Very Often n (%)	Almost Always n (%)
**I______ make clinical notes in a journal/dispensing software (apart from the narcotic register) to monitor ongoing opioids use.**	179 (22.9)	166 (21.2)	178 (22.7)	121 (15.5)	139 (17.8)
**I ______ counsel patients on the possible side effects of opioids.**	26 (3.3)	89 (11.4)	187 (23.9)	217 (27.7)	264 (33.7)
**I ______ counsel patients on opioids overdose risk.**	40 (5.1)	86 (11.0)	184 (23.5)	189 (24.1)	284 (36.3)
**I ______ refuse the supply of the opioids when in doubt of abuse.**	36 (4.6)	75 (9.6)	105 (13.4)	161 (20.6)	406 (51.9)
**I ______ notify police/regulatory bodies on the abuse of opioids.**	203 (25.9)	154 (19.7)	145 (18.5)	135 (17.2)	146 (18.6)
**To clarify the opioids prescription, I ______ call the physician.**	76 (9.7)	140 (17.9)	294 (37.5)	129 (16.5)	144 (18.4)

n= number

Statistical analysis revealed no significant difference in the overall mean competency scores against the variables, such as gender, community vs. hospital settings, or system of assessment used in the Pharm-D degree program or type of hospital (private or public). In contrast, full-time employed pharmacists with a higher degree after Pharm-D, as well as those who had completed continuous professional development courses, achieved significantly higher mean competency scores. Similarly, pharmacists with a degree from a public university scored slightly higher (significance at borderline) than pharmacists who received a Pharm-D degree from a private educational institute. Table 4 summarizes the overall mean competency score against each variable.

For peer review only - http://bmjopen.bmj.com/site/about/guidelines.xhtml

Table 4 Different variables against the summative mean score of all the core opioids competencies

Variable	Category	Mean (SD)	t* (p-value)
**Gender**	Male	19.64 (8.301)	-0.816 (0.415)
	Female	20.14 (8.019)	
**System of assessment in your Pharm-D degree program**	Annual	20.25 (8.455)	1.393 (0.164)
	Semester	19.44 (7.959)	
**Type of institute**	Public	20.36 (8.700)	1.921 (0.055) *
	Private	19.24 (7.602)	
**Highest level of education**	Masters	21.99 (9.099)	**3.408 (0.001)** *
	Pharm-D/B-Pharm	19.28 (7.878)	
**Employment status**	Full-time	20.50 (8.180)	**4.519 (0.000)** *
	Part-time	17.33 (7.800)	
**Have you completed any continuous professional development course in the last TWO years?**	Yes	20.86 (9.101)	**2.079 (0.038)** *
	No	19.41 (7.787)	
**Type of pharmacy practice**	Community	19.40 (7.869)	-1.846 (0.065)
	Hospital	20.57 (8.730)	
**If pharmacy is in hospital, type of hospital:**	Public	21.05 (9.047)	1.316 (0.189)
	Private	19.59 (8.004)	

***Independent t-test, p-value (≥0.05) Bold values indicate statistical significance.**

SD= standard deviation

On the other hand, results of ANOVA revealed that statistically significant differences (p=0.006) exist among the three pharmacist groups compared, i.e., hospital, chain, and independent pharmacist. Hospital pharmacists secured higher mean competency scores than all other sub-groups (both chain and independent). Further, the mean competency scores for pharmacists at chain pharmacies were better than pharmacists at independent pharmacies, as shown in Table 5.

For peer review only - http://bmjopen.bmj.com/site/about/guidelines.xhtml

Table 5 Comparison of different types of pharmacy practice

Type of Pharmacy Practice	Groups	Mean Difference	p-value
**Chain**	Independent	1.904	**.010 ***
	Hospital	-0.357	.602
**Independent**	Chain	-1.904	**.010 ***
	Hospital	-2.261	**.002***
**Hospital**	Chain	0.357	.602
	Independent	2.261	**.002 ***

***p-value (≤ 0.05)**

S3 Table details the mean score for each of the eleven individual opioid competencies against different variables ('See electronic supplementary material S3 Table'). Comparatively, among the eleven opioid competencies, community pharmacists received the lowest mean competency score in "therapeutic use of opioids" (C-5= 1.34), "opioid and society" (C-1 = 1.39), and "opioid use monitoring" (C-10= 1.41) thus emerged as a priority area to focus for further training. For hospital pharmacists, "opioid use monitoring" (C-10= 1.45), "therapeutic use of opioids" (C-5= 1.52), "opioid and society" (C-1 = 1.56), and "opioid overdose management" (C-11 = 1.56) had the lowest mean scores.

DISCUSSION

The findings of this study portray a bleak picture of the current professional practices and competencies of pharmacist workforce related to opioid use and dispensing in Punjab, Pakistan.

First, it is pertinent to mention that pharmacist availability, especially in the community setting, was challenging during the first visit to pharmacies. Our study confirms the earlier findings of Bashir and colleagues, which reported absence of pharmacists in 50% of the pharmacies in different regions of Punjab. [30] Legally, only a registered pharmacist can dispense opioids in Pakistan. It is not some kind of a delegated responsibility, but an inherent role of pharmacist as defined through an act of law. [13] Pharmacies without a qualified pharmacist or operating on a proxy could precipitate an upsurge in the dispensing of opioids without a prescription and pervasive misuse and abuse of prescription opioids. Further, this finding implies poor regulatory check and balance and raises serious concerns on the consequences of low engagement of community pharmacists in opioid

For peer review only - http://bmjopen.bmj.com/site/about/guidelines.xhtml

management. Thus, pharmacists, especially in community settings, are not visible at the frontline. The government should ensure the implementation of the mandatory presence of a qualified person (pharmacist) in community pharmacies in Punjab. The loopholes in the current regulatory policies and the partial implementation of the Control of Narcotic Substances Act 1967 are also attributed to opioid misuse and unauthorized dispensing. A few countries, including India, Ukraine, and Colombia, have recently amended their laws to engage community pharmacists to offer opioid stewardship activities, though the implementation is slow. [10,31]

Second, findings of this study reveal a potential gap in the documentation practices, which are crucial for ensuring patient safety and continuity of care. Nevertheless, studies support the exigent need for community pharmacist engagement to facilitate the implementation of safe opioid use in society. [6,18] This situation is further exacerbated by the low engagement of pharmacists in professional development programs, which emphasizes the need for initiatives to make continuous education and training programs a regulatory prerequisite for practice. Currently, continuing education for pharmacists is not mandatory in Pakistan. Our findings strongly suggest that making continuing education mandatory for pharmacists could lead to an overall improvement in the quality of healthcare delivery, particularly in the context of opioid dispensation. Another recent research also endorsed that prior training results in better competency of community pharmacists in handling medication opioid use disorder. [15] Thus, community and hospital pharmacists must undergo continuous professional education to dispense opioids and follow standard guidelines.

Third, unfortunately, only one-third of the pharmacists reported that they "almost always" counseled patients on the possible side effects and overdose risks associated with opioid use. While pharmacists have the professional scope to educate patients about opioid-related risks and benefits, suggest alternatives, conduct medication reviews as part of an interdisciplinary team, and counsel on opioid substitution therapy and management of opioid poisoning.. [6,32] These findings may be correlated with the recent surge in the abuse of opioid analgesics, particularly Nalbuphine, in Pakistan. [15] Another study in Pakistan also attributed unchecked dispensing at pharmacies with much of the opioid's abuse and misuse in Pakistan. Thus, to curtail the opioid related overdose mortalities, enhanced patient education especially in the community setting could improve the opioid use in the society.

Fourth, pharmacists were reluctant to call the physician to clarify opioid-related prescriptions, indicating a lack of interprofessional collaboration between pharmacists and general practitioners. These findings indicate the importance and urgent need to enhance interprofessional communication and collaboration, especially in community settings. The decision-making process for a pharmacist in determining whether to contact the police

For peer review only - http://bmjopen.bmj.com/site/about/guidelines.xhtml

or the prescriber in response to a suspicious or potentially problematic opioid prescription is a multifaceted one. Contacting the police will be the last reserve for illegal suspicious orders that have not proven to be authentic upon validation with the prescriber. It is not an option in every case to contact the police vs prescriber. Thus, there are no rigid, one-size-fits-all guidelines for when to contact the police versus the prescriber regarding an opioid prescription, however, there are general principles and practices that guide pharmacists in making this critical decision. It is important to note that these principles are contextual and may vary depending on local regulations, institutional policies, and individual judgment. [13,14] The safety and well-being of the patient will always be the paramount consideration. Pharmacists generally evaluate the prescription's legitimacy and appropriateness for the patient's medical condition. If there is a reasonable suspicion that the prescription may pose a risk to the patient's health or well-being, contacting the prescriber to seek clarification is usually the first step. Pharmacists are trained to recognize signs of forged or fraudulent prescriptions. If there are indications that the prescription is not legitimate or has been altered, it is advisable to contact the prescriber to validate its authenticity. Pharmacists are encouraged to assess the prescribing history of a patient, especially if they notice a recurring pattern of early refills, high doses, or multiple prescriptions from different prescribers. Such patterns may raise red flags and necessitate further investigation or contact with the prescriber. [13,14] An Australian study identified that authentication of the prescription by contacting the prescriber as one of the crucial strategies used in prescription opioid monitoring. [17] Ensuring appropriate opioid use requires devising a mechanism or channel of communication between the prescriber and pharmacist, especially in a community setting in Pakistan. Numerous studies provide evidence for the positive outcomes of collaborative care on medication reviews, identifying prescribing errors, and intervening in opioid misuse in other countries. [33,34] Similarly, only a handful of respondents reported that they notified the police or regulatory bodies regarding the abuse of opioids, highlighting a potential lack of awareness or reluctance to report such cases. The collaboration of pharmacists with law enforcement agencies, opioid use disorder consultants, policymakers, regulators, epidemiologists, and pharmaceutical companies is equally important.

Fifth, higher competency scores of hospital pharmacists compared with chain and independent pharmacies may be linked to their higher exposure and clinical and patient care experience in hospital settings. Similarly, better competency scores at chain pharmacies compared to their counterparts in independent pharmacies could be attributed to the fact that chain pharmacies hold more financial resources, which they spent on the training and development of their pharmacists.

For peer review only - http://bmjopen.bmj.com/site/about/guidelines.xhtml

Finally, the pharmacist who had studied under the current Pharm-D curriculum in Pakistan lack sufficient competencies to practice across all scopes of opioid management and dispensing. The lowest overall mean competency scores in "opioid overdose management", "opioid use monitoring", and "therapeutic use of opioids" indicate the top three priority areas which require further training and education. These findings align with the conclusion drawn in a recent qualitative study on the dearth of skill-based competency among pharmacists, such as identifying and communicating prescription errors, therapeutic duplication, monitoring unauthorized dispensing, and providing patient-centered care. [15] A Canadian study also validated similar key areas of training in opioid and pain management, alternative pain management, naloxone dispensing, collaborating with physicians, and counseling patients on opioid use and misuse. [35] Another possible reason for these poor competency scores may be related to the limited coverage of these topics in the Pharm-D curriculum in Pakistan. Further, the Pharm-D curriculum in Pakistan has not been revised for a decade, making it difficult for fresh graduates to adapt to the updated guidelines on pain management and opioid use. [36] This highlights the urgent need to upgrade the Pharm-D curriculum and incorporate opioid learning modules with updated guidelines. Lessons should be learned from the case of the US, where, in line with the recent opioid epidemic, pharmacy schools are incorporating specific opioids related competencies into their Doctor of Pharmacy (Pharm-D) curriculums to ensure that students are well-prepared to handle the complex issues surrounding opioid use and misuse. [37,38]

Implications for Policy, Practice and Future Research

Based on the findings of this study, the authors suggest the following implications for policy, practice, and further research:

1. It is crucial to address the gaps in knowledge and practice related to therapeutic use of opioids, opioid use monitoring, and opioid overdose management. Improving pharmacist competency in these areas could contribute to the safe and effective use of opioids, thereby reducing the risks associated with their misuse and abuse.
2. Strategies should be developed to promote the documentation of clinical notes and enhanced patient counselling on opioid risks.
3. The notification of opioid misuse to regulatory authorities and clarification of opioid-related prescription from the physicians should be made a binding requirement.
4. To curb opioid misuse, the availability of a qualified personnel at community pharmacist should be ensured.

For peer review only - http://bmjopen.bmj.com/site/about/guidelines.xhtml

5. Community and Hospital pharmacists must pass a licensing exam that assess the different opioid dispensing competencies of pharmacists before recruitment in community and hospital settings.
6. An electronic national opioid database system should be developed to store the records of all opioid prescriptions. This will assist in tracing the unauthorized prescribing and dispensing of opioids.
7. The Pharm-D curriculum should be revised, and opioids must be covered in depth at the graduate and undergraduate level primarily related to opioid use disorder, misuse, and diversion. A detailed and comprehensive module of opioid use can be integrated in pharmacology, clinical pharmacy, and forensic pharmacy. The module's components can be decided upon various consensus methods, such as Delphi and nominal group technique etc.
8. Future research should prioritize the examining of policies and interventions aimed at ensuring the active participation of pharmacists in community pharmacies. Furthermore, there is a need for research to focus on the development of evidence-based opioid stewardship interventions, clinical support tools and collaborative practice models fostering enhanced communication among the pharmacist, prescribers, and regulatory bodies.

For peer review only - http://bmjopen.bmj.com/site/about/guidelines.xhtml

Strength & Limitations

The main strengths of this work are related to being the pioneer study to report the practices and competencies of community and hospital pharmacists on opioid use and dispensing in a systematic way i.e., under the framework of eleven opioid competencies. It thus may be taken as a call for action. Our study did not select a convenient sample of the community pharmacies and hospital pharmacists, instead, it reached out to all nine diverse divisions of Punjab to present data representative of the population.

The current study should be interpreted in the light of its limitations. It was a long survey consisting of 55 items that may have resulted in the fatigue bias of the respondent. However, it was inevitable in the context of the coverage of essential 11 core competencies related to opioid use and dispensing. In the case of community pharmacy, the presence percentage was possible because one pharmacist/pharmacy generally follows the law of the land. However, we could not comment on the absence of pharmacists in hospital settings due to the absence of data regarding the total number of pharmacists in a hospital. We did not include 13 incomplete survey forms, however, given the response rate, diverse and vast sample size achieved, the findings remain the representative of the population and results are expected to be generalizable.

CONCLUSION

While the opioid epidemic continues to inflict grave and unsettling consequences, both community and hospital pharmacists hold significant positions and potential to contribute meaningfully to the mitigation of harms and risks associated with opioids at both individual and societal levels. Nevertheless, this study underscores notable deficiencies in the competence and practices of pharmacists, whether in hospital or community settings in Punjab, concerning various aspects related to the dispensing and utilization of opioids. It also highlights the pressing need for the development of strategies aimed at improving several practice areas including the documentation, the quality of patient counseling, the effectiveness of reporting mechanisms for opioid abuse, and the stringent enforcement of regulatory policies to curtail opioid misuse. Thus, to combat the looming opioid epidemic in Pakistan, it is crucial to equip pharmacists with the necessary competencies to offer opioid stewardship activities to ensure responsible opioid use. However, achieving this goal necessitates a forward-thinking approach, an eagerness to collaborate and notify physicians and regulatory authorities, a willingness to honestly introspect and acknowledge the lack of competencies or past failed efforts, and an investment in the training of pharmacy human resources. Embracing this life-saving commitment is imperative for the well-being of the patients, communities, and the pharmacy profession.

For peer review only - http://bmjopen.bmj.com/site/about/guidelines.xhtml

DECLARATIONS

Competing Interest

The authors have no relevant financial or non-financial interests to disclose.

Data Sharing Statement

All relevant data, materials, and information necessary to understand, replicate, and build upon the findings presented in this article have been made available within the main manuscript and supplementary files associated with this publication.

Acknowledgement

We extend our heartfelt thanks to the Provisional Drug Control Unit, Punjab, for providing the list of community pharmacies across Punjab.

Author Contribution

Naeem Mubarak: Conceptualization, Methodology, Formal analysis, Writing - original draft preparation, Writing - review and editing, Resources, Supervision.

Taheer Zahid: Methodology, Investigation, Resources, Writing - review and editing.

Fatima Rahman Rana: Methodology, Investigation, Formal analysis, Writing - original draft preparation, Writing - review and editing, Resources.

Umm-E-Barirah Ijaz: Methodology, Investigation, Resources, Writing - review and editing.

Afshan Shabbir: Methodology, Investigation, Resources, Writing - review and editing.

Mahrukh Manzoor: Methodology, Investigation, Resources.

Nahan Khan: Methodology, Investigation, Resources, Writing - review and editing.

Minahil Arif: Methodology, Investigation, Resources, Writing - review and editing.

Muhammad Mehroz Naeem: Methodology, Investigation, Resources, Writing - review and editing.

Sabba Kanwal: Methodology, Formal analysis, Resources, Writing - review and editing.

Nasira Saif-ur-Rehman: Methodology, Investigation, Resources, Supervision, Writing - review and editing.

Che Suraya Zin: Methodology, Investigation, Writing - review and editing, Resources, Supervision.

Khalid Mehmood: Methodology, Formal analysis, Writing - review and editing, Resources, Supervision.

Javaid Asgher: Methodology, Investigation, Writing - review and editing, Resources, Supervision.

Mohamed Hassan Elnaem: Methodology, Formal analysis, Writing - review and editing, Resources, Supervision.

For peer review only

467 **Prior Publication**

468 This paper has not been submitted elsewhere in similar form, and all authors have contributed significantly to the
469 publication. All authors are aware of the submission and agree with it.

470

471 **Funding**

472 The authors declare that no funds, grants, or other support were received during the preparation of this manuscript.

473

474 **SUPPORTING INFORMATION**

475 **S1 Table** = Highlights Cronbach's alpha values against individual opioid competencies

476 **S2 Table=** Response Rate for Community and Hospital Pharmacists in the nine divisions of Punjab

477 **S3 Table** = Details the individual competency scores against different variables

478 **S1 Appendix**= Outlines the final version of the survey administered

479 **S2 Appendix**= Provides STROBE guidelines

480 **S3 Appendix**= Depicts Ethical approval document

481

For peer review only

For peer review only - http://bmjopen.bmj.com/site/about/guidelines.xhtml

REFERENCES

1. Macchia L, Oswald AJ. Social Science & Medicine Physical pain , gender , and the state of the economy in 146 nations. Soc Sci Med [Internet]. 2021;287(December 2020):114332. Available from: https://doi.org/10.1016/j.socscimed.2021.114332
2. Amaechi O, Human MM, Featherstone K. Pharmacologic Therapy for Acute Pain. Vol. 104, American Family Physician. 2022.
3. Anekar AA, Hendrix JM, Cascella M. WHO Analgesic Ladder. Stat Pearls Publishing. 2023. p. 1–7.
4. Rauf U, Ali M, Dehele I, Paudyal V, Elnaem MH, Cheema E. Causes, nature and toxicology of fentanyl-analogues associated fatalities: A systematic review of case reports and case series. J Pain Res. 2021;14:2601–14.
5. Alrashdi S, Paudyal V, Elnaem MH, Cheema E. Causes, Nature and Toxicology of Tramadol-Associated Deaths Reported in the Peer-Reviewed Literature: A Systematic Review of Case Studies and Case Series. Drugs Ther Perspect [Internet]. 2021;37(9):422–32. Available from: https://doi.org/10.1007/s40267-021-00859-6
6. Gregory T, Gregory L. The Role of Pharmacists in Safe Opioid Dispensing. J Pharm Pract. 2020;33(6):856–62.
7. Jantarada C, Silva C, Guimarães-Pereira L. Prevalence of Problematic Use of Opioids in Patients with Chronic Noncancer Pain: A Systematic Review with Meta-analysis. Pain Pract. 2021;21(6):715–29.
8. UNODC, World Drug Report 2022 (United Nations publications, 2022)
9. Gardner EA, McGrath SA, Dowling G, Bai D. The Opioid Crisis: Prevalence and Markets of Opioids. Forensic Sci Rev. 2022;34(1):44–70.
10. Zachary, Z., Kathryn, F., Hanna, GP., Anna Z. A global study of pain prevalence across 52 countries : examining the role of country- level contextual factors : PAIN. Pain. 2022;163(9):1–12.
11. Taylor J, Pardo B, Hulme S, Bouey J, Greenfield V, Zhang S, et al. Illicit synthetic opioid consumption in Asia and the Pacific: Assessing the risks of a potential outbreak. Drug Alcohol Depend [Internet]. 2021;220(October 2020):108500. Available from: https://doi.org/10.1016/j.drugalcdep.2020.108500
12. Mubarak N. Corona and clergy – The missing link for effective social distancing in Pakistan: Time for some unpopular decisions. Int J Infect Dis. 2020;95(January):431–2.
13. Siddiqui A, Shrestha S, Ahmed A, Mazhar S, Bhutta OA. Addressing the opioid epidemic in Pakistan: urgent need for opioid stewardship and comprehensive solutions. Drugs Ther Perspect. 2023;1–5.
14. Islam M. State of the art treatment options for Pakistan's opioid, alcohol and methamphetamine crisis. J Pak Med Assoc. 2020;70(6):1063–8.
15. Iqbal A, Knaggs R, Anderson C, Shean L. Logic model for opioid safety in chronic non ‑ malignant pain management , an in ‑ depth qualitative study. Int J Clin Pharm [Internet]. 2022;45(1):220–32. Available from: https://doi.org/10.1007/s11096-022-01493-6
16. Iqbal MJ, Ali M, Rizwan, Ghafoor, Khan AM, Raza MM. Study the different incidences of Tramadol in different cities of Punjab. Vol. 11. 2021.
17. Alvin M, Picco L, Wood P, Mnatzaganian G, Nielsen S. Community pharmacists ' preparedness to intervene with concerns around prescription opioids : findings from a nationally representative survey. Int J Clin Pharm [Internet]. 2020;43(0123456789):411–9. Available from: https://doi.org/10.1007/s11096-020-01152-8
18. Webb K, Cernasev A, Li MS, Gatwood J, Cochran G, Hohmeier KC. An Exploratory Study of Pharmacist Perceptions of Opioid Interventions for Acute Pain. J Pharm Technol. 2021;37(1):36–44.
19. Underwood N, Cremer L, Cance JD, Williams J, Guy GPJ, Zule W. The impact of community-level prevention strategies on high-dose opioid dispensing rates: 2014-2019. Drug Alcohol Depend. 2021 Oct;227:108988.
20. Maher E, Nielsen S, Summers R, Wood P. Core competencies for Australian pharmacists when supplying prescribed opioids: a modified Delphi study. Int J Clin Pharm. 2021 Apr;43(2):430–8.
21. Sproule B. AFPC Commitment to the Joint Statement of Action to Address the Opioid Crisis in Canada. 2018.
22. Punjab Pharmacy Council (2023) https://punjabpharmacycouncil.com/index_files/Page528.htm-(Accessed on-1-7-2023)
23. Punjab, G. of. (2023). Primary & Secondary Health Care Department (pp. 1–71). https://pshealthpunjab.gov.pk/-(Accessed on 1-7-2023)
24. Brady JE, Wunsch H, DiMaggio C, Lang BH, Giglio J, Li G. Prescription drug monitoring and dispensing of prescription opioids. Public Health Rep. 2014;129(2):139–47.
25. Rosen DM, Alcock MM, Palmer GM. Opioids for acute pain management in children. Anaesth Intensive Care. 2022;50(1–2):81–94.
26. Pattullo GG. Opioids in acute pain: Towards getting the right balance. Anaesth Intensive Care. 2022;50(1–

For peer review only - http://bmjopen.bmj.com/site/about/guidelines.xhtml

2):68–80.
27. E.Hall J. Guyton and Hall: Textbook of Medical Physiology. 12th editi. William Schmitt; Rebecca Gruliow; Frank Morales, editor. Saunders. Saunders; 2013. 1112 p.
28. Laurence L. Brunton; Randa Hilal-Dandan; Björn C. Knollmann. Goodman & Gilman's: The Pharmacological Basis of Therapeutics [Internet]. 13th editi. Randa Hilal-Dandan; Bjorn Knollmann, editor. Mc Graw Hill Education; 2022. 1423 p. Available from: https://accesspharmacy.mhmedical.com/book.aspx?bookID=2189
29. Yusoff MSB. ABC of Content Validation and Content Validity Index Calculation. Educ Med J. 2019;11(2):49–54.
30. Bashir I, Ahmad M, Jamshaid M, Zaman M. Heliyon Illicit sale of controlled drugs at community pharmacy / medical stores of Punjab , Pakistan : A road to demolition of public health. Heliyon [Internet]. 2021;7(November 2020):e07031. Available from: https://doi.org/10.1016/j.heliyon.2021.e07031
31. Volkow ND, McLellan AT. Opioid Abuse in Chronic Pain — Misconceptions and Mitigation Strategies. N Engl J Med. 2016;374(13):1253–63.
32. Aziz MM, Ji W, Masood I, Farooq M, Malik MZ, Chang J, et al. Patient satisfaction with community pharmacies services: A cross-sectional survey from Punjab; Pakistan. Int J Environ Res Public Health. 2018;15(12).
33. Moecker R, Weissenborn M, Klingenberg A, Wirbka L, Fuchs A, Eickhoff C, et al. Task sharing in an interprofessional medication management program – a survey of general practitioners and community pharmacists. BMC Health Serv Res. 2022;22:1–10.
34. Mubarak N, Hatah E, Aris MAM, Shafie AA, Zin CS. Consensus among healthcare stakeholders on a collaborative medication therapy management model for chronic diseases in Malaysia; A Delphi study. PLoS One. 2019;14(5):e0216563–e0216563.
35. Balough MM, Nwankpa S, Unni EJ. Readiness of Pharmacists Based in Utah About Pain Management and Opioid Dispensing. Pharm (Basel, Switzerland). 2019 Jan;7(1).
36. Mubarak N, Arif S, Irshad M, Aqeel RM, Khalid A, Ijaz UEB, et al. How are we educating future physicians and pharmacists in pakistan? A survey of the medical and pharmacy student's perception on learning and preparedness to assume future roles in antibiotic use and resistance. Antibiotics. 2021;10(10).
37. Renfro ML, Moczygemba LR, Baumgartner J, Baumgart G, Hill LG. Opioid-Related Education Provided by Continuing Education Divisions at US Pharmacy Schools. Am J Pharm Educ. 2020 Oct;84(10):ajpe8001.
38. Hohmeier KC, Cernasev A, Sensmeier M, Hall E, Webb K, Barenie R, et al. U.S. student pharmacist perceptions of the pharmacist's role in methadone for opioid use disorder: A qualitative study. SAGE Open Med. 2021;9:205031212110229.

For peer review only - http://bmjopen.bmj.com/site/about/guidelines.xhtml

"Are Pharmacists on the Frontlines of the Opioid Epidemic? A Cross Sectional Study of the Practices and Competencies of Community and Hospital Pharmacists in Punjab, Pakistan"

Naeem Mubarak,[1] Taheer Zahid,[2] Fatima Rahman Rana,[1] Umm-E-Barirah Ijaz,[1] Afshan Shabbir,[1] Mahrukh Manzoor,[1] Nahan Khan,[1] Minahil Arif,[1] Muhammad Mehroz Naeem,[1] Sabba Kanwal,[1] Nasira Saif-ur-Rehman,[3] Che Suraya Zin,[4] Khalid Mahmood,[5] Javaid Asgher,[1,6,7] Mohamed Hassan Elnaem[8*]

[1] Department of Pharmacy Practice, Faculty of Pharmaceutical Sciences, Lahore University of Biological & Applied Sciences, Lahore, Punjab, Pakistan.

[2] Manager, Servaid Pharmacy, 24-M Quaid-e-Azam Industrial Estate, Lahore, Punjab, Pakistan.

[3] Department of Pharmaceutics, Faculty of Pharmaceutical Sciences, Lahore University of Biological & Applied Sciences, Lahore, Punjab, Pakistan.

[4] Kulliyyah of Pharmacy, International Islamic University, Kuantan, Malaysia.

[5] Institute of Information Management, University of the Punjab, Lahore, Punjab, Pakistan.

[6] Lahore Medical & Dental College, Lahore, Punjab, Pakistan.

[7] Doctors Hospital & Medical Centre, Lahore, Punjab, Pakistan.

[8] School of Pharmacy and Pharmaceutical Sciences, Ulster University, Coleraine, United Kingdom.

Corresponding author:
Dr Mohamed Hassan Elnaem,
School of Pharmacy and Pharmaceutical Sciences, Ulster University, Coleraine, United Kingdom.
Email: m.elnaem@ulster.ac.uk
ORCiD: 0000-0003-0873-6541

For peer review only

DEMOGRAPHICS

Please ENCIRCLE/WRITE

- **Gender:** Male Female
- **Age (years)** ________ **Years of practice experience** _________
- **Employment status:** Full-time Part-time Intern
- **Type of community pharmacy:** Chain Independent Hospital
- **If pharmacy in hospital, type of hospital:** Public Private
- **Highest level of education:** PhD Masters Pharm-D/B-Pharm
- **Location of pharmacy:**
  - Lahore, Kasur, Nankana Sahib, Sheikhupura
  - Gujranwala, Gujrat, Hafizabad, MandiBahauddin, Narowal, Sialkot
  - Sargodha, Khushab, Mianwali, Bhakkar
  - Sahiwal, Pakpattan, Okara
  - Multan, Lodhran, Khanewal, Vehari
  - Faisalabad, Chiniot, Toba Tek Singh, Jhang
  - Rawalpindi, Jhelum, Chakwal, Attock
  - Bahawalpur, Bahawalnagar, Rahim Yar Khan
  - DG Khan, Layyah, Muzaffargarh, Rajanpur
- **Please encircle the appropriate:**

[Never = 1, rarely= 2, Sometimes= 3, Very often= 4, Almost always =5]

- I……………..make clinical notes in a journal/dispensing software (apart from narcotic register) to monitor ongoing opioids use.
  1 2 3 4 5
- I …...counsel patients on the possible side effects of opioids.
  1 2 3 4 5
- I …...counsel patients on opioids over dose risk.
  1 2 3 4 5
- I ……….refuse the supply of the opioids when in doubt of abuse
  1 2 3 4 5
- I………..notify police/regulatory bodies on the abuse of opioids
  1 2 3 4 5
- To clarify the opioids' prescription, I ………..call the physician

[Never = 1, rarely= 2, Sometimes= 3 Very often= 4 Almost always =5]

- **Have you completed any continuous professional development course in last ONE year?** YES NO
- **You completed your B.Pharm/Pharm-D degree from a ……….institute**
  Public Private
- **Your system of education in B.Pharm/Pharm-D was:**
  Annual Semester

OPIOIDS AND SOCIETY

1 **The "Opioid Epidemic" or "Opioid Crises" refers to the increase in the number of deaths and hospitalization:**
   a. Due to prescription opioid use
   b. Due to illicit(illegal) opioid use
   c. Due to opioid use for instant pain relief
   d. Due to both prescription and illicit(illegal) opioid use
   e. I don't know

2 **National Institute on Drug Abuse recommends the term _______ for a "substance or drug abuser"**
   a. Drug addict
   b. Patient
   c. Junkie
   d. Addicted baby
   e. I don't know

3 **In Pakistan, the highest number of opioid drug users belongs to:**
   a. KPK
   b. Punjab
   c. Balochistan
   d. Sindh
   e. I don't know

4 **In Pakistan, opioid misuse is more common among**
   a. Males
   b. Females
   c. Transgender
   d. No difference on the basis of gender
   e. I don't know

5 **In Pakistan, generally women abuse sedative and amphetamines, while, men favor _____**
   a. Methamphetamine and cannabis
   b. Cannabis and alcohol
   c. Sedative and opioids
   d. Cannabis and opioids
   e. I don't know

For peer review only

OPIOID PHYSIOLOGY

1 **Opioids produce analgesia, which indicates:**
   a. CNS depression
   b. CNS stimulation
   c. Both depression and stimulation
   d. No effect
   e. I don't know

2 **The increase in the sensitivity of pain receptors is called:**
   a. Analgesia
   b. Algesia
   c. Hyperalgesia
   d. Allodynia
   e. I don't know

3 **Beta endorphins are the most common endogenous ligands for the receptor:**
   a. Beta
   b. Delta
   c. Kappa
   d. Mu
   e. I don't know

4 **When activated, which one of the following opioid receptors <u>mainly</u> cause <u>both</u> analgesia and dysphoria?**
   a. Mu
   b. Kappa
   c. Delta
   d. Alpha
   e. I don't know

5 **When activated, which one of the following opioid receptors causes the <u>most</u> <u>respiratory</u> depression?**
   a. Mu
   b. Beta
   c. Kappa
   d. Delta
   e. I don't know

OPIOID PHARMACOLOGY

1 **The difference between <u>Opiates</u> and Opioids is**
   a. Opiates are natural, while Opioids are synthetic
   b. Opioids are natural, while Opiates are synthetic
   c. Opiates are semi-synthetic, while Opioids are synthetic
   d. Opioids are semi-synthetic, while Opiates are synthetic
   e. I don't know

2 **Majority of the opioid receptors belongs to:**
   a. Intracellular receptors
   b. Tyrosine Kinase receptors
   c. G-protein coupled receptors
   d. Enzyme linked receptors
   e. I don't know

3 **Tramadol is a __________ Mu receptor agonist**
   a. Strong
   b. Weak
   c. Moderate
   d. None of these
   e. I don't know

4 **At the injection site, Morphine causes localized pain and itching due to the release of:**
   a. Glutamine
   b. Serotonin
   c. Dopamine
   d. Histamine
   e. I don't know

5 **Which one of the following opioids is a mixed agonist-antagonist?**
   a. Morphine
   b. Fentanyl
   c. Codeine
   d. Pentazocine
   e. I don't know

For peer review only
For peer review only - http://bmjopen.bmj.com/site/about/guidelines.xhtml

OPIOID PHARMACOKINETICS AND DRUG INTERACTIONS

1 **Morphine undergoes _________ during phase-2 biotransformation**
   a. Methylation
   b. Acetylation
   c. Glucuronidation
   d. Oxidation
   e. I don't know

2 **Which one of the following opioids is safe to use in renal failure and does not require dose adjustment?**
   a. Buprenorphine
   b. Morphine
   c. Fentanyl
   d. Methadone
   e. I don't know

3 **Which one of the following opioids has the least CNS penetration?**
   a. Morphine
   b. Loperamide
   c. Codeine
   d. Fentanyl
   e. I don't know

4 **Opioids can cause significant respiratory depression when administered with:**
   a. Naloxone
   b. Naltrexone
   c. Anti-hypertensive
   d. Benzodiazepines
   e. I don't know

5 **Which of the following drugs when administered with tramadol can cause serotonin syndrome (increase in serotonin levels)?**
   a. Tricyclic anti-depressants
   b. Selective serotonin reuptake inhibitors
   c. Both
   d. None of these
   e. I don't know

THERAPEUTIC USE OF OPIOIDS

1 **The appropriate treatment for moderate pain as defined by WHO analgesic ladder is:**
   a. NSAIDS
   b. Strong opioids and NSAIDS
   c. Weak opioids and NSAIDS
   d. Weak opioids and strong opioids
   e. I don't know

2 **Which of the following opioids are used for opioid substitution therapy?**
   a. Meperidine and Methadone
   b. Oxycodone and Buprenorphine
   c. Fentanyl and Methadone
   d. Methadone and Buprenorphine
   e. I don't know

3 **Opioid dependence refers to:**
   a. Withdrawal symptoms when not taking the opioid
   b. Reduced response to an opioid with repeated use
   c. Persistent use of an opioid despite substantial harm and adverse consequences
   d. Overwhelming desire to continue taking the opioid and obtaining it by all means
   e. I don't know

4 **Which one of the following opioids is generally contraindicated in Myocardial Infarction?**
   a. Morphine
   b. Fentanyl
   c. Methadone
   d. Pentazocine
   e. I don't know

5 **Opioid dependence during pregnancy can be treated with:**
   a. Methadone
   b. Fentanyl
   c. Naloxone
   d. Morphine
   e. I don't know

For peer review only

For peer review only - http://bmjopen.bmj.com/site/about/guidelines.xhtml

OPIOID EDUCATION

1 **Pharmacist should educate the patient to change the fentanyl patch after:**
   a. 72 hours
   b. 48 hours
   c. 24 hours
   d. 6 hours
   e. I don't know

2 **The appropriate fate of fentanyl patch after use is:**
   a. Reuse the same patch
   b. Incinerate(fire) the patch
   c. Flush over (used or leftover patches)
   d. Give to someone else for use to prevent drug wastage
   e. I don't know

3 **Pharmacist should advise the patient to store opioids at:**
   a. Room temperature
   b. Refrigerator
   c. Freezer
   d. Any temperature
   e. I don't know

4 **Pharmacist should restrict methadone supply for:**
   a. 7 days
   b. 8 days
   c. 6 days
   d. 10 days
   e. I don't know

5 **In case of incorrect, ambiguous or incomplete prescription of opioids, pharmacist should:**
   a. Dispense medication as written
   b. Correct the prescription and dispense the medication
   c. Call to police
   d. Hold dispensing and clarify from the prescriber
   e. I don't know

OPIOID DISPENSING

1 **In Pakistan, as per Control of Narcotic Substances Act 1967, opioids come under:**
   a. Schedule A
   b. Schedule B
   c. Schedule C
   d. Schedule D
   e. I don't know

2 **Which one of the following is NOT the sign of opioid's intoxication?**
   a. Altered mental state
   b. Shallow breathing
   c. Irresponsiveness
   d. Fast breathing
   e. I don't know

3 **Pharmacist should dispense the take home doses of opioids in:**
   a. Child resistant containers
   b. Amber colored bottles
   c. Light colored containers
   d. Aluminum foil
   e. I don't know

4 **The record for the sale of opioids should be documented in the narcotic register with:**
   a. blue ink
   b. red ink
   c. green ink
   d. any color
   e. I don't know

5 **As per Punjab Drug Rules-2007, which one of the following is an important particular to mention in the narcotic register during the sale of opioids?**
   a. Lot number
   b. Expiry date
   c. Manufacturing date
   d. Batch number
   e. I don't know

For peer review only - http://bmjopen.bmj.com/site/about/guidelines.xhtml

For peer review only

OPIOIDS AND PAIN MANAGEMENT

1 **…is the pain due to injury to nerve and opioid of choice for this kind of pain is…………**
   a. Neuropathic, Methadone
   b. Psychogenic, Morphine
   c. Somatic, Methadone
   d. Visceral, Morphine
   e. I don't know

2 **Which one of the following opioids is the gold standard for the treatment of acute (severe, accidental) pain?**
   a. Methadone
   b. Fentanyl
   c. Morphine
   d. Tramadol
   e. I don't know

3 **Which one of the following is the opioid of choice in labor pain?**
   a. Meperidine
   b. Pentazocine
   c. Methadone
   d. Buprenorphine
   e. I don't know

4 **The Centers for Disease Control and Prevention recommends the use of opioid therapy in patients with acute (severe) pain for:**
   a. 7-14 days
   b. 3-7 days
   c. 14-28 days
   d. More than 1 month
   e. I don't know

5 **FDA approved opioid for treatment of acute pain in children is:**
   a. Morphine
   b. Codeine
   c. Fentanyl
   d. Methadone
   e. I don't know

COURSE OF OPIOID THERAPY

1 **Opioid taper, Opioid rotation, and Opioid Equianalgesic table refer to _____ , respectively:**
   a. Dose reduction over time, switching between opioids, equivalent to analgesic dose of morphine
   b. Switching between opioids, dose reduction over time , equivalent to analgesic dose of codeine
   c. Dose reduction over time, switching from opioids to non-opioids, equivalent to analgesic dose of morphine
   d. Dose reduction over time, switching between opioids, equivalent to analgesic dose of methadone
   e. I don't know

2 **The appropriate adult dose of Tramadol is:**
   a. 50-100mg for 4-6 hours PRN, with maximum dose up to 400mg/day
   b. 200-300mg for 4-6 hours PRN, with maximum dose up to 500mg/day
   c. 50-100mg for 4-6 hours PRN, with maximum dose up to 500mg/day
   d. 200-300mg for 4-6 hours PRN, with maximum dose up to 400mg/day
   e. I don't know

3 **As per equianalgesic table, _____ oral dose of tramadol is equivalent to 10mg of morphine**
   a. 100mg
   b. 50mg
   c. 200mg
   d. 150mg
   e. I don't know

4 **The indications for an opioid rotation are to:**
   a. Improve analgesia
   b. Reduce adverse effects
   c. Decrease opioid tolerance
   d. All of the above
   e. I don't know

5 **CDC guidelines recommend appropriate opioid tapering as the**
   a. 10% decrease in dose per month
   b. 20% decrease in dose per month
   c. 30% decrease in dose per month
   d. 40% decrease in dose per month
   e. I don't know

For peer review only - http://bmjopen.bmj.com/site/about/guidelines.xhtml

OPIOID MONITORING

1 **5A's of monitoring chronic pain are:**
   a. Activity, analgesia, adverse effects, aberrant behaviors, affect
   b. Activity, algesia, adverse effects, aberrant behaviors, assign
   c. Activity, algesia, adverse effects, adherent behaviors, affect
   d. Action, analgesia, adverse effects, aberrant behaviors, assign
   e. I don't know

2 **The bolus injection of morphine requires monitoring of respiratory rate after every:**
   a. 12 hours
   b. 6 hours
   c. 3 hours
   d. 24 hours
   e. I don't know

3 **Opioid therapy monitoring is recommended ……for patients at <u>low risk</u> of adverse effects:**
   a. Once every 3-6 months
   b. Twice every 3-6 months
   c. Monthly
   d. Weekly
   e. I don't know

4 **Opioid therapy monitoring is recommended ……..for patients at <u>high risk</u> of adverse effects:**
   a. Once every 3-6 months
   b. Twice every 3-6 months
   c. Monthly
   d. Weekly
   e. I don't know

5 **Chronic opioid therapy is the use of opioids daily or nearly daily for <u>at least</u>:**
   a. 30 days
   b. 90 days
   c. 60 days
   d. 180 days
   e. I don't know

OPIOID OVERDOSE TRAINING

1 **The maximum toxic dose of morphine in adult patients is:**
   a. 250mg/day
   b. 500mg/day
   c. 1000mg/day
   d. 100mg/day
   e. I don't know

2 **The usual dose of Naloxone for opioid toxicity is:**
   a. 0.4mg to 2mg IV at 2-3 min intervals
   b. 10 mg IV at 2-3 min intervals
   c. 2.5mg to 5mg at 2-3 min intervals
   d. 7.5 mg IV at 2-3 min intervals
   e. I don't know

3 **The FDA recommends administering IM Naloxone in the**
   a. Upper arm
   b. Buttock
   c. Veins
   d. Intrathecal
   e. I don't know

4 **Three key symptoms of opioid overdose triad include**:
   a. Pinpoint pupils, Slow breathing, Unconsciousness/non-responsiveness
   b. Dilated pupils, Shallow breathing, Unconsciousness/ non-responsiveness
   c. Pinpoint pupils, Fast breathing, Clammy skin
   d. Dilated pupils, Slow breathing, Limp body (difficulty in walk)
   e. I don't know

5 **Which one of the following opioids is resistant to Naloxone?**
   a. Buprenorphine
   b. Morphine
   c. Codeine
   d. Hydromorphone
   e. I don't know

For peer review only - http://bmjopen.bmj.com/site/about/guidelines.xhtml

For peer review only

"Are Pharmacists on the Frontlines of the Opioid Epidemic? A Cross Sectional Study of the Practices and Competencies of Community and Hospital Pharmacists in Punjab, Pakistan"

Naeem Mubarak,[1] Taheer Zahid,[2] Fatima Rahman Rana,[1] Umm-E-Barirah Ijaz,[1] Afshan Shabbir,[1] Mahrukh Manzoor,[1] Nahan Khan,[1] Minahil Arif,[1] Muhammad Mehroz Naeem,[1] Sabba Kanwal,[1] Nasira Saif-ur-Rehman,[3] Che Suraya Zin,[4] Khalid Mahmood,[5] Javaid Asgher,[1,6,7] Mohamed Hassan Elnaem[8*]

[1] Department of Pharmacy Practice, Faculty of Pharmaceutical Sciences, Lahore University of Biological & Applied Sciences, Lahore, Punjab, Pakistan.

[2] Manager, Servaid Pharmacy, 24-M Quaid-e-Azam Industrial Estate, Lahore, Punjab, Pakistan.

[3] Department of Pharmaceutics, Faculty of Pharmaceutical Sciences, Lahore University of Biological & Applied Sciences, Lahore, Punjab, Pakistan.

[4] Kulliyyah of Pharmacy, International Islamic University, Kuantan, Malaysia.

[5] Institute of Information Management, University of the Punjab, Lahore, Punjab, Pakistan.

[6] Lahore Medical & Dental College, Lahore, Punjab, Pakistan.

[7] Doctors Hospital & Medical Centre, Lahore, Punjab, Pakistan.

[8] School of Pharmacy and Pharmaceutical Sciences, Ulster University, Coleraine, United Kingdom.

Corresponding author:
Dr Mohamed Hassan Elnaem,
School of Pharmacy and Pharmaceutical Sciences, Ulster University, Coleraine, United Kingdom.
Email: m.elnaem@ulster.ac.uk
ORCiD: 0000-0003-0873-6541

For peer review only

STROBE Statement—checklist of items that should be included in reports of observational studies.

	Item No.	Recommendation	Page No.	Relevant text from manuscript
**Title and abstract**	1	(*a*) Indicate the study's design with a commonly used term in the title or the abstract	1	Line 1
		(*b*) Provide in the abstract an informative and balanced summary of what was done and what was found	2	Line 36-63
		**Introduction**		
Background/rationale	2	Explain the scientific background and rationale for the investigation being reported	4-5	Line 81-129
Objectives	3	State specific objectives, including any prespecified hypotheses	5	Line 128
		**Methods**		
Study design	4	Present key elements of study design early in the paper	5	Line 133
Setting	5	Describe the setting, locations, and relevant dates, including periods of recruitment, exposure, follow-up, and data collection	5-6	Line 132-149
Participants	6	(*a*) *Cohort study*—Give the eligibility criteria, and the sources and methods of selection of participants. Describe methods of follow-up<br>*Case-control study*—Give the eligibility criteria, and the sources and methods of case ascertainment and control selection. Give the rationale for the choice of cases and controls<br>*Cross-sectional study*—Give the eligibility criteria, and the sources and methods of selection of participants	7,16	Line 185-199, 440
		(*b*) *Cohort study*—For matched studies, give matching criteria and number of exposed and unexposed<br>*Case-control study*—For matched studies, give matching criteria and the number of controls per case	NA	NA
Variables	7	Clearly define all outcomes, exposures, predictors, potential confounders, and effect modifiers. Give diagnostic criteria, if applicable	8	Line 202-209
Data sources/ measurement	8*	For each variable of interest, give sources of data and details of methods of assessment (measurement). Describe comparability of assessment methods if there is more than one group	8	Line 202-209
Bias	9	Describe any efforts to address potential sources of bias	6	Line 147-149
Study size	10	Explain how the study size was arrived at	6	Line 143-146

For peer review only - http://bmjopen.bmj.com/site/about/guidelines.xhtml

Quantitative variables	11	Explain how quantitative variables were handled in the analyses. If applicable, describe which groupings were chosen and why	8,12	Line 202-209,273-277
Statistical methods	12	(*a*) Describe all statistical methods, including those used to control for confounding	8-13	Line 202-209,231-288
		(*b*) Describe any methods used to examine subgroups and interactions	8,12	Line 202-209,273-277
		(*c*) Explain how missing data were addressed	8	Line 199
		(*d*) *Cohort study*—If applicable, explain how loss to follow-up was addressed<br>*Case-control study*—If applicable, explain how matching of cases and controls was addressed.<br>*Cross-sectional study*—If applicable, describe analytical methods taking account of sampling strategy	5-6	Line 138-149
		(*e*) Describe any sensitivity analyses	NA	NA
		**Results**		
Participants	13*	(a) Report numbers of individuals at each stage of study—e.g., numbers potentially eligible, examined for eligibility, confirmed eligible, included in the study, completing follow-up, and analysed	5-6	Line 138-149
		(b) Give reasons for non-participation at each stage	8	Line 199
		(c) Consider use of a flow diagram	NA	NA
Descriptive data	14*	(a) Give characteristics of study participants (eg demographic, clinical, social) and information on exposures and potential confounders	9	Line 232-241
		(b) Indicate number of participants with missing data for each variable of interest	8	Line 199
		(c) *Cohort study*—Summarise follow-up time (eg, average and total amount)	NA	NA
Outcome data	15*	*Cohort study*—Report numbers of outcome events or summary measures over time	*NA*	*NA*
		Case-control study—Report numbers in each exposure category, or summary measures of exposure	*NA*	*NA*
		Cross-sectional study—Report numbers of outcome events or summary measures	8-13	Line 231-288
Main results	16	(*a*) Give unadjusted estimates and, if applicable, confounder-adjusted estimates and their precision (e.g., 95% confidence interval). Make clear which confounders were adjusted for and why they were included	8	Line 212-218
		(*b*) Report category boundaries when continuous variables were categorized	NA	NA
		(*c*) If relevant, consider translating estimates of relative risk into absolute risk for a meaningful time period	NA	NA

Continued on next page

For peer review only - http://bmjopen.bmj.com/site/about/guidelines.xhtml

Other analyses	17	Report other analyses done—e.g., analyses of subgroups and interactions, and sensitivity analyses	13	Line 279
		**Discussion**		
Key results	18	Summarise key results with reference to study objectives	13	Line 291
Limitations	19	Discuss limitations of the study, taking into account sources of potential bias or imprecision. Discuss both direction and magnitude of any potential bias	6,18	Line 147-149,410-417
Interpretation	20	Give a cautious overall interpretation of results considering objectives, limitations, multiplicity of analyses, results from similar studies, and other relevant evidence	13-18	Line 291-417
Generalisability	21	Discuss the generalisability (external validity) of the study results	3	Line 72,78
**Other information**				
Funding	22	Give the source of funding and the role of the funders for the present study and, if applicable, for the original study on which the present article is based	20	Line 471

*Give information separately for cases and controls in case-control studies and, if applicable, for exposed and unexposed groups in cohort and cross-sectional studies.

Note: An Explanation and Elaboration article discusses each checklist item and gives methodological background and published examples of transparent reporting. The STROBE checklist is best used in conjunction with this article (freely available on the Web sites of PLoS Medicine at http://www.plosmedicine.org/, Annals of Internal Medicine at http://www.annals.org/, and Epidemiology at http://www.epidem.com/). Information on the STROBE Initiative is available at www.strobe-statement.org.

For peer review only - http://bmjopen.bmj.com/site/about/guidelines.xhtml

LAHORE PHARMACY COLLEGE

(A PROJECT OF LMDC)
Affiliated with
UNIVERSITY OF HEALTH SCIENCES, LAHORE

Ref. No. LPC / ETH/26/09/22 Date: 23-09-2022

Dr. Naeem Mubarak,
Department of Pharmacy Practice,
Lahore Pharmacy College,
(A Project of LMDC).

SUBJECT: **ETHICAL APPROVAL CERTIFICATE**

Dear Dr. Naeem Mubarak,

Apropos of the ethical review application (ref: NM/03//22), after a detailed review of the ethical aspects of the research project mentioned below, the Research Ethics Committee of Lahore Pharmacy College grants ethical approval for the implementation of the study.

- **Research Title:** "Evaluating competencies of Community Pharmacists when supplying opioids. A cross sectional study in Punjab, Pakistan".
- **Principal Supervisor:** Dr. Naeem Mubarak
- **Co-Supervisors:** Prof. Dr. Nasira Saif ur Rehman

This ethical approval certificate is valid from **23rd Sep. 2022** to **23rd Sep. 2023.**

Yours Truly,

Dr. Asad Majeed Khan (PhD)
Chairperson,
Research Ethics Committee,
Lahore Pharmacy College,
University of Health Sciences,
Lahore, Pakistan.
asad.majeed@lmdc.edu.pk

For peer review only - http://bmjopen.bmj.com/site/about/guidelines.xhtml

"Are Pharmacists on the Frontlines of the Opioid Epidemic? A Cross Sectional Study of the Practices and Competencies of Community and Hospital Pharmacists in Punjab, Pakistan"

Naeem Mubarak,[1] Taheer Zahid,[2] Fatima Rahman Rana,[1] Umm-E-Barirah Ijaz,[1] Afshan Shabbir,[1] Mahrukh Manzoor,[1] Nahan Khan,[1] Minahil Arif,[1] Muhammad Mehroz Naeem,[1] Sabba Kanwal,[1] Nasira Saif-ur-Rehman,[3] Che Suraya Zin,[4] Khalid Mahmood,[5] Javaid Asgher,[1,6,7] Mohamed Hassan Elnaem[8*]

[1] Department of Pharmacy Practice, Faculty of Pharmaceutical Sciences, Lahore University of Biological & Applied Sciences, Lahore, Punjab, Pakistan.

[2] Manager, Servaid Pharmacy, 24-M Quaid-e-Azam Industrial Estate, Lahore, Punjab, Pakistan.

[3] Department of Pharmaceutics, Faculty of Pharmaceutical Sciences, Lahore University of Biological & Applied Sciences, Lahore, Punjab, Pakistan.

[4] Kulliyyah of Pharmacy, International Islamic University, Kuantan, Malaysia.

[5] Institute of Information Management, University of the Punjab, Lahore, Punjab, Pakistan.

[6] Lahore Medical & Dental College, Lahore, Punjab, Pakistan.

[7] Doctors Hospital & Medical Centre, Lahore, Punjab, Pakistan.

[8] School of Pharmacy and Pharmaceutical Sciences, Ulster University, Coleraine, United Kingdom.

Corresponding author:
Dr Mohamed Hassan Elnaem,
School of Pharmacy and Pharmaceutical Sciences, Ulster University, Coleraine, United Kingdom.
Email: m.elnaem@ulster.ac.uk
ORCiD: 0000-0003-0873-6541

For peer review only

S1 Table Cronbach's alpha value against each of the eleven opioid competencies

Sr.	Competency	Alpha*
**1.**	Opioids & Society	0.654
**2.**	Opioids' Physiology	0.800
**3.**	Opioids' Pharmacology	0.776
**4.**	Opioids' Pharmacokinetic & Drug Interaction	0.826
**5.**	Therapeutic use of Opioids	0.746
**6.**	Opioids' Education	0.740
**7.**	Opioids Dispensing	0.833
**8.**	Opioids & Pain Management	0.852
**9.**	Course of Opioid Therapy	0.844
**10.**	Opioids' Monitoring	0.871
**11.**	Opioid Overdose Management	0.852

*** Cronbach's alpha coefficient test**

For peer review only

"Are Pharmacists on the Frontlines of the Opioid Epidemic? A Cross Sectional Study of the Practices and Competencies of Community and Hospital Pharmacists in Punjab, Pakistan"

Naeem Mubarak,[1] Taheer Zahid,[2] Fatima Rahman Rana,[1] Umm-E-Barirah Ijaz,[1] Afshan Shabbir,[1] Mahrukh Manzoor,[1] Nahan Khan,[1] Minahil Arif,[1] Muhammad Mehroz Naeem,[1] Sabba Kanwal,[1] Nasira Saif-ur-Rehman,[3] Che Suraya Zin,[4] Khalid Mahmood,[5] Javaid Asgher,[1,6,7] Mohamed Hassan Elnaem[8*]

[1] Department of Pharmacy Practice, Faculty of Pharmaceutical Sciences, Lahore University of Biological & Applied Sciences, Lahore, Punjab, Pakistan.

[2] Manager, Servaid Pharmacy, 24-M Quaid-e-Azam Industrial Estate, Lahore, Punjab, Pakistan.

[3] Department of Pharmaceutics, Faculty of Pharmaceutical Sciences, Lahore University of Biological & Applied Sciences, Lahore, Punjab, Pakistan.

[4] Kulliyyah of Pharmacy, International Islamic University, Kuantan, Malaysia.

[5] Institute of Information Management, University of the Punjab, Lahore, Punjab, Pakistan.

[6] Lahore Medical & Dental College, Lahore, Punjab, Pakistan.

[7] Doctors Hospital & Medical Centre, Lahore, Punjab, Pakistan.

[8] School of Pharmacy and Pharmaceutical Sciences, Ulster University, Coleraine, United Kingdom.

Corresponding author:
Dr Mohamed Hassan Elnaem,
School of Pharmacy and Pharmaceutical Sciences, Ulster University, Coleraine, United Kingdom.
Email: m.elnaem@ulster.ac.uk
ORCiD: 0000-0003-0873-6541

For peer review only

S2 Table Details the individual competency scores against different variables.

Variable	Category	Opioids & Society (C1)		Opioids' Physiology (C2)		Opioids' Pharmacology (C3)		Opioids' Pharmacokinetic & Drug Interaction (C4)		Therapeutic use of Opioids (C5)		Opioids' Education (C6)		Opioids Dispensing (C7)		Opioids & Pain Management (C8)		Course of Opioid Therapy (C9)		Opioids' Use monitoring (C10)		Opioid Overdose Management (C11)	
		**Mean (SD)**	**t (p-value)**	**Mean (SD)**	**t (p-value)**	**Mean (SD)**	**t (p-value)**	**Mean (SD)**	**t (p-value)**	**Mean (SD)**	**t (p-value)**	**Mean (SD)**	**t (p-value)**	**Mean (SD)**	**t (p-value)**	**Mean (SD)**	**t (p-value)**	**Mean (SD)**	**t (p-value)**	**Mean (SD)**	**t (p-value)**	**Mean (SD)**	**t (p-value)**
**Gender**	**Male**	1.45 (0.956)	-0.160 (0.873)	2.05 (1.353)	-0.966 (0.335)	1.95 (1.389)	-1.649 (0.100)	1.88 (1.280)	0.250 (0.803)	1.36 (1.198)	-1.329 (0.184)	2.17 (1.205)	-1.030 (0.303)	2.18 (1.352)	-1.200 (0.231)	1.78 (1.115)	0.048 (0.962)	1.91 (1.376)	1.620 (0.106)	1.42 (1.049)	-0.251 (0.802)	1.49 (1.316)	-0.589 (0.556)
	**Female**	1.46 (0.957)		2.15 (1.388)		2.12 (1.442)		1.86 (1.239)		1.48 (1.244)		2.27 (1.219)		2.30 (1.307)		1.77 (1.083)		1.75 (1.374)		1.44 (1.079)		1.55 (1.286)	
**System of assessment in the Pharm-D degree program**	**Annual**	1.49 (0.995)	1.054 (0.292)	2.17 (1.379)	1.639 (0.102)	2.05 (1.409)	0.826 (0.409)	1.90 (1.257)	0.608 (0.543)	1.39 (1.215)	-0.364 (0.716)	2.22 (1.210)	0.225 (0.822)	2.28 (1.285)	1.104 (0.270)	1.85 (1.098)	1.869 (0.062)	1.87 (1.474)	0.348 (0.728)	1.46 (1.074)	0.735 (0.462)	1.57 (1.350)	1.235 (0.219)
	**Semester**	1.42 (0.919)		2.01 (1.351)		1.97 (1.411)		1.85 (1.273)		1.42 (1.216)		2.20 (1.211)		2.17 (1.379)		1.71 (1.104)		1.84 (1.287)		1.40 (1.047)		1.46 (1.263)	
**Type of institute**	**Public**	1.49 (0.955)	1.144 (0.253)	2.16 (1.339)	1.625 (0.105)	2.02 (1.372)	0.248 (0.805)	1.96 (1.322)	2.066 **(0.039)** *	1.42 (1.244)	0.461 (0.645)	2.21 (1.221)	0.065 (0.948)	1.22 (1.351)	-0.082 (0.935)	1.89 (1.118)	2.939 **(0.003)** *	1.92 (1.479)	1.315 (0.189)	1.49 (1.096)	1.696 (0.090)	1.58 (1.337)	1.573 (0.116)
	**Private**	1.41 (0.956)		2.01 (1.390)		2.00 (1.196)		1.78 (1.196)		1.38 (1.185)		1.21 (1.200)		1.22 (1.322)		1.66 (1.075)		1.79 (1.258)		1.36 (0.016)		1.43 (0.268)	
**Highest level of education**	**Masters**	1.57 (0.960)	1.666 (0.096)	2.43 (1.353)	3.475 **(0.001)** *	2.24 (1.469)	2.259 **(0.024)** *	2.02 (1.351)	1.618 (0.106)	1.85 (1.244)	5.118 **(0.000)** *	2.41 (1.288)	2.173 **(0.031)** *	2.32 (1.371)	1.004 (0.316)	1.92 (1.137)	1.877 (0.061)	2.08 (1.494)	2.189 **(0.030)** *	1.62 (1.065)	2.539 **(0.011)** *	1.55 (1.315)	0.413 (0.680)
	**Pharm-D/ B-Pharm**	1.43 (0.953)		2.00 (1.357)		1.95 (1.390)		1.84 (1.241)		1.30 (1.184)		2.16 (1.186)		2.20 (1.327)		1.74 (1.092)		1.80 (1.341)		1.38 (0.053)		1.50 (1.303)	
**Have you completed any continuous professional development courses in last TWO years?**	**Yes**	1.53 (0.966)	1.230 (0.219)	2.03 (1.385)	-0.586 (0.558)	2.01 (1.526)	0.012 (0.991)	1.94 (1.317)	0.851 (0.395)	1.49 (1.197)	1.008 (0.314)	2.20 (1.239)	-0.103 (0.896)	2.25 (1.440)	0.327 (0.744)	1.76 (1.108)	-0.154 (0.878)	1.75 (1.445)	-1.180 (0.238)	1.40 (1.051)	-0.444 (0.658)	1.33 (1.259)	-2.113 **(0.035)** *
	**No**	1.43 (0.952)		2.10 (1.360)		2.01 (1.375)		1.85 (1.250)		1.38 (1.220)		2.21 (1.203)		2.21 (1.305)		1.78 (1.102)		1.88 (1.356)		1.44 (1.063)		1.56 (1.314)	
**Employment status**	**Full-time**	1.50 (0.955)	2.520 **(0.012)** *	2.14 (1.350)	2.211 **(0.027)** *	2.08 (1.398)	2.581 **(0.010)** *	1.96 (1.264)	3.768 **(0.000)** *	1.46 (1.228)	2.412 **(0.017)** *	2.29 (1.196)	3.483 **(0.001)** *	2.31 (1.350)	3.875 **(0.000)** *	1.82 (1.094)	2.116 **(0.035)** *	1.91 (1.390)	2.162 **(0.031)** *	1.50 (1.076)	3.803 **(0.000)** *	1.55 (1.324)	1.537 (0.125)
	**Part-time**	1.29 (0.941)		1.88 (1.405)		1.76 (1.428)		1.55 (1.219)		1.21 (1.150)		1.92 (1.220)		1.89 (1.232)		1.62 (1.123)		1.65 (1.310)		1.17 (0.958)		1.38 (1.229)	
**If pharmacy is in hospital, type of hospital:**	**Public**	1.67 (0.943)	2.628 **(0.009)** *	2.34 (1.344)	1.417 (0.157)	2.17 (1.429)	0.783 (0.434)	2.02 (1.490)	1.053 (0.293)	1.59 (1.269)	1.242 (0.215)	2.20 (1.292)	-0.282 (0.778)	1.91 (1.233)	-1.126 (0.261)	1.95 (1.181)	1.056 (0.292)	2.15 (1.586)	1.719 (0.087)	1.48 (1.023)	0.620 (0.536)	1.58 (1.306)	0.372 (0.710)
	**Private**	1.35 (0.988)		2.10 (1.284)		2.02 (1.475)		1.84 (1.252)		1.39 (1.195)		2.25 (1.323)		2.10 (1.367)		1.79 (1.043)		1.84 (1.345)		1.39 (1.167)		1.52 (1.253)	
**Type of pharmacy practice**	**Community**	1.39 (0.944)	-2.389 **(0.017)** *	1.99 (1.379)	-2.620 **(0.009)** *	1.95 (1.389)	-1.598 (0.111)	1.83 (1.172)	-1.322 (0.187)	1.34 (1.194)	-2.033 **(0.042)** *	2.20 (1.159)	-0.174 (0.862)	2.36 (1.349)	3.847 **(0.000)** *	1.71 (1.078)	-2.312 **(0.021)** *	1.75 (1.283)	-2.803 **(0.005)** *	1.41 (1.054)	-0.422 (0.673)	1.48 (1.315)	-0.848 (0.397)
	**Hospital**	1.56 (0.968)		2.26 (1.327)		2.12 (1.443)		1.96 (1.416)		1.52 (1.246)		2.22 (1.300)		1.97 (1.279)		1.90 (1.138)		2.05 (1.515)		1.45 (1.071)		1.56 (1.287)	

***Independent t-test, p-value (≥0.05) Bold values indicate statistical significance.**
SD= standard deviation, C= competency

"**Are Pharmacists on the Frontlines of the Opioid Epidemic? A Cross Sectional Study of the Practices and Competencies of Community and Hospital Pharmacists in Punjab, Pakistan**"

Naeem Mubarak,[1] Taheer Zahid,[2] Fatima Rahman Rana,[1] Umm-E-Barirah Ijaz,[1] Afshan Shabbir,[1] Mahrukh Manzoor,[1] Nahan Khan,[1] Minahil Arif,[1] Muhammad Mehroz Naeem,[1] Sabba Kanwal,[1] Nasira Saif-ur-Rehman,[3] Che Suraya Zin,[4] Khalid Mahmood,[5] Javaid Asgher,[1,6,7] Mohamed Hassan Elnaem[8*]

[1] Department of Pharmacy Practice, Faculty of Pharmaceutical Sciences, Lahore University of Biological & Applied Sciences, Lahore, Punjab, Pakistan.

[2] Manager, Servaid Pharmacy, 24-M Quaid-e-Azam Industrial Estate, Lahore, Punjab, Pakistan.

[3] Department of Pharmaceutics, Faculty of Pharmaceutical Sciences, Lahore University of Biological & Applied Sciences, Lahore, Punjab, Pakistan.

[4] Kulliyyah of Pharmacy, International Islamic University, Kuantan, Malaysia.

[5] Institute of Information Management, University of the Punjab, Lahore, Punjab, Pakistan.

[6] Lahore Medical & Dental College, Lahore, Punjab, Pakistan.

[7] Doctors Hospital & Medical Centre, Lahore, Punjab, Pakistan.

[8] School of Pharmacy and Pharmaceutical Sciences, Ulster University, Coleraine, United Kingdom.

Corresponding author:
Dr Mohamed Hassan Elnaem,
School of Pharmacy and Pharmaceutical Sciences, Ulster University, Coleraine, United Kingdom.
Email: m.elnaem@ulster.ac.uk
ORCiD: 0000-0003-0873-6541

For peer review only

S3 Table Details the individual competency scores against different variables

Variable	Category	Opioids & Society (C1)		Opioids' Physiology (C2)		Opioids' Pharmacology (C3)		Opioids' Pharmacokinetic & Drug Interaction (C4)		Therapeutic use of Opioids (C5)		Opioids' Education (C6)		Opioids Dispensing (C7)		Opioids & Pain Management (C8)		Course of Opioid Therapy (C9)		Opioids' Use monitoring (C10)		Opioid Overdose Management (C11)	
		Mean (SD)	t (p-value)	Mean (SD)	t (p-value)	Mean (SD)	t (p-value)	Mean (SD)	t (p-value)	Mean (SD)	t (p-value)	Mean (SD)	t (p-value)	Mean (SD)	t (p-value)	Mean (SD)	t (p-value)	Mean (SD)	t (p-value)	Mean (SD)	t (p-value)	Mean (SD)	t (p-value)
Gender	Male	1.45 (0.956)	-0.160 (0.873)	2.05 (1.353)	-0.966 (0.335)	1.95 (1.389)	-1.649 (0.100)	1.88 (1.280)	0.250 (0.803)	1.36 (1.198)	-1.329 (0.184)	2.17 (1.205)	-1.030 (0.303)	2.18 (1.352)	-1.200 (0.231)	1.78 (1.115)	0.048 (0.962)	1.91 (1.376)	1.620 (0.106)	1.42 (1.049)	-0.251 (0.802)	1.49 (1.316)	-0.589 (0.556)
	Female	1.46 (0.957)		2.15 (1.388)		2.12 (1.442)		1.86 (1.239)		1.48 (1.244)		2.27 (1.219)		2.30 (1.307)		1.77 (1.083)		1.75 (1.374)		1.44 (1.079)		1.55 (1.286)	
System of assessment in the Pharm-D degree program	Annual	1.49 (0.995)	1.054 (0.292)	2.17 (1.379)	1.639 (0.102)	2.05 (1.409)	0.826 (0.409)	1.90 (1.257)	0.608 (0.543)	1.39 (1.215)	-0.364 (0.716)	2.22 (1.210)	0.225 (0.822)	2.28 (1.285)	1.104 (0.270)	1.85 (1.098)	1.869 (0.062)	1.87 (1.474)	0.348 (0.728)	1.46 (1.074)	0.735 (0.462)	1.57 (1.350)	1.235 (0.219)
	Semester	1.42 (0.919)		2.01 (1.351)		1.97 (1.411)		1.85 (1.273)		1.42 (1.216)		2.20 (1.211)		2.17 (1.379)		1.71 (1.104)		1.84 (1.287)		1.40 (1.047)		1.46 (1.263)	
Type of institute	Public	1.49 (0.955)	1.144 (0.253)	2.16 (1.339)	1.625 (0.105)	2.02 (1.372)	0.248 (0.805)	1.96 (1.322)	2.066 **(0.039)** *	1.42 (1.244)	0.461 (0.645)	2.21 (1.221)	0.065 (0.948)	1.22 (1.351)	-0.082 (0.935)	1.89 (1.118)	2.939 **(0.003)** *	1.92 (1.479)	1.315 (0.189)	1.49 (1.096)	1.696 (0.090)	1.58 (1.337)	1.573 (0.116)
	Private	1.41 (0.956)		2.01 (1.390)		2.00 (1.196)		1.78 (1.196)		1.38 (1.185)		1.21 (1.200)		1.22 (1.322)		1.66 (1.075)		1.79 (1.258)		1.36 (0.016)		1.43 (0.268)	
Highest level of education	Masters	1.57 (0.960)	1.666 (0.096)	2.43 (1.353)	3.475 **(0.001)** *	2.24 (1.469)	2.259 **(0.024)** *	2.02 (1.351)	1.618 (0.106)	1.85 (1.244)	5.118 **(0.000)** *	2.41 (1.288)	2.173 **(0.031)** *	2.32 (1.371)	1.004 (0.316)	1.92 (1.137)	1.877 (0.061)	2.08 (1.494)	2.189 **(0.030)** *	1.62 (1.065)	2.539 **(0.011)** *	1.55 (1.315)	0.413 (0.680)
	Pharm-D/ B-Pharm	1.43 (0.953)		2.00 (1.357)		1.95 (1.390)		1.84 (1.241)		1.30 (1.184)		2.16 (1.186)		2.20 (1.327)		1.74 (1.092)		1.80 (1.341)		1.38 (0.053)		1.50 (1.303)	
Have you completed any continuous professional development courses in last TWO years?	Yes	1.53 (0.966)	1.230 (0.219)	2.03 (1.385)	-0.586 (0.558)	2.01 (1.526)	0.012 (0.991)	1.94 (1.317)	0.851 (0.395)	1.49 (1.197)	1.008 (0.314)	2.20 (1.239)	-0.103 (0.896)	2.25 (1.440)	0.327 (0.744)	1.76 (1.108)	-0.154 (0.878)	1.75 (1.445)	-1.180 (0.238)	1.40 (1.051)	-0.444 (0.658)	1.33 (1.259)	-2.113 **(0.035)** *
	No	1.43 (0.952)		2.10 (1.360)		2.01 (1.375)		1.85 (1.250)		1.38 (1.220)		2.21 (1.203)		2.21 (1.305)		1.78 (1.102)		1.88 (1.356)		1.44 (1.063)		1.56 (1.314)	
Employment status	Full-time	1.50 (0.955)	2.520 **(0.012)** *	2.14 (1.350)	2.211 **(0.027)** *	2.08 (1.398)	2.581 **(0.010)** *	1.96 (1.264)	3.768 **(0.000)** *	1.46 (1.228)	2.412 **(0.017)** *	2.29 (1.196)	3.483 **(0.001)** *	2.31 (1.350)	3.875 **(0.000)** *	1.82 (1.094)	2.116 **(0.035)** *	1.91 (1.390)	2.162 **(0.031)** *	1.50 (1.076)	3.803 **(0.000)** *	1.55 (1.324)	1.537 (0.125)
	Part-time	1.29 (0.941)		1.88 (1.405)		1.76 (1.428)		1.55 (1.219)		1.21 (1.150)		1.92 (1.220)		1.89 (1.232)		1.62 (1.123)		1.65 (1.310)		1.17 (0.958)		1.38 (1.229)	
If pharmacy is in hospital, type of hospital:	Public	1.67 (0.943)	2.628 **(0.009)** *	2.34 (1.344)	1.417 (0.157)	2.17 (1.429)	0.783 (0.434)	2.02 (1.490)	1.053 (0.293)	1.59 (1.269)	1.242 (0.215)	2.20 (1.292)	-0.282 (0.778)	1.91 (1.233)	-1.126 (0.261)	1.95 (1.181)	1.056 (0.292)	2.15 (1.586)	1.719 (0.087)	1.48 (1.023)	0.620 (0.536)	1.58 (1.306)	0.372 (0.710)
	Private	1.35 (0.988)		2.10 (1.284)		2.02 (1.475)		1.84 (1.252)		1.39 (1.195)		2.25 (1.323)		2.10 (1.367)		1.79 (1.043)		1.84 (1.345)		1.39 (1.167)		1.52 (1.253)	
Type of pharmacy practice	Community	1.39 (0.944)	-2.389 **(0.017)** *	1.99 (1.379)	-2.620 **(0.009)** *	1.95 (1.389)	-1.598 (0.111)	1.83 (1.172)	-1.322 (0.187)	1.34 (1.194)	-2.033 **(0.042)** *	2.20 (1.159)	-0.174 (0.862)	2.36 (1.349)	3.847 **(0.000)** *	1.71 (1.078)	-2.312 **(0.021)** *	1.75 (1.283)	-2.803 **(0.005)** *	1.41 (1.054)	-0.422 (0.673)	1.48 (1.315)	-0.848 (0.397)
	Hospital	1.56 (0.968)		2.26 (1.327)		2.12 (1.443)		1.96 (1.416)		1.52 (1.246)		2.22 (1.300)		1.97 (1.279)		1.90 (1.138)		2.05 (1.515)		1.45 (1.071)		1.56 (1.287)	

***Independent t-test, p-value (≥0.05) Bold values indicate statistical significance.**
SD= standard deviation, C= competency
